# Issues in the Adoption of Online Medical Care: Cross-Sectional Questionnaire Survey

**DOI:** 10.2196/64159

**Published:** 2024-11-01

**Authors:** Yuka Sugawara, Yosuke Hirakawa, Masao Iwagami, Haruo Kuroki, Shuhei Mitani, Ataru Inagaki, Hiroki Ohashi, Mitsuru Kubota, Soichi Koike, Rie Wakimizu, Masaomi Nangaku

**Affiliations:** 1 Division of Nephrology and Endocrinology The University of Tokyo Tokyo Japan; 2 Department of Health Services Research, Institute of Medicine University of Tsukuba Ibaraki Japan; 3 Clinic Pauroom Tokyo Japan; 4 Department of Education, College of Education, Psychology and Human Studies Aoyama Gakuin University Tokyo Japan; 5 TAMA Family Clinic Tokyo Japan; 6 Department of General Pediatrics and Interdisciplinary Medicine National Center for Child Health and Development Tokyo Japan; 7 Division of Health Policy and Management, Center for Community Medicine Jichi Medical University Tochigi Japan; 8 Department of Child Health and Development Nursing, Institute of Medicine University of Tsukuba Ibaraki Japan

**Keywords:** telemedicine, online medical care, telehealth, eHealth, mobile phone

## Abstract

**Background:**

Telemedicine, or online medical care, has gained considerable attention worldwide. However, it has not been widely adopted in Japan, and the detailed status of received and provided online medical care and the reasons for its lack of popularity remain unknown.

**Objective:**

This study aims to investigate the current status of online medical care in Japan and the factors limiting its adoption from the perspective of both patients receiving and medical professionals providing online medical care.

**Methods:**

In total, 2 nationwide questionnaire surveys were conducted. The first survey, targeting both patients and healthy individuals, screened approximately 40,000 participants among 13 million people. The participants were selected to match the age distribution of the Japanese population based on government data, and their online medical care experience and medical visit status were recorded. To further investigate online medical care use and satisfaction, a web-based survey was conducted with 15% (6000/40,000) of the screened participants. The second survey, targeting medical professionals, was administered to a physician, a nurse, and a member of the administrative staff in each of 4900 randomly selected medical facilities to inquire about their online medical care practices and impressions. In addition, both surveys investigated the factors limiting online medical care expansion in Japan.

**Results:**

The response rates among patients and healthy individuals targeted for the screening and main surveys were 92.5% (36,998/40,000) and ˃80% (1312/1478, 88.77%; 1281/1522, 84.17%; 404/478, 84.5%; and 2226/2522, 88.26% in 4 survey groups), respectively. The survey of medical professionals yielded 1552 responses (n=618, 39.82% physicians; n=428, 27.58% nurses; n=506, 32.6% administrative staff). Although the facility-level response rate was low (794/4900, 16.2%), some facility categories had relatively high response rates. Only 5.29% (1956/36,998) of the patients and healthy individuals had online medical care experience. When there were more hospitals nearby and they felt it was more work to see a physician in person, they were more likely to use online medical care (more nearby hospitals: adjusted odds ratio [aOR] 1.33, 95% CI 1.18-1.50; more work: aOR 1.48, 95% CI 1.35-1.63 per survey response point in the patient group). Similarly, these factors were substantially associated with satisfaction (more nearby hospitals: aOR 1.40, 95% CI 1.14-1.73; more work: aOR 1.50, 95% CI 1.27-1.76 per survey response point in the patient group). In both surveys, the most frequently selected factor preventing the widespread use of online medical care was patients’ need to switch to face-to-face medical care for mandatory tests and procedures. Inadequate awareness of and education on online medical care were also frequently selected.

**Conclusions:**

Our nationwide surveys provided insights into the current status of online medical care in Japan and simultaneously identified several problems and issues related to it, which will be useful in promoting its wider adoption.

## Introduction

### Background

“Telemedicine” involves the delivery of all types of medical, diagnostic, and treatment-related services using telecommunication technologies [1]. This includes various medical processes such as conducting diagnostic tests, closely monitoring patient progress after treatment or therapy, and facilitating access to specialists who may not be geographically close to the patient. Similar terms for telemedicine include “telehealth” and “eHealth,” which are broader than telemedicine and often encompass digital app to support self-management. In Japan, the term “online medical care” is used, which is defined by the government more specifically than the term “telemedicine” as follows: the act of examining and diagnosing a patient, communicating the results of the diagnosis, and prescribing medical treatment in real time between a physician and a patient via information and telecommunications technology [2]. This definition emphasizes real-time communication between physicians and patients.

Telemedicine, including online medical care, has steadily evolved over time; however, the COVID-19 pandemic substantially accelerated its adoption and implementation worldwide. An analysis using Google Trends data revealed a sharp increase in public interest in telemedicine in April 2020 after the onset of the COVID-19 pandemic [3]. The number of telemedicine practices in the United States has been increasing rapidly since March 2020 with the expansion of public health insurance coverage [4]. The US Food and Drug Administration launched the Digital Health Center of Excellence in September 2020, which focuses on the development of various technologies such as mobile health devices, medical device software, and wearable technology. The United Kingdom and other European countries have been actively promoting telemedicine services through their governments, and telemedicine has become more widespread since the COVID-19 pandemic in many countries [5-8].

However, despite this global trend, Japan has not widely adopted telemedicine. Previously, telemedicine was highly restricted and not reimbursable in Japan; however, since 2018, online medical care medication expenses for specific diseases have been reimbursed. Despite deregulation and reimbursement revisions in Japan during the COVID-19 pandemic to promote online medical care [7], its implementation remains limited at many facilities and for many patients.

While online medical care is assumed to have the advantage of increasing convenience for patients, there are many unknowns, including whether it is convenient for medical institutions, whether accurate medical care can be provided, and to what extent patients are willing to use it. In clarifying these issues, it is also desirable to understand what types of facilities have offered online medical care the most, what types of patients have received online medical care, and in what types of situations patients were most satisfied with online medical care.

### Objectives

To understand the current situation following the COVID-19 pandemic and identify issues related to online medical care, we conducted a nationwide questionnaire survey of the 2 largest relevant populations: patients and healthy individuals and health care professionals. The primary objective of this study was to identify the key factors that limit the adoption of online medical care.

## Methods

### Study Design

In total, 2 nationwide questionnaire surveys were conducted at approximately the same time to analyze the actual status of online medical care and the potential barriers to online medical care expansion in Japan from the perspective of both patients and healthy individuals who receive online medical care and medical professionals who provide it. This study was registered with University hospital Medical Information Network- Clinical Trials Registry before initiation (registration ID: UMIN000051219).

### Development of Questionnaires

To develop the questionnaires, interviews were conducted at 2 sites with patients, health care professionals (physicians and nurses), and administrative staff members regarding their experiences with telemedicine, its advantages, and possible reasons why it is not currently desired or promoted. The interviews with health care professionals or administrative staff members were conducted in person or web-based, whereas patients were exclusively interviewed in person. No personal information was obtained from the participants.

The primary objective of this study was to determine the actual status of online medical care in Japan. In the patient group, we investigated whether they had experienced online medical care (a binary variable) and their level of satisfaction (on a 4-point scale) to determine the experience rate and the level of satisfaction of those who had experienced online medical care. In addition, factors related to the experience of online medical care or the satisfaction with online medical care (as outcome) were analyzed, including sex, age (surveyed as an integer and used as a binary variable for whether the patient was aged ≥60 years), number of nearby medical facilities (3 levels), and perceived effort involved in seeing a physician (4 levels; as exposure factors). In the group of health care professionals, the rate of online medical care implementation was examined overall, by type of facility, and by use of electronic medical records (EMRs).

The second objective of this study was to identify issues of online medical care. To this end, we obtained responses regarding factors that prevent the further spread of online medical care. In addition, as data to reinforce this, responses regarding the various patient burdens associated with medical visits (online medical care vs face-to-face medical care on a 5-point scale) and the ease of performing various medical procedures (online medical care vs face-to-face medical care on a 5-point scale) in the medical professional group were also surveyed.

On the basis of the summary of these interviews and previous findings, 2 questionnaires were developed and refined for patients and healthy individuals and for medical professionals. The final version of each questionnaire (in Japanese and English) is provided in Multimedia Appendices 1-4. The survey of patients and healthy individuals consisted of six parts: (1) background of respondents, (2) online medical care use and satisfaction among those with online medical care experience, (3) online medical care use prediction among those without online medical care experience, (4) various patient burdens, (5) comparison of face-to-face medical care and online medical care, and (6) factors impeding the adoption of online medical care. The survey of medical professionals consisted of seven parts: (1) background of respondent facilities and respondents, (2) status of online medical care use and its issues, (3) various patient burdens, (4) ease of performing various clinical procedures, (5) medical facilities’ financial burdens and medical fees with online medical care, (6) appropriate patients for online medical care, and (7) factors hindering the adoption of online medical care. Usability and technical functionality were tested by the multiple research team members before fielding the questionnaire.

### Survey of Patients and Healthy Individuals

To be eligible for the screening survey, respondents must be members of the Freeasy panel (iBRIDGE) [9], an internet-based survey panel of 13 million people. It consists of people who have registered with the site or application for the purpose of accumulating points and exchanging them for cash or e-money. In addition, to participate in the subsequent main survey, the eligibility criteria were that the respondent had to have provided sufficient information in the screening survey and have experience with online medical care or have been selected through random sampling described later if they had no experience with online medical care.

First, 40,000 individuals were selected from the Freeasy panel [9] to match the age and sex distribution of the Japanese population [10] following statistics published by the Japanese government (ie, the respondents were selected in order of earliest response [nonprobabilistic sampling], but the maximum number of respondents in each category was set based on the aforementioned distribution). They were asked to complete a screening questionnaire about their regular medical visits (currently visiting a medical facility on a regular basis; that is, more than once every 3 months) and experience with online medical care. Note that the survey panel used in this study did not include those who had difficulty accessing the internet as web-based operations were required for participation. The respondents to the screening survey were classified into the following four categories: (1) with regular visits and with online medical care experience, (2) with regular visits and without online medical care experience, (3) without regular visits and with online medical care experience, and (4) without regular visits and without online medical care experience. Second, the main survey was conducted on 3000 individuals with regular visits (ie, patients) and 3000 individuals without regular visits (ie, healthy individuals). As the number of individuals with online medical care experience was small in both groups, all the individuals were sampled. Alternatively, there were a large number of individuals who had no experience with online medical care; thus, stratified random sampling by age and sex was used to select target individuals in each group without regular visits (probabilistic sampling). All members of group 1 and part of group 2 were selected so that the total number of respondents in these groups was 3000. Similarly, all members of group 3 and part of group 4 were selected so that these groups would have a total of 3000 respondents. This approach ensured that each combined set of groups (groups 1 and 2 and groups 3 and 4) had a similar age and sex distribution. The participants were then asked to complete the main questionnaire. The survey request was sent out via the Freeasy system to the email addresses registered with Freeasy or posted on Freeasy’s affiliated website, and responses were completed on the web. The response period for the screening survey was September 25, 2023, to October 8, 2023, and that for the main survey was October 19, 2023, to November 2, 2023. The Checklist for Reporting Results of Internet E-Surveys [11] (Multimedia Appendix 5) provides further details on this survey.

### Survey of Medical Professionals

The eligibility criterion for this survey was that the respondent must be a physician, nurse, or administrative staff member of a medical institution covered by insurance in Japan as of June 2023.

Stratified random sampling by region and hospital category (probabilistic sampling) was used to select from 96,269 insured medical facilities in Japan as of June 2023. The regions were divided into 7 categories, facilities were divided into 2 categories (hospitals and clinics), and 350 facilities per category (350 × 2 × 7 = 4900 facilities in total) were selected. The sampling rate was 5% in total; however, it was set higher for the following facility categories that were anticipated to be underrepresented (ie, to include only a very small number of participants) through simple random sampling: specific functioning hospitals (sampling rate of 100%), including university hospitals and hospitals for advanced medical care; clinics for medical services in remote areas; and core hospitals for medical services in remote areas (sampling rates of 50%). The study descriptions and requests for survey responses were mailed to the hospital administrator of each facility, who was instructed to distribute survey completion requests to 3 respondents at their facility (1 physician, 1 nurse, and 1 member of the administrative staff). The respondents were asked to complete a web-based form using a shortened URL or QR code. In cases of duplicate responses, only the first response was considered valid. The response period was October 30, 2023, to December 8, 2023. The Checklist for Reporting Results of Internet E-Surveys (Multimedia Appendix 5) [11] provides further details on this survey.

### Data Analysis

The questionnaire responses were summarized for descriptive purposes and then compared between groups of respondents with or without online medical care experience and background factors. The chi-square and Mann-Whitney *U* tests were used for binary or categorical and ordinal variables, respectively, and the McNemar test was used when comparing two groups with correspondence. *P* values of <.05 were considered statistically significant. A multivariate logistic regression analysis was also performed of the outcome of experience or satisfaction with online medical care, with age, sex, number of nearby medical facilities (3-point Likert scale), and perceived effort of visiting the hospital (4-point Likert scale) included as covariates. All analyses were performed using Stata (version 17.0; StataCorp).

### Ethical Considerations

This entire study (including the interviews, the patient and healthy individual survey, and the medical professional survey) was approved by the Research Ethics Committee of the Faculty of Medicine at the University of Tokyo (2023024NI) and was conducted in accordance with the tenets of the Declaration of Helsinki. For the interviews, which were conducted to develop the subsequent questionnaires for patients and healthy individuals and medical professionals, written informed consent was obtained from all the participants, and the interview responses did not contain any personal information. In the questionnaire survey of patients and healthy individuals, the recruitment of patients was performed through the iBRIDGE company (Freeasy system), informed consent was obtained on the web at the time of each response on Freeasy-affiliated websites, and responses were provided by the company in a completely anonymized form. For each question answered, respondents received points worth ¥1 (US $0.007) as a reward (according to the survey company’s rules and regulations). Points can be exchanged for gift certificates or discount coupons through Freeasy-affiliated websites. In the questionnaire survey of medical professionals, a consent form was not required because the submission of responses was considered as consent to participate. To exclude duplicate responses, respondents were asked to provide only their facility name, title, and initials. In other words, no explicit personal information was included in the survey content, but potential identifiers were included. The responses to the medical professional survey, which included potential identifiers, were stored in a password-locked and encrypted hard disk in a secure room. No compensation was provided for the medical professional participants.

## Results

### Patient and Healthy Individual Characteristics

The proportion of women aged ˃70 years in the panel and the allocation to this group were slightly lower than those in the population distribution. In total, 92.5% (36,998/40,000) of individuals responded to the screening questionnaire and were classified into four categories: (1) regular visits and with online medical care experience (1478/36,998, 3.99%), (2) regular visits and without online medical care experience (16,269/36,998, 43.97%), (3) without regular visits and with online medical care experience (478/36,998, 1.29%), and (4) without regular visits and without online medical care experience (18,773/36,998, 50.74%). Among the respondents to the screening questionnaire, only 5.29% (1956/36,998) had online medical care experience. Second, all participants in group 1 (1478/1478, 100%) and some in group 2 (1522/16,269, 9.36%) were extracted (Figures S1A and S1B in Multimedia Appendix 6). Similarly, all participants in group 3 (478/478, 100%) and some in group 4 (2522/18,773, 13.43%) were extracted (Figures S1C and S1D in Multimedia Appendix 6). The main questionnaire was then administered to the selected individuals. The response rate in each group was >80% (1312/1478, 88.77%; 1281/1522, 84.17%; 404/478, 84.5%; and 2226/2522, 88.26% in groups 1, 2, 3, and 4, respectively). More than 80% of the respondents (4438/5223, 84.97%) were aged <60 years.

Regarding the number of nearby medical facilities, a major proportion of respondents with online medical care experience reported that there were many medical facilities nearby (Figure S2A in Multimedia Appendix 6). A greater percentage of respondents with online medical care experience reported that it took considerable effort to go to the hospital (Figure S2B in Multimedia Appendix 6). Among the respondents with regular visits, those with online medical care experience visited the clinic more frequently than those without (Figure S3A in Multimedia Appendix 6). One-way trips to the clinic were shorter for the group with no online medical care experience (Figure S3B in Multimedia Appendix 6).

### Characteristics of Medical Professionals

Of the 1580 responses, 1552 (98.23%) were valid after excluding duplicate responses and responses from out-of-scope facilities. When categorized by job, 39.82% (618/1552) of the responses were from physicians, 27.58% (428/1552) were from nurses, and 32.6% (506/1552) were from administrative staff members. When divided by online medical care practice, 78.87% (1224/1552) did not practice online medical care at their facility, 9.15% (142/1552) practiced online medical care at their facility but with no direct involvement, and the remaining 11.98% (186/1552) were directly involved in online medical care.

At the facility level, responses were received from 16.2% (794/4900) of the sampled facilities. The response rates from specific functioning hospitals, core hospital for medical services in remote areas, and clinic for medical services in remote areas were higher than those from other hospitals (specific functioning hospitals: 38/88, 43%; clinic for medical services in remote areas: 130/504, 25.8%; core hospitals for medical services in remote areas: 35/170, 20.6%; other hospitals: 297/2196, 13.5%; other clinics: 298/1946, 15.31%. Note that 4 facilities were categorized as specific functioning hospitals and at the same time as core hospitals for medical services in remote areas).

### Patients’ and Healthy Individuals’ Perspectives on Current Online Medical Care Use

In the group with online medical care experience, >50% of the patients selected internal medicine as the department in which they received online medical care (705/1312, 53.73% and 231/404, 57.2% of respondents with and without regular visits); followed by dermatology and psychiatry or psychosomatic medicine: 18.14% (238/1312), 16.6% (67/404), 18.06% (237/1312), 8.4% (34/404) for respondents with and without regular visits, respectively. In the group with no experience with online medical care, when asked to indicate which departments they would be willing to consider receiving online medical care in, internal medicine and psychiatry or psychosomatic medicine were the most common (587/1281, 45.82%; 786/2226, 35.31%; and 397/1281, 31%; 518/2226, 23.27% of respondents with and without regular visits, respectively; Table S1 in Multimedia Appendix 6).

Table 1 shows the results of the multivariate analysis of the online medical care experience with the variables of age of >60 years (binary variable), sex, number of nearby medical facilities (3-point Likert scale), and perceived effort of visiting the hospital (4-point Likert scale). The more nearby medical facilities, the more likely the respondent was to have experienced online medical care (adjusted odds ratio [aOR] 1.33, 95% CI 1.18-1.50 per point in the patient group; aOR 1.43, 95% CI 1.22-1.68 per point in the healthy individual group). The more effort they felt it was to visit the hospital, the more likely the respondent was to have experienced online medical care (aOR 1.48, 95% CI 1.35-1.63 per point in the patient group; aOR 1.57, 95% CI 1.38-1.78 per point in the healthy individual group).

**Table 1:** Multivariate analysis of factors related to experience and satisfaction with online medical care.

Factors	Experience, adjusted odds ratio (95% CI)		Satisfaction among respondents with experience, adjusted odds ratio (95% CI)	
	Patients (n=2593)	Healthy individuals (n=2630)	Patients (n=1312)	Healthy individuals (n=404)
Age of >60 y	1.09 (0.89-1.34)	1.07 (0.77-1.49)	1.04 (0.74-1.46)	0.54 (0.27-1.07)
Male sex	1.01 (0.86-1.18)	1.07 (0.86-1.32)	0.88 (0.67-1.16)	0.67 (0.40-1.13)
Medical facilities nearby^a^	*1.33* *(1.18-1.50)* ^b^	*1.43* *(1.22-1.68)*	*1.40* *(1.14-1.73)*	*1.77* *(1.23-2.54)*
Effort for hospital visits^c^	*1.48 (1.35-1.63)*	*1.57* *(1.38-1.78)*	*1.50* *(1.27-1.76)*	*1.77* *(1.31-2.40)*

^a^Ordinal variable (1=few, 2=reasonable, and 3=many).

^b^Italicization indicate statistical significance (the 95% CI of the adjusted odds ratio does not exceed 1).

^c^Ordinal variable (1=no, 2=negligible, 3=minimal, and 4=substantial).

The participants were asked to rate their level of satisfaction with online medical care on a 4-point scale (*very satisfied*, *generally satisfied*, *not very satisfied*, and *not satisfied at all*). Approximately 80% of the respondents in both groups selected the top 2 choices, “very satisfied” or “generally satisfied” (1023/1312, 77.97% and 322/404, 79.7% of respondents with and without regular visits, respectively; Table S2 in Multimedia Appendix 6). When stratified, respondents without regular visits aged ˂60 years or who had more medical facilities nearby were substantially more satisfied with online medical care (Table S2 in Multimedia Appendix 6). The groups that felt that going to the hospital required more work and that felt that online medical care was less burdensome than face-to-face medical care were more satisfied with online medical care (Table S2 in Multimedia Appendix 6).

As with the online medical care experience, a multivariate logistic analysis of satisfaction with online medical care (selecting the top 2 levels on a 4-point satisfaction scale was defined as being satisfied) showed that the greater the number of nearby medical facilities, the more satisfied the participants were (aOR 1.40, 95% CI 1.14-1.73 per point in the patient group; aOR 1.77, 95% CI 1.23-2.54 per point in the healthy individual group; Table 1). The more effort they felt it was to visit the hospital, the more satisfied they were (aOR 1.50, 95% CI 1.27-1.76 per point in the patient group; aOR 1.77, 95% CI 1.31-2.40 per point in the healthy individual group).

In the group with online medical care experience, the respondents answered that they wanted to use online medical care and searched for a medical facility that could provide it (870**/**1312, 66.31% and 217**/**404, 53.7% of the respondents with and without regular visits) rather than their physicians or medical facilities recommending the use of online medical care (442**/**1312, 33.69% and 152**/**404, 37.6% of those with and without regular visits). When the respondents without online medical care experience were asked about situations in which they would be willing to use online medical care, a relatively high number of respondents chose “when offered the option of online medical care by a physician or medical facility where they had already received face-to-face medical care” (696**/**1281, 54.33% and 932**/**2226, 41.87% of those with and without regular visits).

### Medical Professionals’ Perspectives on Current Online Medical Care Practice

Among the health care facilities that responded to the survey, the implementation rate of online medical care was 20.4% (162/794). Of the responding facilities, only 71.4% (567/794) reported using EMRs, and facilities that used EMR systems had significantly higher online medical care practice rates (142/567, 25% vs 20/227, 8.8%; *P*<.001). The implementation rate of online medical care did not differ between hospitals and clinics (75/366, 20.5% for hospitals and 87/428, 20.3% for clinics); however, among hospitals, the rate was higher for specific functioning hospitals than for core hospital for medical services in remote areas or other hospitals (16/38, 42.1% for specific functioning hospitals; 7/35, 20% for core hospital for medical services in remote areas; and 53/297, 17.8% for other hospitals).

The most frequently selected scenarios in which online medical care was provided were prescriptions of the same medication as usual (106/186, 57%), routine medical checkups (98/186, 52.7%), and difficulty attending hospital consultations owing to transportation issues and living in a remote area (68/186, 36.6%; Table 2). The most common problem with the system when implementing online medical care was that it could not be implemented on the same computer as the EMR system (Table 2).

**Table 2:** Online medical care situations and problems with its system as answered by health care professionals who are directly involved in online medical care (results of the medical professional survey; n=186).

Situations and problems		Participants, n (%)
**Situations in which online medical care is provided**		
	Prescription of the same medication as usual	106 (57)
	Routine medical checkups	98 (52.7)
	Difficulty attending hospital consultations (eg, transportation issues and living in a remote area)	68 (36.6)
	Outpatient visits for fever and other situations in which some type of infectious disease is anticipated	62 (33.3)
	Explanation of test results	61 (32.8)
	Visit for sudden onset of illness (acute illness) other than infectious diseases	19 (10.2)
	Second opinion	13 (7)
	Consultations when there is no specialist physician nearby	12 (6.5)
	Consultation on whether to see a physician	12 (6.5)
	Nutritional guidance	7 (3.8)
	Consultation services related to childcare (eg, developmental counseling and childcare support)	4 (2.2)
	Conferences for home medical care	4 (2.2)
	Details not known by the respondents	3 (1.6)
	Perinatal counseling services (eg, motherhood classes and genetic, infertility, and pregnancy complication counseling)	2 (1.1)
	Other	12 (6.5)
**Problems identified with the online medical care system**		
	Cannot be implemented on the same terminal as the electronic medical record system	95 (51.1)
	Inability to screen share documents such as test results (must be captured on a camera)	82 (44.1)
	Inability to draw illustrations on the screen (must be written on a piece of paper by hand and captured on a camera)	72 (38.7)
	Communication environment problems causing issues	64 (34.4)
	Difficulty operating on the patient side	59 (31.7)
	Difficulty operating on the medical institution side	19 (10.2)
	Can be performed on the same terminal as the electronic medical record system, but the software is not integrated and is difficult to use	13 (7)
	None that apply	26 (14)

Most respondents (128/186, 68.8%) indicated that the hospital tasks required for online medical care were “obviously/somewhat more complicated” than those for face-to-face medical care (Table S3 in Multimedia Appendix 6). A major proportion of respondents among the administrative staff (33/43, 77%) chose “obviously/somewhat more complicated,” and none chose “obviously easier.”

Only 14% (26/186) of the respondents reported that they could see more patients per unit time with online medical care than with face-to-face medical care, whereas 44.1% (82/186) reported that they could see fewer patients with online medical care (Table S3 in Multimedia Appendix 6).

### Patient Burdens Associated With Medical Visits

Both patients and healthy individuals and medical professionals were asked how the patient burden associated with medical visits differed between online medical care and face-to-face medical care. Regarding time and physical burden, most respondents indicated that online medical care was less burdensome, with comparable results for patients and healthy individuals and medical professionals (1352/2226, 60.74% to 307/404, 76% and 1285/2226, 57.73% to 116/142, 81.7%, respectively, depending on the respondent category; Table 3). Most patients and healthy individuals with online medical care experience indicated that online medical care reduces mental and financial burden (mental burden: 735/1312, 56.02% and 252/404, 62.4% for those with and without regular visits, respectively; financial burden: 671/1312, 51.14% and 172/404, 42.6% for those with and without regular visits, respectively).

**Table 3:** Percentage of respondents reporting that online medical care (OMC) is less burdensome than face-to-face medical care (results of the patient and healthy individual and medical professional surveys)^a^.

Respondents				Time burden, n (%)		Physical burden, n (%)		Mental burden, n (%)		Financial burden, n (%)
**Patients and healthy individuals**										
	**With regular visits** **(OMC experience)**									
		Experienced (n=1312)	986 (75.15)		891 (67.91)		735 (56.02)		671 (51.14)	
		Not experienced (n=1281)	929 (72.52)		859 (67.06)		502 (39.19)		463 (36.14)	
	**Without regular visits** **(OMC experience)**									
		Experienced (n=404)	307 (75.99)		310 (76.73)		252 (62.38)		172 (42.57)	
		Not experienced (n=2226)	1352 (60.74)		1285 (57.73)		792 (35.58)		753 (33.83)	
**Medical personnel** **(OMC practice)**										
	Not provided (n=1224)			897 (73.28)		933 (76.23)		438 (35.78)		549 (44.85)
	Provided but not involved^b^ (n=142)			103 (72.54)		116 (81.69)		70 (49.3)		65 (45.77)
	Involved (n=186)			137 (73.66)		140 (75.27)		84 (45.16)		77 (41.4)

^a^In this survey, the financial burden included medical fees, transportation costs, line charges, and various other expenses related to medical visits.

^b^The facility provides OMC practice but the respondent himself or herself is not involved in it.

The acceptability of medical visits was also surveyed according to the frequency and modality of medical visits. The acceptability rate did not differ significantly between face-to-face medical care and online medical care in patients with online medical care experience (Table 4). When stratified by age, those aged ≥60 years with no online medical care experience tended to prefer face-to-face medical care over online medical care regardless of the frequency of medical visits.

**Table 4:** Acceptability of online medical care (OMC) and face-to-face medical care (FMC) in relation to the frequency of visits (results of the patient and healthy individual survey).

			Once a week							Once every 2 weeks							Once a month				
Patients and healthy individuals			FMC, n (%)		OMC, n (%)		*P* value		FMC, n (%)			OMC, n (%)		*P* value		FMC, n (%)			OMC, n (%)		*P* value
**With** **regular visits (OMC experience)**																					
	Experienced: all (n=1312)	797 (60.74)		833 (63.49)		.09		849 (64.71)			886 (67.53)		.07		1037 (79.04)			1028 (78.35)		.62	
	Experienced: aged <60 y (n=1074)	672 (62.57)		696 (64.8)		.21		702 (65.36)			741 (69)		*.03* ^a^		832 (77.47)			845 (78.68)		.43	
	Experienced: aged ≥60 y (n=238)	125 (52.52)		137 (57.56)		.17		147 (61.76)			145 (60.92)		.81		205 (86.13)			183 (76.89)		*.0* *05*	
	Not experienced: all (n=1281)	529 (41.3)		558 (43.56)		.17		663 (51.76)			617 (48.17)		*.03*		1075 (83.92)			778 (60.73)		*<.001*	
	Not experienced: aged <60 y (n=1039)	426 (41)		463 (44.56)		*.05*		536 (51.59)			515 (49.57)		.25		862 (82.96)			644 (61.98)		*<.001*	
	Not experienced: aged ≥60 y (n=242)	103 (42.56)		95 (39.26)		.41		127 (52.48)			102 (42.15)		*.01*		213 (88.02)			134 (55.37)		*<.001*	
**Without** **regular visits (OMC experience)**																					
	Experienced: all (n=404)	205 (50.74)		258 (63.86)		*<.001*		246 (60.89)			280 (69.31)		*.001*		319 (78.96)			320 (79.21)		.90	
	Experienced: aged <60 y (n=354)	183 (51.69)		231 (65.25)		*<.001*		218 (61.58)			251 (70.9)		*.001*		279 (78.81)			286 (80.79)		.35	
	Experienced: aged ≥60 y (n=50)	22 (44)		27 (54)		.20		28 (56)			29 (58)		.78		40 (80)			34 (68)		.08	
	Not experienced: all (n=2226)	902 (40.52)		912 (40.97)		.69		1114 (50.04)			996 (44.74)		*<.001*		1531 (68.78)			1198 (53.82)		*<.001*	
	Not experienced: aged <60 y (n=1971)	763 (38.71)		796 (40.39)		.15		950 (48.2)			874 (44.34)		*.001*		1318 (66.87)			1063 (53.93)		*<.001*	
	Not experienced: aged ≥60 y (n=255)	139 (54.51)		116 (45.49)		*.02*		164 (64.31)			122 (47.84)		*<.001*		213 (83.53)			135 (52.94)		*<.001*	

^a^Italicization indicates statistical significance.

### Comparison of Face-to-Face Medical Care and Online Medical Care for Various Medical Procedures

The overwhelming majority of medical professionals answered that general impressions and physical examination findings were easier (chose “somewhat easier” or “clearly easier”) to obtain in face-to-face medical care than in online medical care (1467/1552, 94.52% and 1500/1552, 96.65%, respectively; Table 5). Most respondents also answered that verbal communication, interviews with family members, and building trust between patients and physicians were easier in face-to-face medical care (1059/1552, 68.23%; 772/1552, 49.74%; and 1207/1552, 77.77%, respectively). In contrast, a higher proportion of respondents indicated that visual understanding of the patient’s medical condition and behavior at home and visual understanding of the home and other environments were relatively easier in online medical care (642/1552, 41.37% and 1057/1552, 68.11%, respectively).

**Table 5:** Ease of performing various medical procedures (results of the medical personnel survey).

Procedures	Clearly easier in OMC^a^, n (%)	Somewhat easier in OMC, n (%)	Almost the same, n (%)	Somewhat easier in FMC^b^, n (%)	Clearly easier in FMC, n (%)
General impression (n=1552)	7 (0.45)	15 (0.97)	63 (4.06)	364 (23.45)	1103 (71.07)
Physical examination findings (n=1552)	8 (0.52)	7 (0.45)	37 (2.38)	310 (19.97)	1190 (76.68)
Verbal communication (n=1552)	7 (0.45)	33 (2.13)	453 (29.19)	476 (30.67)	583 (37.56)
Interviews with family members (n=1552)	35 (2.26)	174 (11.21)	571 (36.79)	348 (22.42)	424 (27.32)
Visual understanding of the patient’s medical condition and behavior at home (n=1552)	181 (11.66)	461 (29.7)	297 (19.14)	241 (15.53)	372 (23.97)
Visual understanding of the home and other environments (n=1552)	507 (32.67)	550 (35.44)	222 (14.3)	108 (6.96)	165 (10.63)
Building trust between patients and physicians (n=1552)	2 (0.13)	14 (0.9)	329 (21.2)	519 (33.44)	688 (44.33)

^a^OMC: online medical care.

^b^FMC: face-to-face medical care.

### Patients Appropriate for Online Medical Care

When asked what percentage of patients attending their hospital would benefit from online medical care, 72.62% (1127/1552) of medical professionals answered “10%” to “30%,” with “10%” being the most common answer (636/1552, 40.98%; Figure S4A in Multimedia Appendix 6). A few respondents (203/1552, 13.08%) answered “0%.” These results showed no significant difference in opinions between respondents who provided online medical care and those who did not (Figure S4B in Multimedia Appendix 6).

Regarding the maximum age at which a patient can receive online medical care alone, the most common response from medical professionals was “60 years old” (369/1552, 23.78%), and a similar proportion of respondents (344/1552, 22.16%) answered that age was not a relevant factor (Figure S4C in Multimedia Appendix 6). No significant differences were observed in the distribution of responses between respondents who provided online medical care or not (Figure S4D in Multimedia Appendix 6).

### Factors Limiting Online Medical Care Expansion

In the survey of patients and healthy individuals, 16 options were presented as factors preventing the expansion or adoption of online medical care, and each respondent was allowed to select 3 options. The most selected factor was “online medical care requires patients to visit a hospital when tests or procedures are needed.” In addition, “concerns about the adequacy of the clinical examination conducted during online medical care,” “despite being aware of online medical care, there is uncertainty or lack of clarity regarding when online medical care is appropriate or desired,” and “face-to-face communication is easier than web-bsed interactions” were commonly chosen (Table 6). The top 3 choices slightly differed depending on whether the patient had regular visits and whether the patient had previous experience with online medical care.

**Table 6:** Factors hindering online medical care (OMC) adoption and expansion.

Factors	Patients and healthy individuals, n (%)					Medical professionals, n (%)			
	Without regular visits and with OMC experience (n=404)	Without regular visits and without OMC experience (n=2226)	With regular visits and with OMC experience (n=1312)	With regular visits and without OMC experience (n=1281)	All (n=1552)		OMC practice		
							Not provided (n=1224)	Provided but not involved^a^ (n=142)	Involved (n=186)
OMC increases administrative procedures on the part of medical institutions	43 (10.64)	250 (11.23)	195 (14.86)	131 (10.2)	598 (38.53)		466 (38.07)	51 (35.92)	*81 (43.55)* ^b^
OMC requires more time and effort on the part of physicians	—^c^	—	—	—	397 (25.58)		322 (26.31)	29 (20.42)	46 (24.73)
OMC requires more time and effort on the part of the patient	40 (9.9)	269 (12.08)	181 (13.8)	132 (10.3)	380 (24.48)		303 (24.75)	29 (20.42)	48 (25.81)
OMC places a heavy financial burden on the medical institution	37 (9.16)	191 (8.58)	191 (14.56)	81 (6.3)	489 (31.51)		404 (33.01)	31 (21.83)	54 (29.03)
OMC imposes a heavy financial burden on patients	47 (11.63)	221 (9.93)	148 (11.28)	116 (9.1)	140 (9.02)		104 (8.5)	16 (11.27)	20 (10.75)
Difficult for the medical institution to construct a system and communication environment for OMC	62 (15.35)	307 (13.79)	215 (16.39)	172 (13.4)	*655 (42.2)*		*561 (45.83)*	36 (25.35)	58 (31.18)
Difficult for patients to download applications and build a communication environment for OMC	94 (23.27)	515 (23.14)	303 (23.09)	284 (22.2)	*880 (56.7)*		*701 (57.27)*	*82 (57.75)*	*97 (52.15)*
Concerns regarding the adequacy of the clinical examination during OMC	*128 (31.68)*	*705 (31.67)*	298 (22.71)	*420 (32.8)*	565 (36.4)		476 (38.89)	34 (23.94)	55 (29.57)
Face-to-face communication is easier than web-based interaction	112 (27.72)	592 (26.59)	273 (20.81)	*395 (30.84)*	620 (39.95)		507 (41.42)	48 (33.8)	65 (34.95)
OMC requires patients to visit a hospital to undergo tests or procedures	*126 (31.19)*	*718 (32.26)*	*400 (30.49)*	*509 (39.73)*	*906 (58.38)*		*736 (60.13)*	*80 (56.34)*	*90 (48.39)*
OMC may not be feasible for a larger proportion of patients	59 (14.6)	257 (11.55)	157 (11.97)	132 (10.3)	585 (37.69)		489 (39.95)	*53 (37.32)*	43 (23.12)
Concerns regarding breach of personal information through OMC	50 (12.38)	293 (13.16)	187 (14.25)	153 (11.94)	375 (24.16)		323 (26.39)	26 (18.31)	26 (13.98)
FMC^d^ is preferable to OMC for the education of medical students and young physicians	—	—	—	—	249 (16.04)		207 (16.91)	22 (15.49)	20 (10.75)
Lack of awareness regarding OMC	79 (19.55)	440 (19.77)	262 (19.97)	222 (17.33)	341 (21.97)		260 (21.24)	38 (26.76)	43 (23.12)
Despite awareness, there is a lack of clarity on when OMC is appropriate or desired	*114 (28.22)*	*610 (27.4)*	*354 (26.98)*	357 (27.87)	386 (24.87)		305 (24.92)	35 (24.65)	46 (24.73)
Lack of information on OMC providers	94 (23.27)	581 (26.1)	*326 (24.85)*	290 (22.64)	326 (21.01)		255 (20.83)	38 (26.76)	33 (17.74)
The level of satisfaction with FMC is high and many people do not need OMC	71 (17.57)	371 (16.67)	233 (17.76)	227 (17.72)	501 (32.28)		401 (32.76)	43 (30.28)	57 (30.65)
Regardless of the level of satisfaction with FMC, people tend to maintain the status quo	56 (13.86)	358 (16.08)	213 (16.23)	222 (17.33)	321 (20.68)		249 (20.34)	26 (18.31)	46 (24.73)

^a^The facility provides OMC practice but the respondent himself or herself is not involved in it.

^b^Italicization indicates the top 3 in each category.

^c^These items were not included as options in the patient and healthy individual survey.

^d^FMC: face-to-face medical care.

In the survey of medical professionals, 18 options were presented, and each respondent was allowed to select all of those that applied. When categorized by job type and practice status of online medical care, the top 2 choices for all groups were “online medical care requires patients to visit hospitals for mandatory tests and procedures” and “difficult for patients to download applications and build a communication environment for online medical care” (Table 6). When stratified by facility type, the results suggest that the complexity of administrative procedures was a disincentive for specific functioning hospitals, and 54% (43/80) of respondents selected “online medical care increases administrative procedures on the part of medical institutions” (Table S4 in Multimedia Appendix 6).

## Discussion

### Principal Findings

By conducting 2 nationwide surveys—one for patients and healthy individuals and the other for medical professionals—we acquired a comprehensive understanding of the current status of online medical care in Japan and identified critical concerns that must be addressed to facilitate its further adoption and expansion in the future. Although only 5.29% (1956/36,998) of the respondents had received online medical care and only 20.4% (162/794) of the institutions offered online medical care, satisfaction was high among approximately 80% (1023/1312, 77.97% and 322/404, 79.7%) of the patients and healthy persons who had received online medical care. If they had a large number of nearby medical facilities and felt that a lot of work was associated with medical visits, they had significantly more experience with online medical care and were more satisfied with it. The survey of medical professionals found that they felt that various medical procedures were more difficult to perform in online medical care compared to face-to-face medical care, whereas online medical care was perceived to reduce various patient burdens compared to face-to-face medical care. The number 1 factor currently preventing the adoption of online medical care in Japan, selected the most in both the patient and medical professional surveys, was that patients who receive online medical care are forced to switch to face-to-face medical care when tests or procedures become necessary. In addition, the lack of sufficient awareness of and education on online medical care both among patients and healthy individuals and among medical professionals may present challenges in performing certain medical procedures compared to face-to-face medical care.

In addition, Japan’s unique background needs to be mentioned. Japan has a large number of medical institutions, ranking third after Colombia and South Korea in the number of medical institutions per population in 2020 according to an Organisation for Economic Co-operation and Development report [12]. The fact that medical institutions are typically located nearby and face-to-face medical care is relatively readily available may impact online medical care diffusion. In addition, laboratory centers that perform blood and urine tests and x-rays for online medical care are only experimental and not widely used in Japan.

### Current Status of Online Medical Care in Japan

In the screening survey of approximately 40,000 patients and healthy individuals, only 5.29% (1956/36,998) of respondents had online medical care experience. Almost all the participants in the panel had smartphones, PCs, or other terminals and were presumed to have a high affinity for telemedicine as this respondent group had to respond via the internet; therefore, the actual experience rate of online medical care in Japan may be much lower.

In the survey of medical professionals, only 20.4% (162/794) of the facilities that responded had implemented online medical care. This result is similar to or slightly higher than the 15.6% telemedicine implementation rate in Japanese clinics in 2022 [13]. The implementation rate did not differ between hospitals and clinics; however, it was particularly high among specific functioning hospitals compared with other hospitals. This may be because specific functioning hospitals are involved in the treatment of or second opinions on rare or intractable diseases.

Although basic tools for telemedicine are available in Japan, many facilities are still operating in a traditional paper-based manner, with 28.6% (227/794) of the responding facilities not using EMRs. In the United States, data show that 88.2% of office-based physicians use EMRs [14]. In Japan, the prevalence of EMR use is 91.2% for large hospitals with ≥400 beds, which is almost the same as that in the United States. However, it is lower for general hospitals and general clinics at 57.2% and 49.9%, respectively [15]. The implementation rate of online medical care is high among facilities using EMR systems, suggesting that further dissemination of these systems may be necessary before online medical care becomes widespread.

Online medical care is most frequently used in internal medicine and dermatology. In addition to these departments, patients and healthy individuals with no online medical care experience preferred online medical care implementation in psychiatry and psychosomatic medicine. Although obtaining the opinions of psychiatry and psychosomatic medicine specialists regarding the feasibility of online medical care is necessary, many patients and healthy individuals are demanding online medical care in these departments. Consequently, telepsychiatry has become widespread in many countries, and in 15 of 17 regions in the world, telepsychiatry was reimbursed at the same rate as or a higher rate than in-person consultations during the COVID-19 pandemic [7].

Facilities engaging in medical services in remote areas, such as core hospital for medical services in remote areas and clinic for medical services in remote areas, also had a higher implementation rate of online medical care than others, which reflects the attempt to overcome geographical disadvantages through online medical care. In fact, in the survey of medical professionals, “difficulty in attending hospital consultations owing to transportation issues” and “living in a remote area” were often selected as situations in which online medical care was provided. However, in the survey of patients and healthy individuals, respondents with fewer nearby medical facilities expressed lower satisfaction with online medical care than those with more nearby medical facilities, although the reason for this contrasting finding was not clear. In previous reports, it has been noted that access to telemedicine in rural areas remains an important issue [16,17].

In the survey of patients and healthy individuals, satisfaction with online medical care was high. Online medical care was more satisfactory to patients and healthy individuals, especially those who felt that hospital visits required considerable effort and those who felt that online medical care reduced the burden of medical visits (Table 1). Furthermore, despite the availability of medical facilities nearby, those with online medical care experience perceived that online medical care reduced the efforts required to physically visit a hospital. The efforts required to make a hospital visit were determined by several other factors (eg, childcare, caregiving, work, busyness, and physical mobility difficulties) rather than distance from a medical facility. This suggests that online medical care may become even more widespread not only in underpopulated areas where online medical services are considered necessary but also in urban areas where there are many medical facilities. There is also a report from the United States that telemedicine has become more prevalent in urban areas than in rural areas after the COVID-19 pandemic [16].

Our survey revealed that medical professionals also recognize that online medical care reduces the burden on patients, improving the access of medical visits. In a survey of 31 countries conducted by the Organisation for Economic Co-operation and Development on the status of telemedicine implementation and other issues, national experts agreed that telemedicine services can positively impact several aspects of health system performance (ie, equity, efficiency, access, cost-effectiveness, effectiveness, safety, and quality) [17]. Online medical care is an important means of connecting patients who have interrupted their visits due to the burden of hospital visits but who still require medical care. However, this is only the case if they are able to use information and communications technology. In promoting the spread of online medical care, it is important to consider those who are not skilled in information and communications technology or else the so-called digital divide may be exacerbated.

In contrast, medical professionals implied that online medical care may not be as convenient to implement as face-to-face medical care as it is more difficult to perform various medical procedures necessary for diagnosis and determination of severity and there are still some problems with the system for performing online medical care.

Our survey results indicate that, while online medical care offers overwhelming advantages in terms of patient convenience and access to medical care, it is difficult for health care providers to offer a variety of medical services and set up a system for online medical care. As the magnitude of benefits for patients seems to outweigh the concerns on the part of health care providers, it seems that online medical care ought to be more widespread.

### Factors Limiting Online Medical Care Expansion and Possible Solutions

The major limiting factor for online medical care adoption was that “online medical care requires patients to make a hospital visit when tests or procedures are needed.” The high selection rate for this option indicates that the lack of access to tests and physical examinations in online medical care is a drawback. This is consistent with the fact that many health care providers responded that performing various medical procedures was more difficult in online medical care than in face-to-face medical care (Table 5). To overcome this, it is necessary to develop separate access to tests or procedures specifically tailored for online medical care or alternative means of obtaining this diagnostic information. In particular, patients desire technologies or tools that make it easier to obtain information for accurate condition assessment, such as video calls with higher resolution and 3D effects, as well as alternative methods for assessing physical findings. Furthermore, for some types of telemedicine, it may be beneficial to have a nurse by the patient's side while communicating with the physician online. The nurse’s presence on the patient’s side eliminates operational concerns and problems and provides information that is difficult to obtain in online medical care, such as physical findings. A previous systematic review summarizing the barriers to adapting to telemedicine reported that the top barriers included technically challenged staff, resistance to change, cost, reimbursement, and patient age and educational level; however, it did not address the need to visit the hospital for physical examinations and tests [18]. In a previous questionnaire survey with medical professionals and another interview survey with patients, concerns related to the lack of physical examinations were cited as barriers to the use of online medical care [19,20]. Such factors can be identified through questionnaires and interviews.

The following responses were also common: “difficult for patients to download applications and build a communication environment for online medical care,” “despite being aware, people do not know when online medical care is appropriate or desired,” and “concerns regarding the adequacy of the clinical examination in online medical care.” Thus, applications for online medical care that can be easily understood from the patients’ perspective should be developed. In the free-response section of the questionnaire, some respondents suggested that booths for online medical care should be set up at city halls, convenience stores, and other public places and that a unified standardization of applications is needed to avoid confusion (data not shown). In addition, offering educational activities regarding online medical care and releasing a list of facilities that implement online medical care in an easy-to-read format for the general public should be considered. Attitudes toward online medical care may change after individuals gain firsthand experience with it.

Promoting telemedicine among patients who are suitable for online medical care or patients who have a disease or condition that is suitable for online medical care could be a realistic strategy. For example, medical professionals answered that it seems to be easier to “visually grasp the patient at home” and “visually grasp the environment of the patient’s daily life at home” with online medical care than with face-to-face medical care (Table 5). The conditions in which these medical procedures are highly important include caring for children and older patients whose usual condition is difficult to understand during hospital visits. The fact that those aged ˂60 years exhibited higher satisfaction rates with online medical care than those aged ˃60 years does not preclude its use by older adults; however, it may be a good idea to appeal to younger people, including those caring for older individuals. Health care providers who are younger are more likely to adopt telemedicine [21]. Given that many respondents expressed their willingness to switch to online medical care if offered by their trusted physicians and medical institutions that currently provide face-to-face medical care, approaches from the medical provider side should be considered for further promotion of online medical care. Notably, patients prefer continuity of care and receiving online medical care from a physician they already know to receiving face-to-face medical care from unfamiliar providers [22].

### Limitations

Despite these advantages, our study has certain limitations that should be acknowledged. First, because this was a voluntary survey, there may be a selection bias in that those who are satisfied with or interested in online medical care may respond more positively to the survey. Furthermore, it is estimated that the participation of those with low digital literacy was low due to the web-based nature of the survey. Second, compared to the high response rate for the patient and healthy individual survey, the overall response rate for the medical professionals’ survey was low (794/4900, 16.2%), and it is possible that the results do not reflect the whole picture because of nonresponse bias. Specifically, it is possible that responses were not received from medical professionals who were too busy to respond. In addition, although our survey did not allow us to investigate the reasons for nonresponse, if those opposed to online medical care tended to be nonrespondents, the results of this survey could be skewed from the overall trend. Third, although part of our results can be extrapolated to other countries, they reflect the actual situation in Japan as of 2023. Furthermore, because this was a questionnaire survey, recall bias and erroneous answers may have been included in the results.

### Conclusions

In conclusion, our nationwide surveys revealed the current status of online medical care in Japan and simultaneously identified several problems and issues related to online medical care, which will be useful in considering its expansion or adoption. On the basis of the identified factors preventing the spread of online medical care, it is important to develop technology and improve the system of medical care so that tests and procedures can be performed seamlessly with online medica care, as well as informing and educating the general population, including patients and the medical community.

## Appendix

Multimedia Appendix 1 Japanese questionnaire for patients and healthy individuals.

Multimedia Appendix 2 English translated questionnaire for patients and healthy individuals.

Multimedia Appendix 3 Japanese questionnaire for medical professionals.

Multimedia Appendix 4 English translated questionnaire for medical professionals.

Multimedia Appendix 5 Checklist for Reporting Results of Internet E-Surveys.

Multimedia Appendix 6 Tables S1-S4 and Figures S1-S4.

## Abbreviations

aOR: adjusted odds ratio
EMR: electronic medical record

## Data Availability

The datasets generated during and analyzed during this study are available from the corresponding author on reasonable request.
