# Correction: Issues in the Adoption of Online Medical Care: Cross-Sectional Questionnaire Survey

**DOI:** 10.2196/68439

**Published:** 2024-11-27

**Authors:** Yuka Sugawara, Yosuke Hirakawa, Masao Iwagami, Haruo Kuroki, Shuhei Mitani, Ataru Inagaki, Hiroki Ohashi, Mitsuru Kubota, Soichi Koike, Rie Wakimizu, Masaomi Nangaku

**Affiliations:** 1 Division of Nephrology and Endocrinology The University of Tokyo Tokyo Japan; 2 Department of Health Services Research, Institute of Medicine University of Tsukuba Ibaraki Japan; 3 Clinic Pauroom Tokyo Japan; 4 Department of Education, College of Education, Psychology and Human Studies Aoyama Gakuin University Tokyo Japan; 5 TAMA Family Clinic Tokyo Japan; 6 Department of General Pediatrics and Interdisciplinary Medicine National Center for Child Health and Development Tokyo Japan; 7 Division of Health Policy and Management, Center for Community Medicine Jichi Medical University Tochigi Japan; 8 Department of Child Health and Development Nursing, Institute of Medicine University of Tsukuba Ibaraki Japan

In “Issues in the Adoption of Online Medical Care: Cross-Sectional Questionnaire Survey” ([J Med Internet Res 2024;1(26):e64159]) the authors made on correction.

In the originally published manuscript, the following sentence:

“Consequently, telepsychiatry has become widespread in many countries, and in 15 of 17 regions in Japan,…”

Was corrected as follows:

“Consequently, telepsychiatry has become widespread in many countries, and in 15 of 17 regions in the world,…”.

The correction will appear in the online version of the paper on the JMIR Publications website on November 27, 2024, together with the publication of this correction notice. Because this was made after submission to PubMed, PubMed Central, and other full-text repositories, the corrected article has also been resubmitted to those repositories.

