# Supplementary material for: Issues in the Adoption of Online Medical Care: Cross-Sectional Questionnaire Survey
Source: J Med Internet Res. 2024 Nov 1;26:e64159. doi: 10.2196/64159 (PMC11568393; doi:10.2196/64159)
Supplement: Multimedia Appendix 1 [file jmir_v26i1e64159_app1.doc]

**Appendix 1. Original (Japanese) questionnaire for patients and healthy individuals.**

Screening questionnaire -----------------------------------------------------------p.2

Main questionnaire (1)-------------------------------------------------------------p.4

Main questionnaire (2)-------------------------------------------------------------p.10

Main questionnaire (3)-------------------------------------------------------------p.16

Main questionnaire (4)-------------------------------------------------------------p.21

**Screening questionnaire**

1.

以下のそれぞれにつき、当てはまるものにチェックを入れてください。なお、定期受診とは少なくとも３か 月に１回の頻度での受診をさします。（いくつでも）*回答必須

□ご自身がどこかの医療機関に定期受診している

□小学校入学前のお子さんの医療機関への定期受診に付き添っている

□小学生～中学生のお子さんの医療機関への定期受診に付き添っている

□ご家族・同居人など15歳以上の方の医療機関への定期受受診に付き添っている

□あてはまるものはない

2.

あなたは、オンライン診療（“医師－患者間において、情報通信機器を通して、患者の診察及び診断を行い 診断結果の伝達や処方等の診療行為を、リアルタイムにより行う行為”を指し、電話診療は含まれません）を一度でも利用したことがありますか。（ひとつだけ）

*回答必須

○利用したことがある

○利用したことはない

分岐条件-Q1でいずれかを選択した選択肢： [2.小学校入学前のお子さんの医療機関への定期受診に付き添 っている～4.ご家族・同居人など15歳以上の方の医療機関への定期受診に付き添っている]

3. あなたが付き添っているご家族は、オンライン診療（“医師－患者間において、情報通信機器を通して、患 者の診察及び診断を行い診断結果の伝達や処方等の診療行為を、リアルタイムにより行う行為”を指指し、電 話診療は含まれません）を一度でも利用したことがありますか。（ひとつだけ）*回答必須

○利用したことがある

○利用したことはない

分岐条件 - Q1でいずれかを選択した選択肢： [1.ご自身がどこかの医療機関に定期受診している]

4.

あなたの現在の定期通院先は以下のどれにあたりますか。（いくつでも）*回答必須

□診療所・クリニック

□一般病院

□大学病院やセンター病院などの大きな病院（特定機能病院・地域医療支援病院など）

□現在通院中ではない

分岐条件 - Q1でいずれかを選択した選択肢： [2.小学校入学前のお子さんの医療機関への定期受診に付き添 っている～4.ご家族・同居人など15歳以上の方の医療機関への定期受診に付き添っている]

排他 ”現在通院中ではない” を選択した場合は他の項目、選択肢を答えることができません

5.

あなたが付き添われているご家族の現在の定期通院先は以下のどれにあたりますか。複数のご家族に付き添 われている場合には、どなたか任意のお一人についてご回答ください。（いくつでも）*回答必須

□診療所・クリニック

□一般病院

□大学病院やセンター病院などの大きな病院（特定機能病院・地域医療支援病院など）

□現在通院中ではない

**Main questionnaire (1)**

1.

事前調査で、

**・あなた⾃⾝あるいはご家族は、定期通院中である**

**・あなた⾃⾝あるいはご家族は、オンライン診療の経験がある**

と回答された⽅向けの設問です。当てはまっていない場合には、現時点で回答を中断してください。

【ご回答前の注意事項｜必ずお読みください】 ・この調査の回答にあたっては、定期通院されているご⾃⾝、あるいはあなたが付き添って定期受診するご家族のうち、どなたか任意のお⼀⼈を想定し、ご回答ください。 上記の注意事項をお読みいただいた上で、どなたについてご回答されますか。（ひとつだけ）*回答必須

○あなた⾃⾝について回答する

○あなたが受診に付き添っている⽅について回答する

分岐条件 数値のみ Q1でいずれかを選択した選択肢 [2.あなたが受診に付き添っている⽅について回答する]

2.

あなたが付き添っている、定期受診されている⽅は何歳ですか︖整数で記載してください。*回答必須

定期受診されている⽅の年齢 *　歳

3.

現在の受診頻度として、最もあてはまるものを選択してください。（ひとつだけ）*回答必須 ○週1回程度

○2週に1回程度

○⽉1回程度

○2ヶ⽉に1回程度

○3か⽉に1回程度

4.

現在通院している医療機関への⽚道の所要時間として、最もあてはまるものを選択してください。（ひとつだけ）*回答必須

【最初の質問で「あなたが受診に付き添っている⽅について回答する」を選択した⽅へ】 あなた（回答者）は定期受診に付き添っているが、定期受診されている⽅とは居住地が異なる場合、あなた（回答者）ではなく定期受診されている⽅の所⽤時間を ご回答ください。

○30分未満

○30分以上1時間未満

○１時間以上２時間未満

○２時間以上

5.

お住まいの地域には、どのくらい多くの医療機関がありますか。あなたの印象を教えてください。（ひとつだけ）*回答必須

【最初の質問で「あなたが受診に付き添っている⽅について回答する」を選択した⽅へ】 あなた（回答者）は定期受診に付き添っているが、定期受診されている⽅とは居住地が異なる場合、あなた（回答者）ではなく定期受診されている⽅のお住まいの 地域についてご回答ください。

○近くに医療機関が数多く存在する

○近くに医療機関がそれなりの数存在する

○近くに医療機関はあまり存在しない

6.

通院における⼿間（移動の⼤変さ、所要時間、仕事の調整、⼦供の世話の調整等）について、あなた（回答者）はどのように感じますか。 （ひとつだけ）*回答必須

○かなり⼿間がかかる

○やや⼿間がかかる

○あまり⼿間はかからない

○全く⼿間はかからない

7.

以下の項⽬のそれぞれについて、選択肢から最も当てはまるものを⼀つ選択してください。*回答必須

【最初の質問で「あなたが受診に付き添っている⽅について回答する」を選択した⽅へ】

あなたが定期受診へ付き添う場合を想定して、ご回答ください。

|  | 全く問題なく 許容可能である | 抵抗はあるが 許容可能である | できれば 避けたい | 絶対に 許容できない |
| --- | --- | --- | --- | --- |
| 対面診療における |  |  |  |  |
| **週1回**程度の受診 |
| 対面診療における |  |  |  |  |
| **2週に1回**程度の受診 |
| 対面診療における |  |  |  |  |
| **月1回**程度の受診 |
| オンライン診療における |  |  |  |  |
| **週1回**程度の受診 |
| オンライン診療における |  |  |  |  |
| **2週に1回**程度の受診 |
| オンライン診療における |  |  |  |  |
| **月1回**程度の受診 |

8.

どのような科におけるオンライン診療の利⽤でしたか/ですか。（いくつでも）*回答必須

□内科

□外科

□⼩児科

□産婦⼈科・⽣殖医療

□精神科・⼼療内科

□⽪膚科

□眼科

□⽿⿐咽喉科

□泌尿器科

□整形外科

□形成外科・美容外科

□その他

9.

どのような場⾯でのオンライン診療の利⽤でしたか（ですか）。（いくつでも）*回答必須

□検査の結果説明

□定期的な診察

□いつもと同じ薬の処⽅

□発熱外来など、何らかの感染症が想定される場⾯での受診

□感染症以外の、急に発症した疾患（急性疾患）についての受診

□その疾患を専⾨とする医師が近くにいない場合

□病院への通院が困難な場合（移動困難、遠隔地居住など）

□受診するべきかどうかについての相談

□セカンドオピニオン

□栄養指導

□⺟親学級、在宅医療導⼊時カンファレンスなど、⼀般的な診察（患者⼀⼈と医師⼀⼈という形式）以外の機会

□その他（⾃由回答）

10.

オンライン診療を利⽤したきっかけは何ですか。（ひとつだけ）*回答必須

○オンライン診療をあなた あるいは あなたの家族側から希望し、対応可能な医療施設を探した。

○対⾯で診療を受けていた医師 あるいは 医療施設から、オンライン診療の選択肢を提⽰された。

11.

オンライン診療を⾏った場合、対⾯診療と⽐較して以下の項⽬がどうか、あなた（回答者）の評価を教えてください。*回答必須 ⾦銭的負担には、診療費、交通費、回線料⾦など受診に関連した諸々を含みます。

|  | オンライン診療の方が | オンライン診療の方が | 対面診療と  概ね | オンライン診療の方が | オンライン診療の方が |
| --- | --- | --- | --- | --- | --- |
|  | 明らかに  小さい | やや小さい | 同等である | やや大きい | 明らかに  大きい |
| 時間的負担 |  |  |  |  |  |
| 身体的負担 |  |  |  |  |  |
| 精神的負担 |  |  |  |  |  |
| 金銭的負担 |  |  |  |  |  |

12.

オンライン診療の際に、ソフトウェアやアプリのダウンロード及び操作で困ったことはありますか。（ひとつだけ）*回答必須

○全く困らなかった

○少し困った

○⾮常に困った

13.

オンライン診療の際に、繋がらない/回線が途切れるなど、通信環境でトラブルが⽣じた経験はありますか。（ひとつだけ）*回答必須

○トラブルの経験はない

○トラブルの経験がある

14.

あなた（回答者）のオンライン診療の満⾜度は以下のうちのどれですか。（ひとつだけ）*回答必須

○とても満⾜している

○だいたい満⾜している

○あまり満⾜していない

○全く満⾜していない

15.

オンライン診療の普及を阻害する要因として、大きいと思うものを下記の中から3つ選択してください。*回答必須

□オンライン診療では、医療機関側の事務的な手続きが増えてしまうため

□オンライン診療では、患者側の手間が増えてしまうため

□オンライン診療では、医療機関側の金銭的負担が大きいため

□オンライン診療では、患者の金銭的負担が大きいため

□オンライン診療のためのシステムや通信環境の構築が、医療機関にとって困難であるため

□オンライン診療のためのアプリダウンロード・通信環境の構築などが、患者にとって困難であるため

□オンライン診療では、医師の診察内容に不安が残るため

□オンラインよりも、対面の方が話しやすいため

□オンライン診療では、検査や処置が必要になった場合に結局通院が必要になってしまうため

□オンライン診療に適した患者があまりいないため

□オンライン診療による個人情報漏洩が心配であるため

□オンライン診療という診療形態自体があまり知られていないため

□オンライン診療という実施形態は知られているが、どういった場合にオンライン診療が適しているのか/希望してよいのかわからないため

□オンライン診療という実施形態は知られているが、オンライン診療を実施している機関がどここかわからないため

□対面診療の満足度が高く、オンライン診療を必要としている人が少ないため

□対面診療の満足度に関わらず、現状維持を望む心理が働くため

16.

その他、オンライン診療について、便利である点、困っている点、今後期待する点など、何かお考えがあればお教えください。

**Main questionnaire (2)**

1.

事前調査で、

**・あなた自身あるいはご家族は、定期通院中である**

**・あなた自身あるいはご家族は、オンライン診療の経験がない**

と回答された方向けの設問です。当てはまっていない場合には、現時点で回答を中断してください。

【ご回答前の注意事項｜必ずお読みください】 ・定期通院されているご自身、あるいはあなたが付き添って定期受診するご家族のうち、どなたか任意のお一人を想定し、ご回答ください。 上記の注意事項をお読みいただいた上で、どなたについてご回答されますか。

（ひとつだけ）*回答必須

○あなた自身が定期受診をしており、あなた自身について回答する

○あなたが付き添って定期受診されている方について回答する

分岐条件 - Q1でいずれかを選択した選択肢： [2.あなたが付き添って定期受診されている方について回答する]

2.

あなたが付き添って定期受診されている方は何歳ですか？整数で記載してください。

*回答必須

定期受診されている方の年齢 *　　歳

3.

現在の受診頻度として、最もあてはまるものを選択してください。（ひとつだけ）*回答必須

○週1回程度

○2週に1回程度

○月1回程度

○2ヶ月に1回程度

○3か月に1回程度

4.

現在通院している医療機関への片道の所要時間として、最もあてはまるものを選択してください。（ひとつだけ）*回答必須

【最初の質問で「あなたが付き添って定期受診されている方について回答する」を選択した方へ】 あなた（回答者）と定期受診されている方とで居住地が異なる場合、あなた（回答者）ではなく定期受診されている方の所用時間をご回答ください。

○30分未満

○30分以上1時間未満

○1時間以上2時間未満

○2時間以上

5.

お住まいの地域には、どのくらい多くの医療機関がありますか。あなたの印象を教えてください（ひとつだけ）*回答必須

【最初の質問で「あなたが付き添って定期されている方について回答する」を選択した方へ】 あなた（回答者）と定期受診されている方とで居住地が異なる場合、あなた（回答者）ではなく定期受診されている方のお住まいの地域についてご回答ください。

○近くに医療機関が数多く存在する

○近くに医療機関がそれなりの数存在する

○近くに医療機関はあまり存在しない

6.

通院における手間（移動の大変さ、所要時間、仕事の調整、子供の世話の調整等）について、あなた（回答者）はどのように感じますか。 （ひとつだけ）*回答必須

【最初の質問で「あなたが付き添って定期されている方について回答する」を選択した方へ】 どなたかの定期受診へ付き添う場合のあなた（回答者）の手間を想定して、ご回答ください。

○かなり手間がかかる

○やや手間がかかる

○あまり手間はかからない

○全く手間はかからない

7.

以下の項目のそれぞれについて、選択肢から最も当てはまるものを一つ選択してください。*回答必須

【最初の質問で「あなたが受診に付き添っている方について回答する」を選択した方へ】 あなた（回答者）が定期受診へ付き添う場合を想定して、ご回答ください。

|  | 全く問題なく 許容可能である | 抵抗はあるが 許容可能である | できれば 避けたい | 絶対に 許容できない |
| --- | --- | --- | --- | --- |
| 対面診療における |  |  |  |  |
| **週1回**程度の受診 |
| 対面診療における |  |  |  |  |
| **2週に1回**程度の受診 |
| 対面診療における |  |  |  |  |
| **月1回**程度の受診 |
| オンライン診療における |  |  |  |  |
| **週1回**程度の受診 |
| オンライン診療における |  |  |  |  |
| **2週に1回**程度の受診 |
| オンライン診療における |  |  |  |  |
| **月1回**程度の受診 |

8.

オンライン診療を行った場合、対面診療と比較して以下の項目がどうなると思うか、あなたの評価を教えてください。（各項目につき単一回答）*回答必須

金銭的負担には、診療費、交通費、回線料金など受診に関連した諸々を含みます。

|  | オンライン診療の方が | オンライン診療の方が | 対面診療と  概ね | オンライン診療の方が | オンライン診療の方が |
| --- | --- | --- | --- | --- | --- |
|  | 明らかに  小さい | やや小さい | 同等である | やや大きい | 明らかに  大きい |
| 時間的負担 |  |  |  |  |  |
| 身体的負担 |  |  |  |  |  |
| 精神的負担 |  |  |  |  |  |
| 金銭的負担 |  |  |  |  |  |

9.

以下の科のうち、オンライン診療を利用してもよいと思うものを選択してください。

（いくつでも）*回答必須

□内科

□外科

□小児科

□産婦人科・生殖医療

□精神科・心療内科

□皮膚科

□眼科

□耳鼻咽喉科

□泌尿器科

□整形外科

□形成外科・美容外科

□あてはまるものはない

10.

以下の場⾯のうち、オンライン診療を利⽤しても良いと思うものを選択してください。

（いくつでも）*回答必須

□検査の結果説明

□定期的な診察

□いつもと同じ薬の処⽅

□発熱外来など、何らかの感染症が想定される場⾯での受診

□感染症以外の、急に発症した疾患（急性疾患）についての受診

□その疾患を専⾨とする医師が近くにいない場合

□病院への通院が困難な場合（移動困難、遠隔地居住など）

□受診するべきかどうかについての相談

□セカンドオピニオン

□栄養指導

□⺟親学級、在宅医療導⼊時カンファレンスなど、⼀般的な診察（患者⼀⼈と医師⼀⼈という形式）以外の機会

□あてはまるものはない

11.

以下のシチュエーションにおいて、オンライン診療を利用しても良いと思う場合、チェックを付けてください。（いくつでも）*回答必須

□対面で診療を受けていた医師あるいは医療施設から、オンライン診療の選択肢を提示される場合

□対面で診療を受けていた医師の異動に伴い、オンライン診療に移行した（オンライン診療でない場合には担当医変更となるため）場合

□あてはまるものはない

12.

あなたは自身の端末（パソコン、スマートフォン、タブレット等）を用いてのオンライン診療の受診が実行可能だと思いますか。（ひとつだけ）*回答必須

○準備を含め、一人で可能だと思う

○家族等の手伝いを受ければ可能だと思う

○困難であると思う

13.

オンライン診療の普及を阻害する要因として、大きいと思うものを下記の中から3つ選択してください。*回答必須

□オンライン診療では、医療機関側の事務的な手続きが増えてしまうため

□オンライン診療では、患者側の手間が増えてしまうため

□オンライン診療では、医療機関側の金銭的負担が大きいため

□オンライン診療では、患者の金銭的負担が大きいため

□オンライン診療のためのシステムや通信環境の構築が、医療機関にとって困難であるため

□オンライン診療のためのアプリダウンロード・通信環境の構築などが、患者にとって困難であるため

□オンライン診療では、医師の診察内容に不安が残るため

□オンラインよりも、対面の方が話しやすいため

□オンライン診療では、検査や処置が必要になった場合に結局通院が必要になってしまうため

□オンライン診療に適した患者があまりいないため

□オンライン診療による個人情報漏洩が心配であるため

□オンライン診療という診療形態自体があまり知られていないため

□オンライン診療という実施形態は知られているが、どういった場合にオンライン診療が適しているのか/希望してよいのかわからないため

□オンライン診療という実施形態は知られているが、オンライン診療を実施している機関がどここかわからないため

□対面診療の満足度が高く、オンライン診療を必要としている人が少ないため

□対面診療の満足度に関わらず、現状維持を望む心理が働くため

14.

その他、オンライン診療について、便利である点、困っている点、今後期待する点など、何かお考えがあればお教えください。

**Main questionnaire (3)**

1 事前調査で、

**・あなた⾃⾝は定期通院中ではなく、また、ご家族の定期通院にも付き添っていない**

**・オンライン診療の経験がある**

と回答された⽅向けの設問です。当てはまっていない場合には、現時点で回答を中断してください。

お住まいの地域には、どのくらい多くの医療機関がありますか。あなたの印象を教えてください。（ひとつだけ）*回答必須

○近くに医療機関が数多く存在する

○近くに医療機関がそれなりの数存在する

○近くに医療機関はあまり存在しない

2.

通院における⼿間（移動の⼤変さ、所要時間、仕事の調整、⼦供の世話の調整等）について、どのように感じますか。（ひとつだけ）*回答必須

○かなり⼿間がかかる

○やや⼿間がかかる

○あまり⼿間はかからない

○全く⼿間はかからない

3.

以下の項⽬のそれぞれについて、選択肢から最も当てはまるものを⼀つ選択してください。*回答必須

|  | 全く問題なく 許容可能である | 抵抗はあるが 許容可能である | できれば 避けたい | 絶対に 許容できない |
| --- | --- | --- | --- | --- |
| 対面診療における |  |  |  |  |
| **週1回**程度の受診 |
| 対面診療における |  |  |  |  |
| **2週に1回**程度の受診 |
| 対面診療における |  |  |  |  |
| **月1回**程度の受診 |
| オンライン診療における |  |  |  |  |
| **週1回**程度の受診 |
| オンライン診療における |  |  |  |  |
| **2週に1回**程度の受診 |
| オンライン診療における |  |  |  |  |
| **月1回**程度の受診 |

4.

どのような科におけるオンライン診療の利⽤でしたか/ですか。（いくつでも）*回答必須

□内科

□外科

□⼩児科

□産婦⼈科・⽣殖医療

□精神科・⼼療内科

□⽪膚科

□眼科

□⽿⿐咽喉科

□泌尿器科

□整形外科

□形成外科・美容外科

□その他

5.

どのような場⾯でのオンライン診療の利⽤でしたか/ですか。（いくつでも）*回答必須

□検査の結果説明

□定期的な診察

□いつもと同じ薬の処⽅

□発熱外来など、何らかの感染症が想定される場⾯での受診

□感染症以外の、急に発症した疾患（急性疾患）についての受診

□その疾患を専⾨とする医師が近くにいない場合

□病院への通院が困難な場合（移動困難、遠隔地居住など）

□受診するべきかどうかについての相談

□セカンドオピニオン

□栄養指導

□母親学級、在宅医療導⼊時カンファレンスなど、⼀般的な診察（患者⼀⼈と医師⼀⼈という形式）以外の機会

□その他（⾃由回答）

6.

オンライン診療を利⽤したきっかけは何ですか。（ひとつだけ）*回答必須

○オンライン診療をあなたあるいはあなたの家族側から希望し、対応可能な医療施設を探した。

○対⾯で診療を受けていた医師あるいは医療施設から、オンライン診療の選択肢を提⽰された。

○その他

7.

オンライン診療を⾏った場合、対⾯診療と⽐較して以下の項⽬がどうか、あなたの評価を教えてください。*回答必須 ⾦銭的負担には、診療費、交通費、回線料⾦など受診に関連した諸々を含みます。

|  | オンライン診療の方が | オンライン診療の方が | 対面診療と  概ね | オンライン診療の方が | オンライン診療の方が |
| --- | --- | --- | --- | --- | --- |
|  | 明らかに  小さい | やや小さい | 同等である | やや大きい | 明らかに  大きい |
| 時間的負担 |  |  |  |  |  |
| 身体的負担 |  |  |  |  |  |
| 精神的負担 |  |  |  |  |  |
| 金銭的負担 |  |  |  |  |  |

8.

オンライン診療の際に、ソフトウェアやアプリのダウンロード及び操作で困ったことはありますか。（ひとつだけ）*回答必須

○全く困らなかった

○少し困った

○⾮常に困った

9.

オンライン診療の際に、繋がらない/回線が途切れるなど、通信環境でトラブルが⽣じた経験はありますか。（ひとつだけ）*回答必須

○トラブルの経験はない

○トラブルの経験がある

10.

オンライン診療の満⾜度は以下のうちのどれですか。（ひとつだけ）*回答必須

○とても満⾜している

○だいたい満⾜している

○あまり満⾜していない

○全く満⾜していない

11.

オンライン診療の普及を阻害する要因として、⼤きいと思うものを下記の中から３つ選択してください。*回答必須

□オンライン診療では、医療機関側の事務的な手続きが増えてしまうため

□オンライン診療では、患者側の手間が増えてしまうため

□オンライン診療では、医療機関側の金銭的負担が大きいため

□オンライン診療では、患者の金銭的負担が大きいため

□オンライン診療のためのシステムや通信環境の構築が、医療機関にとって困難であるため

□オンライン診療のためのアプリダウンロード・通信環境の構築などが、患者にとって困難であるため

□オンライン診療では、医師の診察内容に不安が残るため

□オンラインよりも、対面の方が話しやすいため

□オンライン診療では、検査や処置が必要になった場合に結局通院が必要になってしまうため

□オンライン診療に適した患者があまりいないため

□オンライン診療による個人情報漏洩が心配であるため

□オンライン診療という診療形態自体があまり知られていないため

□オンライン診療という実施形態は知られているが、どういった場合にオンライン診療が適しているのか/希望してよいのかわからないため

□オンライン診療という実施形態は知られているが、オンライン診療を実施している機関がどここかわからないため

□対面診療の満足度が高く、オンライン診療を必要としている人が少ないため

□対面診療の満足度に関わらず、現状維持を望む心理が働くため

12.

その他、オンライン診療について、便利である点、困っている点、今後期待する点など、何かお考えがあればお教えください。

**Main questionnaire (4)**

1

事前調査で、

**・あなた⾃⾝は定期通院中ではなく、また、ご家族の定期通院にも付き添っていない**

**・オンライン診療の経験がない**

と回答された⽅向けの設問です。当てはまっていない場合には、現時点で回答を中断してください。

お住まいの地域には、どのくらい多くの医療機関がありますか。あなたの印象を教えてください。（ひとつだけ）*回答必須

○近くに医療機関が数多く存在する

○近くに医療機関がそれなりの数存在する

○近くに医療機関はあまり存在しない

2.

通院における⼿間（移動の⼤変さ、所要時間、仕事の調整、⼦供の世話の調整等）について、どのように感じますか。（ひとつだけ）*回答必須

○かなり⼿間がかかる

○やや⼿間がかかる

○あまり⼿間はかからない

○全く⼿間はかからない

3.

以下の項⽬のそれぞれについて、選択肢から最も当てはまるものを⼀つ選択してください。*回答必須

|  | 全く問題なく 許容可能である | 抵抗はあるが 許容可能である | できれば 避けたい | 絶対に 許容できない |
| --- | --- | --- | --- | --- |
| 対面診療における |  |  |  |  |
| **週1回**程度の受診 |
| 対面診療における |  |  |  |  |
| **2週に1回**程度の受診 |
| 対面診療における |  |  |  |  |
| **月1回**程度の受診 |
| オンライン診療における |  |  |  |  |
| **週1回**程度の受診 |
| オンライン診療における |  |  |  |  |
| **2週に1回**程度の受診 |
| オンライン診療における |  |  |  |  |
| **月1回**程度の受診 |

4.

オンライン診療を⾏った場合、対⾯診療と⽐較して以下の項⽬がどうなると思うか、あなたの考えを教えてください。*回答必須 ⾦銭的負担には、診療費、交通費、回線料⾦など受診に関連した諸々を含みます。

|  | オンライン診療の方が | オンライン診療の方が | 対面診療と  概ね | オンライン診療の方が | オンライン診療の方が |
| --- | --- | --- | --- | --- | --- |
|  | 明らかに  小さい | やや小さい | 同等である | やや大きい | 明らかに  大きい |
| 時間的負担 |  |  |  |  |  |
| 身体的負担 |  |  |  |  |  |
| 精神的負担 |  |  |  |  |  |
| 金銭的負担 |  |  |  |  |  |

5.

以下の科のうち、オンライン診療を利⽤してもよいと思うものを選択してください。（いくつでも）*回答必須

□内科

□外科

□⼩児科

□産婦⼈科・⽣殖医療

□精神科・⼼療内科

□⽪膚科

□眼科

□⽿⿐咽喉科

□泌尿器科

□整形外科

□形成外科・美容外科

□あてはまるものはない

6.

以下の場⾯のうち、オンライン診療を利⽤してもよいと思うものを選択してください。（いくつでも）*回答必須

□検査の結果説明

□定期的な診察

□いつもと同じ薬の処⽅

□発熱外来など、何らかの感染症が想定される場⾯での受診

□感染症以外の、急に発症した疾患（急性疾患）についての受診

□その疾患を専⾨とする医師が近くにいない場合

□病院への通院が困難な場合（移動困難、遠隔地居住など）

□受診するべきかどうかについての相談

□セカンドオピニオン

□栄養指導

□⺟親学級、在宅医療導⼊時カンファレンスなど、⼀般的な診察（患者⼀⼈と医師⼀⼈という形式）以外の機会

□あてはまるものはない

7.

以下のシチュエーションのうち、オンライン診療を利⽤してもよいと思うものを選択してください。（いくつでも）*回答必須

□対⾯で診療を受けていた医師あるいは医療施設から、オンライン診療の選択肢を提⽰される場合

□対⾯で診療を受けていた医師の異動に伴い、オンライン診療に移⾏した（オンライン診療でない場合には担当医変更となるため）場合

□あてはまるものはない

8.

あなたは⾃⾝の端末（パソコン、スマートフォン、タブレット等）を⽤いてのオンライン診療の受診が実⾏可能だと思いますか。（ひとつだけ）*回答必須

○準備を含め、⼀⼈で可能だと思う

○家族等の⼿伝いを受ければ可能だと思う

○困難であると思う

9.

オンライン診療の普及を阻害する要因として、⼤きいと思うものを下記の中から3つ選択してください。*回答必須

□オンライン診療では、医療機関側の事務的な⼿続きが増えてしまうため

□オンライン診療では、患者側の⼿間が増えてしまうため

□オンライン診療では、医療機関側の⾦銭的負担が⼤きいため

□オンライン診療では、患者の⾦銭的負担が⼤きいため

□オンライン診療のためのシステムや通信環境の構築が、医療機関にとって困難であるため

□オンライン診療のためのアプリダウンロード・通信環境の構築などが、患者にとって困難であるため

□オンライン診療では、医師の診察内容に不安が残るため

□オンラインよりも、対⾯の⽅が話しやすいため

□オンライン診療では、検査や処置が必要になった場合に結局通院が必要になってしまうため

□オンライン診療に適した患者があまりいないため

□オンライン診療による個⼈情報漏洩が⼼配であるため

□オンライン診療という診療形態⾃体があまり知られていないため

□オンライン診療という実施形態は知られているが、どういった場合にオンライン診療が適しているのか/希望してよいのかわからないため

□オンライン診療という実施形態は知られているが、オンライン診療を実施している機関がどこかわからないため

□対⾯診療の満⾜度が⾼く、オンライン診療を必要としている⼈が少ないため

□対⾯診療の満⾜度に関わらず、現状維持を望む⼼理が働くため

10.

その他、オンライン診療について、便利である点、困っている点、今後期待する点など、何かお考えがあればお教えください。
