# Supplementary material for: Issues in the Adoption of Online Medical Care: Cross-Sectional Questionnaire Survey
Source: J Med Internet Res. 2024 Nov 1;26:e64159. doi: 10.2196/64159 (PMC11568393; doi:10.2196/64159)
Supplement: Multimedia Appendix 2 [file jmir_v26i1e64159_app2.doc]

**Appendix 1. English-translated questionnaire for patients and healthy individuals.**

Screening questionnaire -----------------------------------------------------------p.2

Main questionnaire (1)-------------------------------------------------------------p.4

Main questionnaire (2)-------------------------------------------------------------p.9

Main questionnaire (3)-------------------------------------------------------------p.14

Main questionnaire (4)-------------------------------------------------------------p.18

**Screening questionnaire**

１．

For each of the following, please check all that apply. A regular visit means a visit at least once every 3 months or more.

A. You regularly visits some medical facility.

B. Accompanying a pre-elementary school child for regular visits to a medical facility.

C. Accompanying a child of elementary to junior high school age to regular visits to a medical facility.

D. Accompanying a family member, roommate, or other person 15 years of age or older to regular visits to a medical facility.

E. None that apply.

2.

Have you ever used online medical care (hereafter referred to as OMC) ("real-time medical services such as examination, diagnosis, communication of diagnosis results, and prescriptions between a doctor and a patient through information and communication devices", excluding telephone consultations)?

A. I have used it.

B. I have never used it.

Branching Condition – If the responder selected Q1-B, C, or D, Q3 appeared.

3.

Have your family members ever used OMC?

A. I have used it.

B. I have never used it.

Branching Condition – If the respondents selected Q1-A, Q4 appeared.

4.

Which of the following is your current regular doctor's appointment?

A. Clinic

B. General hospitals

C. Large hospitals such as university hospitals, center hospitals, etc. (e.g., hospitals with specific functions, regional medical support hospitals, etc.)

D. Not currently receiving treatment at the hospital

Branching Condition – If the respondents selected Q1-B, C, and D, Q5 appeared.

5.

Which of the following is your family member's current regular doctor's appointment? If you are accompanied by more than one family member, please answer for one of them.

A. Clinic

B. General hospitals

C. Large hospitals such as university hospitals, center hospitals, etc. (e.g., hospitals with specific functions, regional medical support hospitals, etc.)

D. Not currently receiving treatment at the hospital

**Main questionnaire (1)**

1.

This questionnaire is for people who answered the following in the screening survey:

**- You or a family member are currently visiting a doctor on a regular basis.**

**- You or a family member have experience with online medical care (hereafter referred to as OMC).**

If the above condition does not apply to you, please stop answering at this time. Please answer this question assuming that you and/or your family member who you accompany are a regular visitor to a medical facility.

After reading the above notes, who would you like to answer about?

A. You yourself are regularly visiting a medical facility, and you will answer about yourself.

B. You will answer about the family member who you accompany to his/her regular visits.

Branching condition – If the respondents selected Q1-B, Q2 appeared.

2.

How old is the person you are accompanying for regular visits?

Age of persons receiving regular checkups　 * 　Years Old

3.

Please select the most applicable frequency of your current visit.

A. Once a week

B. Once every two weeks

C. Once a month

D. Once every two months

E. Once every three months

4.

Please select the most applicable time required for a one-way trip to your current medical facility.

A. Less than 30 minutes

B. More than 30 minutes but less than 1 hour

C. Over 1 hour but less than 2 hours

D. Over 2 hours

5.

How many medical facilities are in your area? Please tell us your impression.

A. Numerous medical facilities nearby

B. A certain number of medical facilities nearby.

C. Not many medical facilities nearby.

6.

How do you feel about the time and effort when going to a doctor's office?

A. A lot of time and effort is needed when going to a doctor's office.

B. Some time and effort is needed when going to a doctor's office.

C. Not much time and effort is needed when going to a doctor's office.

D. No time and effort is needed when going to a doctor's office.

7.

For each of the following items, please select the one that best fits your choice.

|  | Acceptable with no problems at all | Resistance is present but acceptable. | Avoid if possible | Absolutely unacceptable |
| --- | --- | --- | --- | --- |
| About once a week in face-to-face consultations (hereafter referred to as FMC) |  |  |  |  |
| About once every two weeks in FMC |  |  |  |  |
| About once a month in FMC |  |  |  |  |
| About once a week visit in OMC |  |  |  |  |
| About once every two weeks visit in OMC |  |  |  |  |
| About once a month visit in OMC |  |  |  |  |

8.

In which department did/do you use OMC?

A. Internal Medicine

B. Surgery

C. Pediatrics

D. Obstetrics and Gynecology/Reproductive Medicine

E. Psychiatry/Psychosomatic medicine

F. Dermatology

G. Ophthalmology

H. Otorhinolaryngology

I. Urology

J. Orthopedic surgery

K. Plastic surgery/cosmetic surgery

L. Others

9.

In what situations did /do you use OMC ?

A. Explanation of test results

B. Regular medical checkup

C. Doing the same medication as usual

D. Consultation at the scene of a possible infectious disease such as a fever outpatient clinic

E. Consultation for sudden onset of illness (acute illness) other than infectious disease

F. When there is no doctor nearby who specializes in that disease

G. When it is difficult to go to a hospital (difficulty in transportation, living in a remote area, etc.)

H. Consultation on whether or not to see a doctor

I. Second opinion

J. Nutritional guidance

K. Opportunities other than general consultation (patient and doctor) such as, parent classes, conferences at the start of home medical care, etc.

L. Others.

10.

What made you decide to use OMC?

A. You or your family members have wanted OMC and have searched for a medical facility that can provide it.

B. The doctor or medical facility where you have been receiving FMC has offered you the option of OMC.

11.

Please tell us how you would rate the following items if you receive OMC compared to FMC.

Financial burden includes medical fees, transportation costs, connection fees, and other costs related to the visit.

|  | Obviously smaller in OMC | Slightly smaller in OMC | Roughly equivalent to FMC | Slightly larger in OMC | Obviously larger in OMC |
| --- | --- | --- | --- | --- | --- |
| time burden |  |  |  |  |  |
| physical burden |  |  |  |  |  |
| mental burden |  |  |  |  |  |
| financial burden |  |  |  |  |  |

12.

Have you ever had any problems downloading and operating software or apps during OMC?

A. No, I had no trouble at all.

B. Slightly troubled

C. Very troubled

13.

Have you ever experienced any trouble with the communication environment during OMC, such as disconnection?

A. No, I have never had a problem.

B. Yes, I have experienced problems.

14.

At which of the following would you rate your satisfaction with OMC ?

A. Very satisfied with it

B. Roughly satisfied with it

C. Not really satisfied with it

D. Not at all satisfied with it

15.

Please select three factors from the following that you think are major impediments to the widespread use of OMC.

A. OMC increases administrative procedures on the part of medical institutions.

B. OMC requires more time and effort on the part of the patient.

C. OMC places a heavy financial burden on the medical institution.

D. OMC imposes a heavy financial burden on patients.

E. Difficult for the medical institution to construct a system and communication environment for OMC.

F. Difficult for patients to download applications and build a communication environment for OMC.

G. People concern about the content of the doctor's examination in OMC

H. Talk face-to-face is easier than that online

I. OMC requires patients to make a hospital visit when tests or procedures are needed

J. There are not many patients who are suitable for OMC.

K. People concern about the leakage of personal information through OMC

L. Because OMC itself is not well known

M. OMC are known, but people do not know when OMC are appropriate or desired.

N. OMC are known, but people do not know which institutions offer OMC.

O. Because the level of satisfaction with FMC is high and there are not many people who need OMC.

P. Regardless of the level of satisfaction with FMC, people tend to maintain the status quo.

16.

Please share any other thoughts you have about OMC, including what you find convenient, what you have trouble with, or what you hope to see in the future.

**Main questionnaire (2)**

1.

This questionnaire is for people who answered the following in the screening survey:

**- You or a family member are currently visiting a doctor on a regular basis.**

**- You or a family member have no experience with online medical care (hereafter referred to as OMC).**

If the above condition does not apply to you, please stop answering at this time. Please answer this question assuming that you are a regular visitor to the clinic or that you are a family member who you accompany to regular visits. After reading the above notes, who would you like to answer about?

A. You yourself are visiting regularly, and you will answer about yourself.

B. You will answer about the family member who you accompany to his/her regular visits.

Branching condition – If the respondents selected Q1-B, Q2 appeared.

2.

How old is the person you are accompanying for regular visits?

Age of persons receiving regular checkups　 * 　Years Old

3.

Please select the most applicable frequency of your current visit.

A. Once a week

B. Once every two weeks

C. Once a month

D. Once every two months

E. Once every three months

4.

Please select the most applicable time required for a one-way trip to your current medical facility.

A. Less than 30 minutes

B. More than 30 minutes but less than 1 hour

C. Over 1 hour but less than 2 hours

D. Over 2 hours

5.

How many medical facilities are in your area?

A. Numerous medical facilities nearby

B. A certain number of medical facilities nearby

C. Not many medical facilities nearby

6.

How do you feel about the time and effort when going to a doctor's office?

A. A lot of time and effort is needed when going to a doctor's office.

B. Some time and effort is needed when going to a doctor's office.

C. Not much time and effort is needed when going to a doctor's office.

D. No time and effort is needed when going to a doctor's office.

7.

For each of the following items, please select the one that best fits your choice.

|  | Acceptable with no problems at all | Resistance is present but acceptable. | Avoid if possible | Absolutely unacceptable |
| --- | --- | --- | --- | --- |
| About once a week in face-to-face consultations (hereafter referred to as FMC) |  |  |  |  |
| About once every two weeks in FMC |  |  |  |  |
| About once a month in FMC |  |  |  |  |
| About once a week visit in OMC |  |  |  |  |
| About once every two weeks visit in OMC |  |  |  |  |
| About once a month visit in OMC |  |  |  |  |

8.

Please tell us how you would rate the following items if you receive OMC compared to FMC.

Financial burden includes medical fees, transportation costs, connection fees, and other costs related to the visit.

|  | Obviously smaller in OMC | Slightly smaller in OMC | Roughly equivalent to FMC | Slightly larger in OMC | Obviously larger in OMC |
| --- | --- | --- | --- | --- | --- |
| time burden |  |  |  |  |  |
| physical burden |  |  |  |  |  |
| mental burden |  |  |  |  |  |
| financial burden |  |  |  |  |  |

9.

Please select which of the following departments you would be willing to use OMC.

A. Internal Medicine

B. Surgery

C. Pediatrics

D. Obstetrics and Gynecology/Reproductive Medicine

E. Psychiatry/Psychosomatic medicine

F. Dermatology

G. Ophthalmology

H. Otorhinolaryngology

I. Urology

J. Orthopedic surgery

K. Plastic surgery/cosmetic surgery

L. None that apply

10.

In the following situations, please check the box you think it is acceptable to use OMC.

A. Explanation of test results

B. Regular medical checkup

C. Doing the same medication as usual

D. Consultation at the scene of a possible infectious disease such as a fever outpatient clinic

E. Consultation for sudden onset of illness (acute illness) other than infectious disease

F. When there is no doctor nearby who specializes in that disease

G. When it is difficult to go to a hospital (difficulty in transportation, living in a remote area, etc.)

H. Consultation on whether or not to see a doctor

I. Second opinion

J. Nutritional guidance

K. Opportunities other than general consultation (patient and doctor) such as, parent classes, conferences at the start of home medical care, etc.

L. None that apply.

11.

In the following situations, please check the box if you would be willing to use OMC.

A. When a doctor or medical facility that has been providing FMC offers the option of OMC.

B. When the doctor you were receiving FMC has moved, and the option of OMC is offered in order to continue seeing him/her.

C. None that apply.

12.

Do you think it is feasible for you to receive OMC using your own device (computer, smartphone, tablet, etc.)?

A. I think I can do it alone, including preparation.

B. With help from family members, etc., I think it is feasible.

C. Difficult to do.

13.

Please select three factors from the following that you think are major impediments to the widespread use of OMC.

A. OMC increases administrative procedures on the part of medical institutions.

B. OMC requires more time and effort on the part of the patient.

C. OMC places a heavy financial burden on the medical institution.

D. OMC imposes a heavy financial burden on patients.

E. Difficult for the medical institution to construct a system and communication environment for OMC.

F. Difficult for patients to download applications and build a communication environment for OMC.

G. People concern about the content of the doctor's examination in OMC

H. Talk face-to-face is easier than that online

I. OMC requires patients to make a hospital visit when tests or procedures are needed

J. There are not many patients who are suitable for OMC.

K. People concern about the leakage of personal information through OMC

L. Because OMC itself is not well known

M. OMC are known, but people do not know when OMC are appropriate or desired.

N. OMC are known, but people do not know which institutions offer OMC.

O. Because the level of satisfaction with FMC is high and there are not many people who need OMC.

P. Regardless of the level of satisfaction with FMC, people tend to maintain the status quo.

14.

Please share any other thoughts you have about OMC, including what you find convenient, what you have trouble with, or what you hope to see in the future.

**Main questionnaire (3)**

1.

This questionnaire is for people who answered the following in the screening survey:

**- You or a family member are not currently visiting a doctor on a regular basis.**

**- You or a family member have experience with online medical care (hereafter referred to as OMC).**

If the above condition does not apply to you, please stop answering at this time.

How many medical facilities are in your area? Please tell us your impression.

A. Numerous medical facilities nearby

B. A certain number of medical facilities nearby

C. Not many medical facilities nearby

2.

How do you feel about the time and effort when going to a doctor's office?

A. A lot of time and effort is needed when going to a doctor's office.

B. Some time and effort is needed when going to a doctor's office.

C. Not much time and effort is needed when going to a doctor's office.

D. No time and effort is needed when going to a doctor's office.

3.

For each of the following items, please select the one that best fits your choice.

|  | Acceptable with no problems at all | Resistance is present but acceptable. | Avoid if possible | Absolutely unacceptable |
| --- | --- | --- | --- | --- |
| About once a week in face-to-face consultations (hereafter referred to as FMC) |  |  |  |  |
| About once every two weeks in FMC |  |  |  |  |
| About once a month in FMC |  |  |  |  |
| About once a week visit in OMC |  |  |  |  |
| About once every two weeks visit in OMC |  |  |  |  |
| About once a month visit in OMC |  |  |  |  |

4.

In which department did/do you use OMC?

A. Internal Medicine

B. Surgery

C. Pediatrics

D. Obstetrics and Gynecology/Reproductive Medicine

E. Psychiatry/Psychosomatic medicine

F. Dermatology

G. Ophthalmology

H. Otorhinolaryngology

I. Urology

J. Orthopedic surgery

K. Plastic surgery/cosmetic surgery

L. Others

5.

In what situations did /do you use OMC?

A. Explanation of test results

B. Regular medical checkup

C. Doing the same medication as usual

D. Consultation at the scene of a possible infectious disease such as a fever outpatient clinic

E. Consultation for sudden onset of illness (acute illness) other than infectious disease

F. When there is no doctor nearby who specializes in that disease

G. When it is difficult to go to a hospital (difficulty in transportation, living in a remote area, etc.)

H. Consultation on whether or not to see a doctor

I. Second opinion

J. Nutritional guidance

K. Opportunities other than general consultation (patient and doctor) such as, parent classes, conferences at the start of home medical care, etc.

L. Others.

6.

What made you decide to use OMC?

A. You or your family members have wanted OMC and have searched for a medical facility that can provide it.

B. The doctor or medical facility where you have been receiving FMC has offered you the option of OMC.

C. Others

7.

Please tell us how you would rate the following items if you receive OMC compared to FMC.

Financial burden includes medical fees, transportation costs, connection fees, and other costs related to the visit.

|  | Obviously smaller in OMC | Slightly smaller in OMC | Roughly equivalent to FMC | Slightly larger in OMC | Obviously larger in OMC |
| --- | --- | --- | --- | --- | --- |
| time burden |  |  |  |  |  |
| physical burden |  |  |  |  |  |
| mental strain |  |  |  |  |  |
| financial burden |  |  |  |  |  |

8.

Have you ever had any problems downloading and operating software or apps during OMC?

A. No, I had no trouble at all.

B. Slightly troubled

C. Very troubled

9.

Have you ever experienced any trouble with the communication environment during OMC, such as disconnection?

A. No, I have never had a problem.

B. Yes, I have experienced problems.

10.

At which of the following would you rate your satisfaction with OMC ?

A. Very satisfied with it

B. Roughly satisfied with it

C. Not really satisfied with it

D. Not at all satisfied with it

11.

Please select three factors from the following that you think are major impediments to the widespread use of OMC.

A. OMC increases administrative procedures on the part of medical institutions.

B. OMC requires more time and effort on the part of the patient.

C. OMC places a heavy financial burden on the medical institution

D. OMC imposes a heavy financial burden on patients

E. Difficult for the medical institution to construct a system and communication environment for OMC.

F. Difficult for patients to download applications and build a communication environment for OMC.

G. People concern about the content of the doctor's examination in OMC

H. Talk face-to-face is easier than that online

I. OMC requires patients to make a hospital visit when tests or procedures are needed

J. There are not many patients who are suitable for OMC.

K. People concern about the leakage of personal information through OMC

L. Because OMC itself is not well known

M. OMC are known, but people do not know when OMC are appropriate or desired.

N. OMC are known, but people do not know which institutions offer OMC.

O. Because the level of satisfaction with FMC is high and there are not many people who need OMC.

P. Regardless of the level of satisfaction with FMC, people tend to maintain the status quo.

12.

Please share any other thoughts you have about OMC, including what you find convenient, what you have trouble with, or what you hope to see in the future.

**Main questionnaire (4)**

1.

This questionnaire is for people who answered the following in the screening survey:

**- You or a family member are not currently visiting a doctor on a regular basis.**

**- You or a family member have no experience with online medical care (hereafter referred to as OMC).**

If the above condition does not apply to you, please stop answering at this time.

How many medical facilities are in your area? Please tell us your impression.

A. Numerous medical facilities nearby

B. A certain number of medical facilities nearby

C. Not many medical facilities nearby

2.

How do you feel about the time and effort when going to a doctor's office?

A. A lot of time and effort is needed when going to a doctor's office.

B. Some time and effort is needed when going to a doctor's office.

C. Not much time and effort is needed when going to a doctor's office.

D. No time and effort is needed when going to a doctor's office.

3.

For each of the following items, please select the one that best fits your choice.

|  | Acceptable with no problems at all | Resistance is present but acceptable. | Avoid if possible | Absolutely unacceptable |
| --- | --- | --- | --- | --- |
| About once a week in face-to-face consultations (hereafter referred to as FMC) |  |  |  |  |
| About once every two weeks in FMC |  |  |  |  |
| About once a month in FMC |  |  |  |  |
| About once a week visit in OMC |  |  |  |  |
| About once every two weeks visit in OMC |  |  |  |  |
| About once a month visit in OMC |  |  |  |  |

4.

Please tell us how you would rate the following items if you receive OMC compared to FMC.

Financial burden includes medical fees, transportation costs, connection fees, and other costs related to the visit.

|  | Obviously smaller in OMC | Slightly smaller in OMC | Roughly equivalent to FMC | Slightly larger in OMC | Obviously larger in OMC |
| --- | --- | --- | --- | --- | --- |
| time burden |  |  |  |  |  |
| physical burden |  |  |  |  |  |
| mental burden |  |  |  |  |  |
| financial burden |  |  |  |  |  |

5.

Please select which of the following departments you would be willing to use OMC.

A. Internal Medicine

B. Surgery

C. Pediatrics

D. Obstetrics and Gynecology/Reproductive Medicine

E. Psychiatry/Psychosomatic medicine

F. Dermatology

G. Ophthalmology

H. Otorhinolaryngology

I. Urology

J. Orthopedics

K. Plastic surgery/cosmetic surgery

L. None that apply

6.

In the following situations, please check the box you think it is acceptable to use OMC.

A. Explanation of test results

B. Regular medical checkup

C. Doing the same medication as usual

D. Consultation at the scene of a possible infectious disease such as a fever outpatient clinic

E. Consultation for sudden onset of illness (acute illness) other than infectious disease

F. When there is no doctor nearby who specializes in that disease

G. When it is difficult to go to a hospital (difficulty in transportation, living in a remote area, etc.)

H. Consultation on whether or not to see a doctor

I. Second opinion

J. Nutritional guidance

K. Opportunities other than general consultation (patient and doctor) such as, parent classes, conferences at the start of home medical care, etc.

L. None that apply

7.

In the following situations, please check the box if you would be willing to use OMC.

A. When a doctor or medical facility that has been providing FMC offers the option of OMC.

B. When the doctor you were receiving FMC has moved, and the option of OMC is offered in order to continue seeing him/her.

C. None that apply.

8.

Do you think it is feasible for you to receive OMC using your own device (computer, smartphone, tablet, etc.)?

A. I think I can do it alone, including preparation.

B. With help from family members, etc., I think it is feasible.

C. Difficult to do.

9.

Please select three factors from the following that you think are major impediments to the widespread use of OMC.

A. OMC increases administrative procedures on the part of medical institutions.

B. OMC requires more time and effort on the part of the patient.

C. OMC places a heavy financial burden on the medical institution

D. OMC imposes a heavy financial burden on patients

E. Difficult for the medical institution to construct a system and communication environment for OMC.

F. Difficult for patients to download applications and build a communication environment for OMC.

G. People concern about the content of the doctor's examination in OMC

H. Talk face-to-face is easier than that online

I. OMC requires patients to make a hospital visit when tests or procedures are needed

J. There are not many patients who are suitable for OMC.

K. People concern about the leakage of personal information through OMC

L. Because OMC itself is not well known

M. OMC are known, but people do not know when OMC are appropriate or desired.

N. OMC are known, but people do not know which institutions offer OMC.

O. Because the level of satisfaction with FMC is high and there are not many people who need OMC.

P. Regardless of the level of satisfaction with FMC, people tend to maintain the status quo.

10.

Please share any other thoughts you have about OMC, including what you find convenient, what you have trouble with, or what you hope to see in the future.
