# Supplementary material for: Issues in the Adoption of Online Medical Care: Cross-Sectional Questionnaire Survey
Source: J Med Internet Res. 2024 Nov 1;26:e64159. doi: 10.2196/64159 (PMC11568393; doi:10.2196/64159)
Supplement: Multimedia Appendix 3 [file jmir_v26i1e64159_app3.pdf]

Appendix 3. Original (Japanese) questionnaire for medical professionals

1. 回答者の職種を以下から選択ください。 なお、本調査の対象は、以下の区分にあてはまる方のみとなります。［ひとつだけ、回答必須]
1 つだけマークしてください。
◯医師
◯看護師（保健師・助産師含む）
◯経営者・事務担当者

2. ご回答されている方のイニシャルを、 名姓の順でご記載ください。[回答必須]
（例：病院 太郎→TB）

3. 電話番号をご記載ください。[任意]

4. E-mail アドレスをご記載ください。[任意]

5. 施設名をご記載ください。[回答必須]

6. 施設の標榜診療科をご選択ください。この際、当てはまるものは全て選択してください。[いくつでも、回答必須]
当てはまるものをすべて選択してください。
☐内科
☐外科
☐小児科
☐産婦人科・生殖医療
☐精神科
☐皮膚科
☐眼科
☐耳鼻咽喉科
☐泌尿器科
☐整形外科
☐形成外科・美容
☐人工透析
☐その他:

7. 医療機関の区分は下記のどれにあたりますか。［いくつでも、回答必須］
当てはまるものをすべて選択してください
□大学病院
□特定機能病院
□結核病院
□精神病院
□上記に当てはまらない病院
□診療所
□その他

8. 施設の位置する都道府県はどちらですか

ご回答にあたって 本アンケートにおいては、原則として、あなたが勤務する医療機関における現状についてご回答ください。例えば、あなたが属する診療科ではオンライン診療を行っていなかったとしても、他の診療科ではオンライン診療を行っている場合には、「医療機関としてはオンライン診療を行っている」ものとご回答ください。

9. 貴院では電子カルテシステムを使用されていますか。
[回答必須]* 1 つだけマークしてください。
○はい
○いいえ

※ 本設問の回答内容により 以降の設問は 3 パターンに分岐する。

10. 貴院ではオンライン診療は実施しておられますか。[回答必須]

オンライン診療は、“医師－患者間において、情報通信機器を通して、患者の診察及び診断を行い診断結果の伝達や処方等の診療行為を、リアルタイムにより行う行為”を指します。 1 つだけマークしてください。
○実施しており、自身もオンライン診療において患者さんと関わることがある
質問 11 にスキップします
○実施しているが、自身はオンライン診療において患者さんと関わることがない
質問 27 にスキップします
○実施していない
質問 41 にスキップします

「病院としてオンライン診療を実施しており、自身もオンライン診療で患者さんと関わることがある」と回答した方へのアンケートです。

パターン 1 の開始

11. 貴院ではどのような科がオンライン診療を利用していますか。知っている範囲でご回答ください。「その他」を選んだ方は、よろしければ詳細をご記載ください。
[いくつでも、回答必須] 当てはまるものをすべて選択してください。
□標榜している全ての科
□内科
□外科
□小児科
□産婦人科・生殖医療
□精神科・心療内科
□皮膚科
□眼科
□耳鼻咽喉科
□泌尿器科
□整形外科
□形成外科・美容外科
□その他:

12. 貴院ではどのような場面でオンライン診療を利用していますか。知っている範囲で ご回答ください。「その他」を選んだ方は、よろしければ詳細をご記載ください。
[いくつでも、回答必須] 当てはまるものをすべて選択してください。
□検査の結果説明
□定期的な診察
□いつもと同じ薬の処方
□発熱外来など、何らかの感染症が想定される場面での受診
□感染症以外の、急に発症した疾患（急性疾患）についての受診
□その疾患を専門とする医師が近くにいない場合の受診
□病院への通院が困難な場合（移動困難、遠隔地居住など）の受診
□受診するべきかどうかについての相談
□セカンドオピニオン
□栄養指導

□母親学級、遺伝相談、不妊相談や妊娠合併症の相談などの周産期に関する相談業務
□発達相談や子育て支援などの育児に関する相談業務
□在宅医療導入時カンファレンス
□詳細はわからない
□その他：

13. 貴院のオンライン診療システムについて、当てはまるものがあれば選んでください。[いくつでも、回答必須] 当てはまるものをすべて選択してください。
□医療機関側の操作が難しい
□電子カルテシステムと同じ端末では実施できない
□電子カルテシステムと同じ端末で実施できるが、ソフトウェアが統合されておらず使いづらい（電子カルテシステム内でのオンライン診療システムの稼働は不能、あるいはオンライン診 療システム内でのカルテ記載不能など）
□検査結果などの資料の画面共有ができない（カメラに写す必要がある）
□画面上で手書きでの図示ができない（手元の紙に書き、カメラに写す必要がある）
□通信環境の問題でトラブルが起こったことがある
□患者側の操作が難しい
□当てはまるものはない

14. 対面診療と比べオンライン診療では、医師に対するコメディカルの補助が必要なこ とが多いですか、少ないですか。[ひとつだけ、回答必須]
※医師に対するコメディカルの補助とは、 看護師や事務担当者による事前問診、カルテ操作補助、オンライン診療に関連する操作補助等の、診療補助業務を指します。
1 つだけマークしてください。
〇オンライン診療の方が、明らかに多い
〇オンライン診療の方が、やや多い
〇対面診療とオンライン診療とでは、おおむね同等である
〇オンライン診療の方が、やや少ない
〇オンライン診療の方が、明らかに少ない

15. オンライン診療を行った場合に必要となる病院側の業務は、対面診療を行った場合 より複雑ですか、簡単ですか。[ひとつだけ、回答必須]
※　病院側の業務とは、患者への各種案内業務、会計業務、その他事務的手続きなど を指します。
1 つだけマークしてください。
〇オンライン診療の方が、明らかに複雑である

〇オンライン診療の方が、やや複雑である
〇オンライン診療と対面診療とで同等である
〇オンライン診療の方が、やや簡単である
〇オンライン診療の方が、明らかに簡単である

16. 対面診療と比較してオンライン診療では、単位時間あたりに診療可能な患者数は多くなりますか、少なくなりますか。[ひとつだけ、回答必須]
1 つだけマークしてください。
〇オンライン診療の方が、多くなる
〇対面診療とオンライン診療とで、ほぼ同数である
〇オンライン診療の方が、少なくなる

17. オンライン診療での患者の負担は、対面診療と比べどの程度だと推定されますか。各項目について、5 つの選択肢のうち最も当てはまるものを選んでください。
[各項目につきひとつだけ、回答必須]
※金銭的負担には、診療費、交通費、回線料金など受診に関連した諸々を含みます
1 行につき 1 つだけマークしてください。

	オンライン診療の方が明らかに小さい	オンライン診療の方がやや小さい	対面診療と概ね同等である	対面診療の方がやや小さい	対面診療の方が明らかに小さい
時間的負担	☐	☐	☐	☐	☐
身体的負担	☐	☐	☐	☐	☐
精神的負担	☐	☐	☐	☐	☐
金銭的負担	☐	☐	☐	☐	☐

18.オンライン診療と対面診療とを比較した場合の、以下の 7 つの診療プロセスの容易さについ て、選択肢のうち最も当てはまると感じるものを選んでください。

18-1. 患者の病状の重症感を直感的に把握することができる。[ひとつだけ、回答必須]
1 つだけマークしてください。
〇オンライン診療の方が明らかに容易である
〇オンライン診療の方がやや容易である
〇対面診療とオンライン診療で概ね同等である

◯対面診療の方がやや容易である
◯対面診療の方が明らかに容易である

18-2. 患者の身体的所見を把握することができる。[ひとつだけ、回答必須]
1 つだけマークしてください。
◯オンライン診療の方が明らかに容易である
◯オンライン診療の方がやや容易である
◯対面診療とオンライン診療で概ね同等である
◯対面診療の方がやや容易である
◯対面診療の方が明らかに容易である

18-3. 患者と言語的コミュニケーションをとることができる。
[ひとつだけ、回答必須]
1 つだけマークしてください。
◯オンライン診療の方が明らかに容易である
◯オンライン診療の方がやや容易である
◯対面診療とオンライン診療で概ね同等である
◯対面診療の方がやや容易である
◯対面診療の方が明らかに容易である

18-4. 家族から患者本人の病状・行動に関する話を聞くことができる。
[ひとつだけ、回答必須]
1 つだけマークしてください。
◯オンライン診療の方が明らかに容易である
◯オンライン診療の方がやや容易である
◯対面診療とオンライン診療で概ね同等である
◯対面診療の方がやや容易である
◯対面診療の方が明らかに容易である

18-5. 自宅などにおける患者本人の病状・行動などを視覚的に把握できる。
[ひとつだけ、回答必須]
1 つだけマークしてください。
◯オンライン診療の方が明らかに容易である
◯オンライン診療の方がやや容易である
◯対面診療とオンライン診療で概ね同等である
◯対面診療の方がやや容易である

〇対面診療の方が明らかに容易である

18-6. 患者本人が日常生活を送る自宅などの環境を視覚的に把握できる。

［ひとつだけ、回答必須］

1 つだけマークしてください。

〇オンライン診療の方が明らかに容易である

〇オンライン診療の方がやや容易である

〇対面診療とオンライン診療で概ね同等である

〇対面診療の方がやや容易である

〇対面診療の方が明らかに容易である

18-7. 患者と医師の間の信頼関係を作ることができる。［ひとつだけ、回答必須］

1 つだけマークしてください。

〇オンライン診療の方が明らかに容易である

〇オンライン診療の方がやや容易である

〇対面診療とオンライン診療で概ね同等である

〇対面診療の方がやや容易である

〇対面診療の方が明らかに容易である

19. オンライン診療を実施するためのシステムの導入・維持費用は高額だと感じます か。

［ひとつだけ、回答必須］

1 つだけマークしてください。

〇明らかに高額である

〇やや高額である

〇おおむね妥当である

〇やや安価である

〇明らかに安価である

〇わからない

20. オンライン診療に対する診療報酬を知っていますか。 対面診療の場合の診療報酬より低い／あるいは高い、同等であるなどの、おおまかな知識で構いません。［ひとつだけ、回答必須］

1 つだけマークしてください。

〇はい

〇いいえ

21. 対面診療およびオンライン診療における診療報酬は、現在以下に示した通りで す。
オンライン診療における診療報酬を高いと感じますか、低いと感じますか。［ひとつだけ、回答必須]

オンライン診療　初診料 **251 点**　※施設基準を満たし届出を行った機関の場合

オンライン診療　再診料 **73 点**

対面診療　初診料 **288 点**

対面診療　再診料 **73 点**（診療所と一般病床 200 床未満の病院の場合）

74 点（一般病床が 200 床以上の病院の場合）

なお、医学管理料（特定疾患療養管理料、難病外来指導管理料など）は、オンライン診療では対面診療における保険点数の約 **87%** に設定されている。

1 つだけマークしてください。

〇診療報酬は、明らかに低い

〇診療報酬は、やや低い

〇診療報酬は、妥当である

〇診療報酬は、やや高い

〇診療報酬は、明らかに高い

〇わからない

22. オンライン診療によって利益を得る患者は、貴院に通院している患者のうち何 パーセント程度を占めると思いますか。
最も当てはまるものをご選択ください。[ひとつだけ、回答必須]

1 つだけマークしてください。

〇0%

〇10%

〇20%

〇30%

〇40%

〇50%

〇60%

〇70%

〇80%

〇90%

〇100%

23. 対象患者がおおよそ何歳以下であれば、自身の端末（パソコン、スマートフォ ン、

タブレット等）を用いたオンライン診療を一人で実行可能だと思いますか。
［ひとつだけ、回答必須]
1 つだけマークしてください。
◯50 歳
◯55 歳
◯60 歳
◯65 歳
◯70 歳
◯75 歳
◯80 歳
◯85 歳
◯年齢は関係ないと考える

24.以下の 10 の診療行為を行う場合、対面診療とオンライン診療のどちらの方がより適しているか、あなたのご意見に最も近いものを選んでください。

24-1. 検査の結果説明 [ひとつだけ、必須回答]
1 つだけマークしてください。
◯オンライン診療の方が明らかに適している
◯オンライン診療の方がやや適している
◯対面診療とオンライン診療で概ね同等である
◯対面診療の方がやや適している
◯対面診療の方が明らかに適している

24-2. 定期的な診察 [ひとつだけ、回答必須]
1 つだけマークしてください。
◯オンライン診療の方が明らかに適している
◯オンライン診療の方がやや適している
◯対面診療とオンライン診療で概ね同等である
◯対面診療の方がやや適している
◯対面診療の方が明らかに適している

24-3. いつもと同じ薬の処方 [ひとつだけ、回答必須]
1 つだけマークしてください。
◯オンライン診療の方が明らかに適している
◯オンライン診療の方がやや適している

〇対面診療とオンライン診療で概ね同等である
〇対面診療の方がやや適している
〇対面診療の方が明らかに適している。

24-4. 発熱外来など、何らかの感染症が想定される場面での受診
[ひとつだけ、回答必須]
1 つだけマークしてください。
〇オンライン診療の方が明らかに適している
〇オンライン診療の方がやや適している
〇対面診療とオンライン診療で概ね同等である
〇対面診療の方がやや適している
〇対面診療の方が明らかに適している。

24-5. その疾患を専門とする医師が近くにいない場合の受診
[ひとつだけ、回答必須]
1 つだけマークしてください。
〇オンライン診療の方が明らかに適している
〇オンライン診療の方がやや適している
〇対面診療とオンライン診療で概ね同等である
〇対面診療の方がやや適している
〇対面診療の方が明らかに適している。

24-6. 病院への通院が困難な場合（移動困難、遠隔地居住など）の受診
[ひとつだけ、回答必須]
1 つだけマークしてください。
〇オンライン診療の方が明らかに適している
〇オンライン診療の方がやや適している
〇対面診療とオンライン診療で概ね同等である
〇対面診療の方がやや適している
〇対面診療の方が明らかに適している。

24-7. セカンドオピニオン　[ひとつだけ、回答必須]
1 つだけマークしてください。
〇オンライン診療の方が明らかに適している
〇オンライン診療の方がやや適している
〇対面診療とオンライン診療で概ね同等である

〇対面診療の方がやや適している
〇対面診療の方が明らかに適している。

24-8. 栄養指導 ［ひとつだけ、回答必須］
1 つだけマークしてください。
〇オンライン診療の方が明らかに適している
〇オンライン診療の方がやや適している
〇対面診療とオンライン診療で概ね同等である
〇対面診療の方がやや適している
〇対面診療の方が明らかに適している。

24-9. 母親学級、遺伝相談、不妊相談や妊娠合併症の相談などの周産期に関する相談業務 ［ひとつだけ、回答必須］
1 つだけマークしてください。
〇オンライン診療の方が明らかに適している
〇オンライン診療の方がやや適している
〇対面診療とオンライン診療で概ね同等である
〇対面診療の方がやや適している
〇対面診療の方が明らかに適している。

24-10. 発達支援や子育て支援などの育児に関する相談業務 ［ひとつだけ、回答必須］
1 つだけマークしてください。
〇オンライン診療の方が明らかに適している
〇オンライン診療の方がやや適している
〇対面診療とオンライン診療で概ね同等である
〇対面診療の方がやや適している
〇対面診療の方が明らかに適している。

25. 以下のうち、オンライン診療の普及を阻害する要因として当てはまるものと考えるものを、全て（少なくとも一つ以上）選択してください。
［いくつでも、回答必須］当てはまるものをすべて選択してください。
☐オンライン診療では、医療機関側の事務的な手続きが増えてしまうため
☐オンライン診療では、医師側の手間が増えてしまうため
☐オンライン診療では、患者側の手間が増えてしまうため
☐オンライン診療では、医療機関側の金銭的負担が大きいため
☐オンライン診療では、患者の金銭的負担が大きいため

□オンライン診療のためのシステムや通信環境の構築が、医療機関にとって困難であるため

□オンライン診療のためのアプリダウンロード・通信環境の構築などが、患者にとって困難であるため

□オンライン診療では、医師の診察内容に不安が残るため

□オンラインよりも、対面の方が話しやすいため

□オンライン診療では、検査や処置が必要になった場合に結局通院が必要になってしまうため

□オンライン診療に適した患者があまりいないため

□オンライン診療による個人情報漏洩が心配であるため

□医学部生や若手の教育のためにはオンライン診療よりも対面診療の方が望ましいため

□オンライン診療という診療形態自体があまり知られていないため

□オンライン診療という実施形態は知られているが、どういった場合にオンライン診療が適し ているのか/希望してよいのかわからないため

□オンライン診療という実施形態は知られているが、オンライン診療を実施している機関がどこかわからないため

□対面診療の満足度が高く、オンライン診療を必要としている人が少ないため。

□対面診療の満足度に関わらず、現状維持を望む心理が働くため

26. オンライン診療について、便利である点、困っている点、今後期待する点など、何かお考えがあればお教えください。

「病院としてはオンライン診療を実施しているが、自身はオンライン診療で患者さんと関わることがない」と回答した方への質問です。
パターン 2 の開始

27. 貴院ではどのような科がオンライン診療を利用していますか。知っている範囲でご 回答ください。「その他」を選んだ方は、よろしければ詳細をご記載ください。
[いくつでも、回答必須] 当てはまるものをすべて選択してください。

□標榜している全ての科
□内科
□外科
□小児科
□産婦人科・生殖医療
□精神科・心療内科
□皮膚科
□眼科
□耳鼻咽喉科
□泌尿器科
□整形外科
□形成外科・美容外科
□その他:

28. 貴院ではどのような場面でオンライン診療を利用していますか。知っている範囲で ご回答ください。「その他」を選んだ方は、よろしければ詳細をご記載ください。
[いくつでも、回答必須] 当てはまるものをすべて選択してください。

□検査の結果説明
□定期的な診察
□いつもと同じ薬の処方
□発熱外来など、何らかの感染症が想定される場面での受診
□感染症以外の、急に発症した疾患（急性疾患）についての受診
□その疾患を専門とする医師が近くにいない場合の受診
□病院への通院が困難な場合（移動困難、遠隔地居住など）の受診
□受診するべきかどうかについての相談
□セカンドオピニオン

□栄養指導
□母親学級、遺伝相談、不妊相談や妊娠合併症の相談などの周産期にに関する相談業務
□発達相談や子育て支援などの育児に関する相談業務
□在宅医療導入時カンファレンス
□詳細はわからない
□その他：

29. 【医師の方にお伺いいたします】 貴院でオンライン診療は実施されているが自身 は携わっていない場合、その理由として当てはまるものはどれですか。
「その他」を選んだ方は、よろしければ詳細をご記載ください。
[いくつでも、回答 必須]
※医師でない場合、「本設問の回答対象外である」をご選択ください。

□本設問の回答対象外である
□当院でオンライン診療を実施しているのは、他診療科のみである
□当院でオンライン診療を実施しているのは、セカンドオピニオンなど限られた場面のみである
□オンライン診療を行う方が対面診療よりも手間がかかる
□オンライン診療を行うためには事前に研修を受講するなどといった準備が必要になる
□自身が診療している分野はオンライン診療に適していないと考えている
□自身が診療している患者にオンライン診療に適した患者がいない
□学生や若手医師の教育のために、対面診療を選択している
□オンライン診療の選択肢を提示しても、患者の承諾が得られない
□対面診療で十分であり、オンライン診療をするモチベーションがない
□その他：

30. 対面診療と比較して、オンライン診療では、単位時間あたりに診療可能な患者数は多くなりますか、少なくなりますか。
[ひとつだけ、回答必須]
1 つだけマークしてください。
〇オンライン診療の方が、多くなる
〇対面診療とオンライン診療とで、ほぼ同数である
〇オンライン診療の方が、少なくなる

31. オンライン診療での患者の負担は、対面診療と比べてどの程度だと推定されますか。各項目について、5 つの選択肢のうち最も当てはまるものを選んでください。

[各項目につきひとつだけ、回答必須]

※金銭的負担には、診療費、交通費、回線料金など受診に関連した諸々を含みます。

	オンライン診療の方が明らかに小さい	オンライン診療の方がやや小さい	対面診療と概ね同等である	対面診療の方がやや小さい	対面診療の方が明らかに小さい
時間的負担	☐	☐	☐	☐	☐
身体的負担	☐	☐	☐	☐	☐
精神的負担	☐	☐	☐	☐	☐
金銭的負担	☐	☐	☐	☐	☐

32. オンライン診療と対面診療とを比較した場合の、以下の 7 つの診療プロセスの容易さについて、選択肢のうち最もあてはまると感じるものを選んでください。

32-1. 患者の病状の重症感を直感的に把握することができる。[ひとつだけ、回答必須]

1 つだけマークしてください。

◯オンライン診療の方が明らかに容易である

◯オンライン診療の方がやや容易である

◯対面診療とオンライン診療で概ね同等である

◯対面診療の方がやや容易である

◯対面診療の方が明らかに容易である

32-2. 患者の身体的所見を把握することができる。[ひとつだけ、回答必須]

1 つだけマークしてください。

◯オンライン診療の方が明らかに容易である

◯オンライン診療の方がやや容易である

◯対面診療とオンライン診療で概ね同等である

◯対面診療の方がやや容易である

◯対面診療の方が明らかに容易である

32-3. 患者と言語的コミュニケーションをとることができる。

[ひとつだけ、回答必須]

1 つだけマークしてください。

〇オンライン診療の方が明らかに容易である
〇オンライン診療の方がやや容易である
〇対面診療とオンライン診療で概ね同等である
〇対面診療の方がやや容易である
〇対面診療の方が明らかに容易である

32-4. 家族から患者本人の病状・行動に関する話を聞くことができる。
［ひとつだけ、回答必須］
1 つだけマークしてください。
〇オンライン診療の方が明らかに容易である
〇オンライン診療の方がやや容易である
〇対面診療とオンライン診療で概ね同等である
〇対面診療の方がやや容易である
〇対面診療の方が明らかに容易である

32-5. 自宅などにおける患者本人の病状・行動などを視覚的に把握できる。
［ひとつだけ、回答必須］
1 つだけマークしてください。
〇オンライン診療の方が明らかに容易である
〇オンライン診療の方がやや容易である
〇対面診療とオンライン診療で概ね同等である
〇対面診療の方がやや容易である
〇対面診療の方が明らかに容易である

32-6. 患者本人が日常生活を送る自宅などの環境を視覚的に把握できる。
［ひとつだけ、回答必須］
1 つだけマークしてください。
〇オンライン診療の方が明らかに容易である
〇オンライン診療の方がやや容易である
〇対面診療とオンライン診療で概ね同等である
〇対面診療の方がやや容易である
〇対面診療の方が明らかに容易である

32-7. 患者と医師の間の信頼関係を作ることができる。［ひとつだけ、回答必須］
1 つだけマークしてください。
〇オンライン診療の方が明らかに容易である

〇オンライン診療の方がやや容易である
〇対面診療とオンライン診療で概ね同等である
〇対面診療の方がやや容易である
〇対面診療の方が明らかに容易である

33. オンライン診療を実施するためのシステムの導入・維持費用は高額だと感じます か。
[ひとつだけ、回答必須]
1 つだけマークしてください。
〇明らかに高額である
〇やや高額である
〇おおむね妥当である
〇やや安価である
〇明らかに安価である
〇わからない

34. オンライン診療に対する診療報酬を知っていますか。 対面診療の場合の診療報酬より低い／あるいは高い、同等であるなどの、おおまかな知識で構いません。[ひとつだけ、回答必須]
1 つだけマークしてください。
〇はい
〇いいえ

35. 対面診療およびオンライン診療における診療報酬は、現在以下に示した通りで す。オンライン診療における診療報酬を高いと感じますか、低いと感じますか。[ひとつだけ、回答必須]
オンライン診療　初診料 **251 点**　※施設基準を満たし届出を行った機関の場合
オンライン診療　再診料 **73 点**
対面診療　初診料 **288 点**
対面診療　再診料 **73 点**（診療所と一般病床 200 床未満の病院の場合）
74 点（一般病床が 200 床以上の病院の場合）
なお、医学管理料（特定疾患療養管理料、難病外来指導管理料など）は、オンライン診療では対面診療における保険点数の約 **87%**に設定されている。

1 つだけマークしてください。
〇診療報酬は、明らかに低い
〇診療報酬は、やや低い

〇診療報酬は、妥当である
〇診療報酬は、やや高い
〇診療報酬は、明らかに高い
〇わからない

36. オンライン診療によって利益を得る患者は、貴院に通院している患者のうちどの程度の割合を占めると思いますか。[ひとつだけ、回答必須]

1 つだけマークしてください。

〇0%
〇10%
〇20%
〇30%
〇40%
〇50%
〇60%
〇70%
〇80%
〇90%
〇100%

37. 対象患者がおおよそ何歳以下であれば、自身の端末（パソコン、スマートフォ ン、タブレット等）を用いたオンライン診療を一人で実行可能だと思いますか。
[ひとつだけ、回答必須]

1 つだけマークしてください。

〇50 歳
〇55 歳
〇60 歳
〇65 歳
〇70 歳
〇75 歳
〇80 歳
〇85 歳
〇年齢は関係ないと考え

38. 以下の 10 の診療行為を行う場合、対面診療とオンライン診療のどちらの方がより適しているか、あなたのご意見に最も近いものを選んでください。

38-1. 検査の結果説明　[ひとつだけ、必須回答]

1 つだけマークしてください。

◯オンライン診療の方が明らかに適している

◯オンライン診療の方がやや適している

◯対面診療とオンライン診療で概ね同等である

◯対面診療の方がやや適している

◯対面診療の方が明らかに適している

38-2. 定期的な診察　[ひとつだけ、回答必須]

1 つだけマークしてください。

◯オンライン診療の方が明らかに適している

◯オンライン診療の方がやや適している

◯対面診療とオンライン診療で概ね同等である

◯対面診療の方がやや適している

◯対面診療の方が明らかに適している

38-3. いつもと同じ薬の処方　[ひとつだけ、回答必須]

1 つだけマークしてください。

◯オンライン診療の方が明らかに適している

◯オンライン診療の方がやや適している

◯対面診療とオンライン診療で概ね同等である

◯対面診療の方がやや適している

◯対面診療の方が明らかに適している。

38-4. 発熱外来など、何らかの感染症が想定される場面での受診

[ひとつだけ、回答必須]

1 つだけマークしてください。

◯オンライン診療の方が明らかに適している

◯オンライン診療の方がやや適している

◯対面診療とオンライン診療で概ね同等である

◯対面診療の方がやや適している

◯対面診療の方が明らかに適している。

38-5. その疾患を専門とする医師が近くにいない場合の受診

[ひとつだけ、回答必須]

1 つだけマークしてください。
◯オンライン診療の方が明らかに適している
◯オンライン診療の方がやや適している
◯対面診療とオンライン診療で概ね同等である
◯対面診療の方がやや適している
◯対面診療の方が明らかに適している。

38-6. 病院への通院が困難な場合（移動困難、遠隔地居住など）の受診
[ひとつだけ、回答必須]
1 つだけマークしてください。
◯オンライン診療の方が明らかに適している
◯オンライン診療の方がやや適している
◯対面診療とオンライン診療で概ね同等である
◯対面診療の方がやや適している
◯対面診療の方が明らかに適している。

38-7. セカンドオピニオン　[ひとつだけ、回答必須]
1 つだけマークしてください。
◯オンライン診療の方が明らかに適している
◯オンライン診療の方がやや適している
◯対面診療とオンライン診療で概ね同等である
◯対面診療の方がやや適している
◯対面診療の方が明らかに適している。

38-8. 栄養指導　[ひとつだけ、回答必須]
1 つだけマークしてください。
◯オンライン診療の方が明らかに適している
◯オンライン診療の方がやや適している
◯対面診療とオンライン診療で概ね同等である
◯対面診療の方がやや適している
◯対面診療の方が明らかに適している。

38-9. 母親学級、遺伝相談、不妊相談や妊娠合併症の相談などの周産期に関する相談業務　[ひとつだけ、回答必須]
1 つだけマークしてください。
◯オンライン診療の方が明らかに適している

〇オンライン診療の方がやや適している
〇対面診療とオンライン診療で概ね同等である
〇対面診療の方がやや適している
〇対面診療の方が明らかに適している。

38-10. 発達支援や子育て支援などの育児に関する相談業務 ［ひとつだけ、回答必須］

1 つだけマークしてください。

〇オンライン診療の方が明らかに適している
〇オンライン診療の方がやや適している
〇対面診療とオンライン診療で概ね同等である
〇対面診療の方がやや適している
〇対面診療の方が明らかに適している。

39. 以下のうち、オンライン診療の普及を阻害する要因として当てはまるものと考えるものを、全て（少なくとも一つ以上）選択してください。

[いくつでも、回答必須] 当てはまるものをすべて選択してください。

□オンライン診療では、医療機関側の事務的な手続きが増えてしまうため
□オンライン診療では、医師側の手間が増えてしまうため
□オンライン診療では、患者側の手間が増えてしまうため
□オンライン診療では、医療機関側の金銭的負担が大きいため
□オンライン診療では、患者の金銭的負担が大きいため
□オンライン診療のためのシステムや通信環境の構築が、医療機関にとって困難であるため
□オンライン診療のためのアプリダウンロード・通信環境の構築などが、患者にとって困難であるため
□オンライン診療では、医師の診察内容に不安が残るため
□オンラインよりも、対面の方が話しやすいため
□オンライン診療では、検査や処置が必要になった場合に結局通院が必要になってしまうため
□オンライン診療に適した患者があまりいないため
□オンライン診療による個人情報漏洩が心配であるため
□医学部生や若手の教育のためにはオンライン診療よりも対面診療の方が望ましいため
□オンライン診療という診療形態自体があまり知られていないため
□オンライン診療という実施形態は知られているが、どういった場合にオンライン診療が適し ているのか/希望してよいのかわからないため

□オンライン診療という実施形態は知られているが、オンライン診療を実施している機関がどこかわからないため
□対面診療の満足度が高く、オンライン診療を必要としている人が少ないため。
□対面診療の満足度に関わらず、現状維持を望む心理が働くため

40. オンライン診療について、便利である点、困っている点、今後期待する点など、何かお考えがあればお教えください。

「オンライン診療を実施していない」と回答した方への質問です。
パターン 3 の開始

41. オンライン診療での患者の負担は、対面診療と比べてどの程度だと推定されますか。各項目について、5 つの選択肢のうち最も当てはまると思うものを選んでください。
[各項目につきひとつだけ、回答必須]
※金銭的負担には、診療費、交通費、回線料金など受診に関連した諸々を含みます。

	オンライン診療の方が明らかに小さい	オンライン診療の方がやや小さい	対面診療と概ね同等である	対面診療の方がやや小さい	対面診療の方が明らかに小さい
時間的負担	☐	☐	☐	☐	☐
身体的負担	☐	☐	☐	☐	☐
精神的負担	☐	☐	☐	☐	☐
金銭的負担	☐	☐	☐	☐	☐

42. オンライン診療と対面診療とを比較した場合の、以下の各診療プロセスの容易さについて、選択肢のうち最もあてはまると感じるものを選んでください。

42-1. 患者の病状の重症感を直感的に把握することができる。[ひとつだけ、回答必須]
1 つだけマークしてください。
〇オンライン診療の方が明らかに容易である
〇オンライン診療の方がやや容易である
〇対面診療とオンライン診療で概ね同等である
〇対面診療の方がやや容易である
〇対面診療の方が明らかに容易である

42-2. 患者の身体的所見を把握することができる。[ひとつだけ、回答必須]
1 つだけマークしてください。
〇オンライン診療の方が明らかに容易である
〇オンライン診療の方がやや容易である
〇対面診療とオンライン診療で概ね同等である
〇対面診療の方がやや容易である
〇対面診療の方が明らかに容易である

42-3. 患者と言語的コミュニケーションをとることができる。

［ひとつだけ、回答必須］

1 つだけマークしてください。

◯オンライン診療の方が明らかに容易である

◯オンライン診療の方がやや容易である

◯対面診療とオンライン診療で概ね同等である

◯対面診療の方がやや容易である

◯対面診療の方が明らかに容易である

42-4. 家族から患者本人の病状・行動に関する話を聞くことができる。

［ひとつだけ、回答必須］

1 つだけマークしてください。

◯オンライン診療の方が明らかに容易である

◯オンライン診療の方がやや容易である

◯対面診療とオンライン診療で概ね同等である

◯対面診療の方がやや容易である

◯対面診療の方が明らかに容易である

42-5. 自宅などにおける患者本人の病状・行動などを視覚的に把握できる。

［ひとつだけ、回答必須］

1 つだけマークしてください。

◯オンライン診療の方が明らかに容易である

◯オンライン診療の方がやや容易である

◯対面診療とオンライン診療で概ね同等である

◯対面診療の方がやや容易である

◯対面診療の方が明らかに容易である

42-6. 患者本人が日常生活を送る自宅などの環境を視覚的に把握できる。

［ひとつだけ、回答必須］

1 つだけマークしてください。

◯オンライン診療の方が明らかに容易である

◯オンライン診療の方がやや容易である

◯対面診療とオンライン診療で概ね同等である

◯対面診療の方がやや容易である

◯対面診療の方が明らかに容易である

42-7. 患者と医師の間の信頼関係を作ることができる。[ひとつだけ、回答必須]

1 つだけマークしてください。

〇オンライン診療の方が明らかに容易である

〇オンライン診療の方がやや容易である

〇対面診療とオンライン診療で概ね同等である

〇対面診療の方がやや容易である

〇対面診療の方が明らかに容易である

43. オンライン診療を実施するためのシステムの導入・維持費用は高額だと感じますか。[ひとつだけ、回答必須]

1 つだけマークしてください。

〇明らかに高額である

〇やや高額である

〇おおむね妥当である

〇やや安価である

〇明らかに安価である

〇わからない

44. オンライン診療に対する診療報酬を知っていますか。 対面診療の場合の診療報酬より低い／あるいは高い、同等であるなどの、おおまかな 知識で構いません。[ひとつだけ、回答必須]

1 つだけマークしてください。

〇はい

〇いいえ

45 対面診療およびオンライン診療における診療報酬は、現在以下に示した通りで す。オンライン診療における診療報酬を高いと感じますか、低いと感じますか。 [ひとつだけ、回答必須]

オンライン診療　初診料 **251 点**　※施設基準を満たし届出を行った機関の場合

オンライン診療　再診料 **73 点**

対面診療　初診料 **288 点**

対面診療　再診料 **73 点**（診療所と一般病床 200 床未満の病院の場合）

74 点（一般病床が 200 床以上の病院の場合）

なお、医学管理料（特定疾患療養管理料、難病外来指導管理料など）は、オンライン診療では対面診療における保険点数の約 **87%**に設定されている。

1 つだけマークしてください。

〇診療報酬は、明らかに低い

〇診療報酬は、やや低い

〇診療報酬は、妥当である

〇診療報酬は、やや高い

〇診療報酬は、明らかに高い

〇わからない

46. オンライン診療によって利益を得る患者は、貴院に通院している患者のうち何パーセント程度を占めると思いますか。[ひとつだけ、回答必須]

1 つだけマークしてください。

〇0%

〇10%

〇20%

〇30%

〇40%

〇50%

〇60%

〇70%

〇80%

〇90%

〇100%

47. 対象患者がおおよそ何歳以下であれば、自身の端末（パソコン、スマートフォン、タブレット等）を用いたオンライン診療を一人で実行可能だと思いますか。

[ひとつだけ、回答必須]

1 つだけマークしてください。

〇50 歳

〇55 歳

〇60 歳

〇65 歳

〇70 歳

〇75 歳

〇80 歳

〇85 歳

〇年齢は関係ないと考え

48. 以下の 10 の診療行為を行う場合、対面診療とオンライン診療のどちらの方がより適しているか、あなたのご意見に最も近いものを選んでください。

48-1. 検査の結果説明 [ひとつだけ、必須回答]

1 つだけマークしてください。

〇オンライン診療の方が明らかに適している
〇オンライン診療の方がやや適している
〇対面診療とオンライン診療で概ね同等である
〇対面診療の方がやや適している
〇対面診療の方が明らかに適している

48-2. 定期的な診察 [ひとつだけ、回答必須]

1 つだけマークしてください。

〇オンライン診療の方が明らかに適している
〇オンライン診療の方がやや適している
〇対面診療とオンライン診療で概ね同等である
〇対面診療の方がやや適している
〇対面診療の方が明らかに適している

48-3. いつもと同じ薬の処方 [ひとつだけ、回答必須]

1 つだけマークしてください。

〇オンライン診療の方が明らかに適している
〇オンライン診療の方がやや適している
〇対面診療とオンライン診療で概ね同等である
〇対面診療の方がやや適している
〇対面診療の方が明らかに適している。

48-4. 発熱外来など、何らかの感染症が想定される場面での受診

[ひとつだけ、回答必須]

1 つだけマークしてください。

〇オンライン診療の方が明らかに適している
〇オンライン診療の方がやや適している
〇対面診療とオンライン診療で概ね同等である
〇対面診療の方がやや適している
〇対面診療の方が明らかに適している。

48-5. その疾患を専門とする医師が近くにいない場合の受診

［ひとつだけ、回答必須］

1 つだけマークしてください。

◯オンライン診療の方が明らかに適している

◯オンライン診療の方がやや適している

◯対面診療とオンライン診療で概ね同等である

◯対面診療の方がやや適している

◯対面診療の方が明らかに適している。

48-6. 病院への通院が困難な場合（移動困難、遠隔地居住など）の受診

［ひとつだけ、回答必須］

1 つだけマークしてください。

◯オンライン診療の方が明らかに適している

◯オンライン診療の方がやや適している

◯対面診療とオンライン診療で概ね同等である

◯対面診療の方がやや適している

◯対面診療の方が明らかに適している。

48-7. セカンドオピニオン　［ひとつだけ、回答必須］

1 つだけマークしてください。

◯オンライン診療の方が明らかに適している

◯オンライン診療の方がやや適している

◯対面診療とオンライン診療で概ね同等である

◯対面診療の方がやや適している

◯対面診療の方が明らかに適している。

48-8. 栄養指導　［ひとつだけ、回答必須］

1 つだけマークしてください。

◯オンライン診療の方が明らかに適している

◯オンライン診療の方がやや適している

◯対面診療とオンライン診療で概ね同等である

◯対面診療の方がやや適している

◯対面診療の方が明らかに適している。

48-9. 母親学級、遺伝相談、不妊相談や妊娠合併症の相談などの周産期に関する相談

業務 ［ひとつだけ、回答必須］

1 つだけマークしてください。

◯オンライン診療の方が明らかに適している

◯オンライン診療の方がやや適している

◯対面診療とオンライン診療で概ね同等である

◯対面診療の方がやや適している

◯対面診療の方が明らかに適している。

48-10. 発達支援や子育て支援などの育児に関する相談業務 ［ひとつだけ、回答必須］

1 つだけマークしてください。

◯オンライン診療の方が明らかに適している

◯オンライン診療の方がやや適している

◯対面診療とオンライン診療で概ね同等である

◯対面診療の方がやや適している

◯対面診療の方が明らかに適している。

49. 以下のうち、オンライン診療の普及を阻害する要因として当てはまるものと考えるものを、全て（少なくとも一つ以上）選択してください。

［いくつでも、回答必須］当てはまるものをすべて選択してください。

☐オンライン診療では、医療機関側の事務的な手続きが増えてしまうため

☐オンライン診療では、医師側の手間が増えてしまうため

☐オンライン診療では、患者側の手間が増えてしまうため

☐オンライン診療では、医療機関側の金銭的負担が大きいため

☐オンライン診療では、患者の金銭的負担が大きいため

☐オンライン診療のためのシステムや通信環境の構築が、医療機関にとって困難であるため

☐オンライン診療のためのアプリダウンロード・通信環境の構築などが、患者にとって困難で あるため

☐オンライン診療では、医師の診察内容に不安が残るため

☐オンラインよりも、対面の方が話しやすいため

☐オンライン診療では、検査や処置が必要になった場合に結局通院が必要になってしまうため

☐オンライン診療に適した患者があまりいないため

☐オンライン診療による個人情報漏洩が心配であるため

☐医学部生や若手の教育のためにはオンライン診療よりも対面診療の方が望ましいため

□オンライン診療という診療形態自体があまり知られていないため
□オンライン診療という実施形態は知られているが、どういった場合にオンライン診療が適し ているのか/希望してよいのかわからないため
□オンライン診療という実施形態は知られているが、オンライン診療を実施している機関がどこかわからないため
□対面診療の満足度が高く、オンライン診療を必要としている人が少ないため。
□対面診療の満足度に関わらず、現状維持を望む心理が働くため

50. オンライン診療について、便利である点、困っている点、今後期待する点など、何かお考えがあればお教えください。
