# Supplementary material for: Issues in the Adoption of Online Medical Care: Cross-Sectional Questionnaire Survey
Source: J Med Internet Res. 2024 Nov 1;26:e64159. doi: 10.2196/64159 (PMC11568393; doi:10.2196/64159)
Supplement: Multimedia Appendix 4 [file jmir_v26i1e64159_app4.doc]

**Appendix 4. English-translated questionnaire for medical professionals**

1. Please select the job title of the respondent from the following options. Please note that this survey is only applicable to those who fit into the following categories.

A. Doctor

B. Nurse (including public health nurses and midwives)

C. Management/administrative staff

2. Please indicate the initials of the respondent, in order of first name and surname.

3. Please state your telephone number. [arbitrary]

4. Please state your E-mail address. [arbitrary]

5. Please state the name of the facility.

6. Please select all the affiliated medical departments of your facility.

A. Internal Medicine

B. Surgery

C. Pediatrics

D. Obstetrics & Gynecology

E. Psychiatry

F. Dermatology

G. Ophthalmology

H. Otorhinolaryngology

I. Urology

J. Orthopedics

K. Plastic Surgery/Cosmetic Surgery

L. Dialysis

M. Others

7. Which of the following is the category of medical institution?

A. University hospitals

B. Hospitals with specific functions

C. Tuberculosis hospitals

D. Psychiatric hospitals

E. Hospitals that do not fall into any of the above categories

F. Clinic

G. Others

8. In which prefecture is the facility located?

Select one from 47 prefectures in Japan.

In principle, please answer this questionnaire regarding the current situation at the medical institution where you work. For example, if your department does not have online medical care (hereafter referred to as OMC), but other departments do, please answer that "your medical institution provides OMC ".

9. Does your hospital use an electronic medical record system?

A. Yes

B. No

10. Does your hospital provide OMC?

OMC refers to "the act of conducting medical examinations and diagnoses, communicating the results of diagnoses, and conducting medical treatment in real time between a physician and a patient through information and telecommunications equipment.

A. Implemented and I have also been involved with my own patients in OMC.

**-> Skip to question 11**

B. Although the facility is implementing it, I am not involved with patients in OMC.

**-> Skip to question 27**

C. Not implemented

**-> Skip to question 41**

※Depending on the answers to this question, the following questions will be divided into three patterns.

**Pattern 1: Questionnaire for those who selected “A” for Q10.**

11. What departments in your hospital provide OMC? Please answer as far as you know. If you chose "other," please describe in detail.

A. All the departments

B. Internal Medicine

C. Surgery

D. Pediatrics

E. Obstetrics & Gynecology

F. Psychiatry / Psychosomatic medicine

G. Dermatology

H. Ophthalmology

I. Otorhinolaryngology

J. Urology

K. Orthopedic surgery

L. Plastic surgery/ cosmetic surgery

M. Others:

12. In what kinds of situations does your hospital provide OMC? Please answer as far as you know. If you chose "other," please describe in detail.

A. Explanation of test results

B. Regular medical checkup

C. Doing the same medication as usual

D. Consultation at the scene of a possible infectious disease, such as an outpatient clinic for fever

E. Consultation for sudden onset of illness (acute illness) other than infectious diseases

F. Diagnosis when there is no doctor nearby who specializes in the disease.

G. Diagnosis when it is difficult to go to a hospital (difficulty in transportation, living in a remote area, etc.)

H. Consultation on whether or not to see a doctor

I. Second opinion

J. Nutritional guidance

K. Perinatal counseling services such as parent classes, genetic counseling, infertility counseling, and pregnancy complications counseling.

L. Consultation services related to childcare, such as developmental counseling and support for childcare

M. Conference at the time of introduction of home medical care

N. Not known in detail.

O. Others:

13. Please select any that apply to your hospital's OMC system.

A. Difficult to operate on the medical institution side.

B. Cannot be implemented on the same terminal as the digital medical record system.

C. Although it is possible to use the same terminal as the digital medical record system, it is difficult to use because the software is not integrated. (e.g., the online medical care system cannot be operated within the digital medical record system, or medical records cannot be written within the online medical care system, etc.)

D. Data such as test results cannot be shared on the screen .(it is necessary to use a camera to capture them).

E. Cannot display written documents on the screen. (need to write on a piece of paper and copy it to the camera).

F. There have been problems with the communication environment.

G. Difficulty in operation on the patient's side.

H. No applicable items

14. Compared to face-to-face medical care (hereafter referred to as FMC), do doctors need more assistance of other medical staff (nurses and/or medical clerks) assistance in provision of OMC? In this question, assistance refers to medical assistance, such as preliminary medical interviews, assistance with medical records, and assistance with operations related to OMC.

A. Obviously more in OMC.

B. Somewhat more in OMC.

C. Almost the same for FMC and OMC.

D. Somewhat less in OMC.

E. Obviously less in OMC.

15. Is the medical facility’s operation in providing OMC more complicated than that in providing in FMC? Medical facility’s operation includes making various announcement for patients, accounting, and other clerical procedures.

A. Obviously more complicated in OMC.

B. Somewhat more complicated in OMC.

C. Almost the same for FMC and OMC.

D. Somewhat less complicated in OMC.

E. Obviously less complicated in OMC.

16. Compared to FMC, is the number of patients that can be treated per unit of time higher or lower with OMC?

A. More in OMC.

B. Almost the same for FMC and OMC.

C. Less in OMC.

17. What do you estimate the burden on the patient for OMC compared to FMC?

※Financial burden includes medical fees, transportation costs, phone charges, and other expenses related to the medical examination.

|  | Obviously smaller in OMC | Slightly smaller in OMC | Roughly equivalent to FMC | Slightly larger in OMC | Obviously larger in OMC |
| --- | --- | --- | --- | --- | --- |
| time burden |  |  |  |  |  |
| physical burden |  |  |  |  |  |
| mental burden |  |  |  |  |  |
| financial burden |  |  |  |  |  |

18. Please select the option that best describes the ease of the following seven medical processes when comparing OMC with FMC.

18-1. Intuitive understanding of the severity of the patient's medical condition.

A. Clearly easier in OMC.

B. Somewhat easier in OMC.

C. About the same for FMC and OMC.

D. Somewhat more difficult in OMC.

E. Clearly more difficult in OMC.

18-2. Obtaining patient's physical findings.

A. Clearly easier in OMC.

B. Somewhat easier in OMC.

C. About the same for FMC and OMC.

D. Somewhat more difficult in OMC.

E. Clearly more difficult in OMC.

18-3. Communicate linguistically with patients.

A. Clearly easier in OMC.

B. Somewhat easier in OMC.

C. About the same for FMC and OMC.

D. Somewhat more difficult in OMC.

E. Clearly more difficult in OMC.

18-4. Asking family members about the patient's medical condition and behavior.

A. Clearly easier in OMC.

B. Somewhat easier in OMC.

C. About the same for FMC and OMC.

D. Somewhat more difficult in OMC.

E. Clearly more difficult in OMC.

18-5. Visual understanding of the patient's condition and behavior in his/her home, etc.

A. Clearly easier in OMC.

B. Somewhat easier in OMC.

C. About the same for FMC and OMC.

D. Somewhat more difficult in OMC.

E. Clearly more difficult in OMC.

18-6. Visualization of the environment in which the patient lives, such as in his/her own home.。

A. Clearly easier in OMC.

B. Somewhat easier in OMC.

C. About the same for FMC and OMC.

D. Somewhat more difficult in OMC.

E. Clearly more difficult in OMC.

18-7. Building trust between the patient and the doctor.

A. Clearly easier in OMC.

B. Somewhat easier in OMC.

C. About the same for FMC and OMC.

D. Somewhat more difficult in OMC.

E. Clearly more difficult in OMC.

19. Do you feel that the cost of setting up and maintaining an OMC system is expensive?

A. Obviously expensive.

B. Somewhat expensive.

C. Generally reasonable.

D. Somewhat inexpensive

E. Obviously inexpensive

F. Don't know.

20. Do you know the amount of reimbursement for OMC? (Please give a rough idea of the fees, e.g., lower, higher, or the same as those for FMC.)

A. Yes

B. No

21. The current reimbursement rates for both FMC and OMC are as shown below. Do you think the fees for OMC are high or low?

OMC: Fee for an initial medical examination **251points**

※For institutions that meet the facility criteria and have submitted a notification.

OMC: Fee for a second medical examination **73 points**

FMC: Fee for an initial medical examination **288 points**

FMC: Fee for a second medical examination **73 points**

(For clinics and hospitals with less than 200 general beds)

FMC: Fee for a second medical examination **74 points**

（For hospitals with 200 or more general beds）

In addition, medical management fees (e.g., specified disease treatment management fees, outpatient guidance and management fees for intractable diseases, etc.) are set at approximately 87% of the insurance points FMC in OMC.

A. Medical fees are obviously low.

B. Medical fees are somewhat low.

C. Medical fees are reasonable.

D. Medical fees are somewhat high.

E. Medical fees are obviously high.

F. I don't know.

22. What percentage of your patients would benefit from OMC?

A. 0％

B. 10％

C. 20％

D. 30％

E. 40％

F. 50％

G. 60％

H. 70％

I. 80％

J. 90％

K. 100％

23. If the target patient is approximately how old or younger, do you think it is possible for a person to perform online medical treatment using his/her own nave (PC, smartphone, tablet, etc.)?

A. 50 years old

B. 55 years old

C. 60 years old

D. 65 years old

E. 70 years old

F. 75 years old

G. 80 years old

H. 85 years old

I. Age is an irrelevant factor

24. Please select the option that best describes your opinion on whether FMC or OMC is more appropriate for the following 10 medical procedures.

24-1. Explanation of test results.

A. OMC is clearly more suitable than FMC.

B. OMC is somewhat more suitable than FMC.

C. The same for both FMC and OMC.

D. FMC is clearly more suitable than OMC.

E. FMC is somewhat more suitable than OMC.

24-2. Regular medical checkup.

A. OMC is clearly more suitable than FMC.

B. OMC is somewhat more suitable than FMC.

C. The same for both FMC and OMC.

D. FMC is clearly more suitable than OMC.

E. FMC is somewhat more suitable than OMC.

24-3. Doing the same medication as usual

A. OMC is clearly more suitable than FMC.

B. OMC is somewhat more suitable than FMC.

C. The same for both FMC and OMC.

D. FMC is clearly more suitable than OMC.

E. FMC is somewhat more suitable than OMC.

24-4. Consultation at the scene of a possible infectious disease such as a fever outpatient clinic.

A. OMC is clearly more suitable than FMC.

B. OMC is somewhat more suitable than FMC.

C. The same for both FMC and OMC.

D. FMC is clearly more suitable than OMC.

E. FMC is somewhat more suitable than OMC.

24-5. When there is no doctor nearby who specializes in that disease.

A. OMC is clearly more suitable than FMC.

B. OMC is somewhat more suitable than FMC.

C. The same for both FMC and OMC.

D. FMC is clearly more suitable than OMC.

E. FMC is somewhat more suitable than OMC.

24-6. When it is difficult to go to a hospital (difficulty in transportation, living in a remote area, etc.)

A. OMC is clearly more suitable than FMC.

B. OMC is somewhat more suitable than FMC.

C. The same for both FMC and OMC.

D. FMC is clearly more suitable than OMC.

E. FMC is somewhat more suitable than OMC.

24-7. Second opinion

A. OMC is clearly more suitable than FMC.

B. OMC is somewhat more suitable than FMC.

C. The same for both FMC and OMC.

D. FMC is clearly more suitable than OMC.

E. FMC is somewhat more suitable than OMC.

24-8. Nutritional guidance

A. OMC is clearly more suitable than FMC.

B. OMC is somewhat more suitable than FMC.

C. The same for both FMC and OMC.

D. FMC is clearly more suitable than OMC.

E. FMC is somewhat more suitable than OMC.

24-9. Perinatal counseling services such as parent classes, genetic counseling, infertility counseling, and pregnancy complications counseling.

A. OMC is clearly more suitable than FMC.

B. OMC is somewhat more suitable than FMC.

C. The same for both FMC and OMC.

D. FMC is clearly more suitable than OMC.

E. FMC is somewhat more suitable than OMC.

24-10. Consultation services related to childcare, such as developmental counseling and support for childcare.

A. OMC is clearly more suitable than FMC.

B. OMC is somewhat more suitable than FMC.

C. The same for both FMC and OMC.

D. FMC is clearly more suitable than OMC.

E. FMC is somewhat more suitable than OMC.

25. Please select all of the following factors that you consider to be obstacles to the spread of OMC.

A.OMC increases administrative procedures on the part of the medical institution.

B.OMC requires more time and effort on the part of physician.

C.OMC requires more time and effort on the part of the patient.

D.OMC places a heavy financial burden on medical institution.

E.OMC imposes a heavy financial burden on patients.

F. Difficult for the medical institution to construct a system and communication environment for OMC.

G. Difficult for patients to download applications and build a communication environment for OMC.

H. People concern about the content of the doctor's examination in OMC

I. Talk face-to-face is easier than that online.

J. OMC requires patients to make a hospital visit when tests or procedures are needed.

K. There are not many patients who are suitable for OMC.

L. People concern about the leakage of personal information through OMC.

M. FMC is preferable to OMC for the education of medical students and young doctors.

N. Because OMC itself is not well known.

O. OMC are known, but people do not know when OMC are appropriate or desired.

P. OMC are known, but people do not know which institutions offer OMC.

Q. Because the level of satisfaction with FMC is high and there are not many people who need OMC.

R. Regardless of the level of satisfaction with FMC, people tend to maintain the status quo.

26. Please share any other thoughts you have about OMC, including what you find convenient, what you have trouble with, or what you hope to see in the future.

**Pattern 2: Questionnaire for those who selected “B” for Q10.**

27. What departments in your hospital provide OMC? Please answer as far as you know. If you chose "other," please describe in detail.

A. All the departments

B. Internal Medicine

C. Surgery

D. Pediatrics

E. Obstetrics & Gynecology

F. Psychiatry / Psychosomatic medicine

G. Dermatology

H. Ophthalmology

I. Otorhinolaryngology

J. Urology

K. Orthopedics

L. Plastic and cosmetic surgery

M. Others:

28. In what kinds of situations does your hospital provide OMC? Please answer as far as you know. If you chose "other," please describe in detail. Please select all that apply.

A. Explanation of test results

B. Regular medical checkup

C. Doing the same medication as usual

D. Consultation at the scene of a possible infectious disease, such as an outpatient clinic for fever.

E. Consultation for sudden onset of illness (acute illness) other than infectious diseases

F. Diagnosis when there is no doctor nearby who specializes in the disease.

G. Diagnosis when it is difficult to go to a hospital (difficulty in transportation, living in a remote area, etc.)

H. Consultation on whether or not to see a doctor

I. Second opinion

J. Nutritional guidance

K. Perinatal counseling services such as parent classes, genetic counseling, infertility counseling, and pregnancy complications counseling.

L. Consultation services related to childcare, such as developmental counseling and support for childcare

M. Conference at the time of introduction of home medical care

N. Not known in detail.

O. Others:

29. 【Please answer if you are a doctor.】

If your hospital offers OMC but you are not involved in it, which reason(s) do you give for this? If you chose "other," please provide details if you don’t mind.

※If you are not a physician, please select "Not applicable to this question.

A. Not applicable to this question

B. OMC is available only in other departments in our hospital.

C. OMC is provided only at limited situations such as second opinions.

D. OMC is more time-consuming than FMC.

E. To perform OMC, preparation such as attending a training course is necessary beforehand.

F. My Field of practice is not suitable for OMC.

G. There is no suitable patient for OMC in my own field.

H. I choose to use FMC in order to educate students and young doctors.

I. When presented with the option of OMC, the patient's consent is not obtained.

J. Supposed to be sufficient for FMC and have no motivation to try OMC.

K. Others:

30.Compared to FMC, is the number of patients that can be treated per unit of time higher or lower with OMC?

A. More in OMC.

B. Almost the same for FMC and OMC.

C. Less in OMC.

31. What do you estimate the burden on the patient for OMC compared to FMC?

※Financial burden includes medical fees, transportation costs, phone charges, and other expenses related to the medical examination.

|  | Obviously smaller in OMC | Slightly smaller in OMC | Roughly equivalent to FMC | Slightly larger in OMC | Obviously larger in OMC |
| --- | --- | --- | --- | --- | --- |
| time burden |  |  |  |  |  |
| physical burden |  |  |  |  |  |
| mental burden |  |  |  |  |  |
| financial burden |  |  |  |  |  |

32. Please select the option that best describes the ease of the following seven medical processes when comparing OMC with FMC.

32-1. Intuitive understanding of the severity of the patient's medical condition.

A. Clearly easier in OMC.

B. Somewhat easier in OMC.

C. About the same for FMC and OMC.

D. Somewhat more difficult in OMC.

E. Clearly more difficult in OMC.

32-2. Obtaining patient's physical findings.

A. Clearly easier in OMC.

B. Somewhat easier in OMC.

C. About the same for FMC and OMC.

D. Somewhat more difficult in OMC.

E. Clearly more difficult in OMC.

32-3. Communicate linguistically with patients.

A. Clearly easier in OMC.

B. Somewhat easier in OMC.

C. About the same for FMC and OMC.

D. Somewhat more difficult in OMC.

E. Clearly more difficult in OMC.

32-4. Asking family members about the patient's medical condition and behavior.

A. Clearly easier in OMC.

B. Somewhat easier in OMC.

C. About the same for FMC and OMC.

D. Somewhat more difficult in OMC.

E. Clearly more difficult in OMC.

32-5. Visual understanding of the patient's condition and behavior in his/her home, etc.

A. Clearly easier in OMC.

B. Somewhat easier in OMC.

C. About the same for FMC and OMC.

D. Somewhat more difficult in OMC.

E. Clearly more difficult in OMC.

32-6. Visualization of the environment in which the patient lives, such as in his/her own home.。

A. Clearly easier in OMC.

B. Somewhat easier in OMC.

C. About the same for FMC and OMC.

D. Somewhat more difficult in OMC.

E. Clearly more difficult in OMC.

32-7. Building trust between the patient and the doctor.

A. Clearly easier in OMC.

B. Somewhat easier in OMC.

C. About the same for FMC and OMC.

D. Somewhat more difficult in OMC.

E. Clearly more difficult in OMC.

33. Do you feel that the cost of setting up and maintaining an OMC system is expensive?

A. Obviously expensive.

B. Somewhat expensive.

C. Generally reasonable.

D. Somewhat inexpensive

E. Obviously inexpensive

F. Don't know.

34. Do you know the amount of reimbursement for OMC ? (Please give a rough idea of the fees, e.g., lower, higher, or the same as those for FMC.)

A. Yes

B. No

　35. The current reimbursement rates for both FMC and OMC are as shown below. Do you think the fees for OMC are high or low? [Only one, answer required]

OMC: Fee for an initial medical examination **251points**

※For institutions that meet the facility criteria and have submitted a notification.

OMC: Fee for a second medical examination **73 points**

FMC: Fee for an initial medical examination **288 points**

FMC: Fee for a second medical examination **73 points**

(For clinics and hospitals with less than 200 general beds)

FMC: Fee for a second medical examination **74 points**

（For hospitals with 200 or more general beds）

In addition, medical management fees (e.g., specified disease treatment management fees, outpatient guidance and management fees for intractable diseases, etc.) are set at approximately 87% of the insurance points FMC in OMC.

A. Medical fees are obviously low.

B. Medical fees are somewhat low.

C. Medical fees are reasonable.

D. Medical fees are somewhat high.

E. Medical fees are obviously high.

F. Don't know.

36. What percentage of your patients would benefit from OMC?

A. 0％

B. 10％

C. 20％

D. 30％

E. 40％

F. 50％

G. 60％

H. 70％

I. 80％

J. 90％

K. 100％

　37. If the target patient is approximately how old or younger, do you think it is possible for a person to perform online medical treatment using his/her own terminal (PC, smartphone, tablet, etc.)?

A. 50 years old

B. 55 years old

C. 60 years old

D. 65 years old

E. 70 years old

F. 75 years old

G. 80 years old

H.85 years old

I. Age is an irrelevant factor

38. Please select the option that best describes your opinion on whether FMC or OMC is more appropriate for the following 10 medical procedures.

38-1. Explanation of test results.

A. OMC is clearly more suitable than FMC.

B. OMC is somewhat more suitable than FMC.

C. The same for both FMC and OMC.

D. FMC is clearly more suitable than OMC.

E. FMC is somewhat more suitable than OMC.

38-2. Regular medical checkup.

A. OMC is clearly more suitable than FMC.

B. OMC is somewhat more suitable than FMC.

C. The same for both FMC and OMC.

D. FMC is clearly more suitable than OMC.

E. FMC is somewhat more suitable than OMC.

38-3. Doing the same medication as usual

A. OMC is clearly more suitable than FMC.

B. OMC is somewhat more suitable than FMC.

C. The same for both FMC and OMC.

D. FMC is clearly more suitable than OMC.

E. FMC is somewhat more suitable than OMC.

38-4. Consultation at the scene of a possible infectious disease such as a fever outpatient clinic.

A. OMC is clearly more suitable than FMC.

B. OMC is somewhat more suitable than FMC.

C. The same for both FMC and OMC.

D. FMC is clearly more suitable than OMC.

E. FMC is somewhat more suitable than OMC.

38-5. When there is no doctor nearby who specializes in that disease.

A. OMC is clearly more suitable than FMC.

B. OMC is somewhat more suitable than FMC.

C. The same for both FMC and OMC.

D. FMC is clearly more suitable than OMC.

E. FMC is somewhat more suitable than OMC.

38-6. When it is difficult to go to a hospital (difficulty in transportation, living in a remote area, etc.)

A. OMC is clearly more suitable than FMC.

B. OMC is somewhat more suitable than FMC.

C. The same for both FMC and OMC.

D. FMC is clearly more suitable than OMC.

E. FMC is somewhat more suitable than OMC.

38-7. Second opinion

A. OMC is clearly more suitable than FMC.

B. OMC is somewhat more suitable than FMC.

C. The same for both FMC and OMC.

D. FMC is clearly more suitable than OMC.

E. FMC is somewhat more suitable than OMC.

38-8. Nutritional guidance

A. OMC is clearly more suitable than FMC.

B. OMC is somewhat more suitable than FMC.

C. The same for both FMC and OMC.

D. FMC is clearly more suitable than OMC.

E. FMC is somewhat more suitable than OMC.

38-9. Perinatal counseling services such as parent classes, genetic counseling, infertility counseling, and pregnancy complications counseling.

A. OMC is clearly more suitable than FMC.

B. OMC is somewhat more suitable than FMC.

C. The same for both FMC and OMC.

D. FMC is clearly more suitable than OMC.

E. FMC is somewhat more suitable than OMC.

38-10. Consultation services related to childcare, such as developmental counseling and support for childcare.

A. OMC is clearly more suitable than FMC.

B. OMC is somewhat more suitable than FMC.

C. The same for both FMC and OMC.

D. FMC is clearly more suitable than OMC.

E. FMC is somewhat more suitable than OMC.

39. Please select all of the following factors that you consider to be obstacles to the spread of OMC.

A. OMC increases administrative procedures on the part of medical institutions.

B. OMC requires more time and effort on the part of physicians

C. OMC requires more time and effort on the part of the patient.

D. OMC places a heavy financial burden on the medical institution

E. OMC imposes a heavy financial burden on patients

F. Difficult for the medical institution to construct a system and communication environment for OMC.

G. Difficult for patients to download applications and build a communication environment for OMC.

H. People concern about the content of the doctor's examination in OMC

I. Talk face-to-face is easier than that online

J. OMC requires patients to make a hospital visit when tests or procedures are needed

K. There are not many patients who are suitable for OMC.

L. People concern about the leakage of personal information through OMC

M. FMC is preferable to OMC for the education of medical students and young doctors

N. Because OMC itself is not well known

O. OMC are known, but people do not know when OMC are appropriate or desired.

P. OMC are known, but people do not know which institutions offer OMC.

Q. Because the level of satisfaction with FMC is high and there are not many people who need OMC.

R. Regardless of the level of satisfaction with FMC, people tend to maintain the status quo.

40. Please share any other thoughts you have about OMC, including what you find convenient, what you have trouble with, or what you hope to see in the future.

**Pattern 3: Questionnaire for those who selected “C” for Q10.**

41. What do you estimate the burden on the patient for OMC compared to FMC?

※Financial burden includes medical fees, transportation costs, phone charges, and other expenses related to the medical examination.

|  | Obviously smaller in OMC | Slightly smaller in OMC | Roughly equivalent to FMC | Slightly larger in OMC | Obviously larger in OMC |
| --- | --- | --- | --- | --- | --- |
| time burden |  |  |  |  |  |
| physical burden |  |  |  |  |  |
| mental burden |  |  |  |  |  |
| financial burden |  |  |  |  |  |

42. Please select the option that best describes the ease of the following seven medical processes when comparing OMC with FMC.

42-1. Intuitive understanding of the severity of the patient's medical condition.

A. Clearly easier in OMC.

B. Somewhat easier in OMC.

C. About the same for FMC and OMC.

D. Somewhat more difficult in OMC.

E. Clearly more difficult in OMC.

42-2. Obtaining patient's physical findings.

A. Clearly easier in OMC.

B. Somewhat easier in OMC.

C. About the same for FMC and OMC.

D. Somewhat more difficult in OMC.

E. Clearly more difficult in OMC.

42-3. Communicate linguistically with patients.

A. Clearly easier in OMC.

B. Somewhat easier in OMC.

C. About the same for FMC and OMC.

D. Somewhat more difficult in OMC.

E. Clearly more difficult in OMC.

42-4. Asking family members about the patient's medical condition and behavior.

A. Clearly easier in OMC.

B. Somewhat easier in OMC.

C. About the same for FMC and OMC.

D. Somewhat more difficult in OMC.

E. Clearly more difficult in OMC.

42-5. Visual understanding of the patient's condition and behavior in his/her home, etc.

A. Clearly easier in OMC.

B. Somewhat easier in OMC.

C. About the same for FMC and OMC.

D. Somewhat more difficult in OMC.

E. Clearly more difficult in OMC.

42-6. Visualization of the environment in which the patient lives, such as in his/her own home.。

A. Clearly easier in OMC.

B. Somewhat easier in OMC.

C. About the same for FMC and OMC.

D. Somewhat more difficult in OMC.

E. Clearly more difficult in OMC.

42-7. Building trust between the patient and the doctor.

A. Clearly easier in OMC.

B. Somewhat easier in OMC.

C. About the same for FMC and OMC.

D. Somewhat more difficult in OMC.

E. Clearly more difficult in OMC.

43. Do you feel that the cost of setting up and maintaining an OMC system is expensive?

A. Obviously expensive.

B. Somewhat expensive.

C. Generally reasonable.

D. Somewhat inexpensive

E. Obviously inexpensive

F. Don't know.

44. Do you know the amount of reimbursement for OMC? (Please give a rough idea of the fees, e.g., lower, higher, or the same as those for FMC.

A. Yes

B. No

45. The current reimbursement rates for both FMC and OMC are as shown below. Do you think the fees for OMC are high or low?

OMC: Fee for an initial medical examination **251points**

※For institutions that meet the facility criteria and have submitted a notification.

OMC: Fee for a second medical examination **73 points**

FMC: Fee for an initial medical examination **288 points**

FMC: Fee for a second medical examination **73 points**

(For clinics and hospitals with less than 200 general beds)

FMC: Fee for a second medical examination **74 points**

（For hospitals with 200 or more general beds）

In addition, medical management fees (e.g., specified disease treatment management fees, outpatient guidance and management fees for intractable diseases, etc.) are set at approximately 87% of the insurance points FMC in OMC.

A. Medical fees are obviously low.

B. Medical fees are somewhat low.

C. Medical fees are reasonable.

D. Medical fees are somewhat high.

E. Medical fees are obviously high.

F. I don't know.

46. What percentage of your patients would benefit from OMC?

A. 0％

B. 10％

C. 20％

D. 30％

E. 40％

F. 50％

G. 60％

H. 70％

I. 80％

J. 90％

K. 100％

47. If the target patient is approximately how old or younger, do you think it is possible for a person to perform online medical treatment using his/her own nave (PC, smartphone, tablet, etc.)?

A. 50 years old

B. 55 years old

C. 60 years old

D. 65 years old

E. 70 years old

F. 75 years old

G. 80 years old

H. 85 years old

I. Age is an irrelevant factor

48. Please select the option that best describes your opinion on whether FMC or OMC is more appropriate for the following 10 medical procedures.

48-1. Explanation of test results.

A. OMC is clearly more suitable than FMC.

B. OMC is somewhat more suitable than FMC.

C. The same for both FMC and OMC.

D. FMC is clearly more suitable than OMC.

E. FMC is somewhat more suitable than OMC.

48-2. Regular medical checkup.

A. OMC is clearly more suitable than FMC.

B. OMC is somewhat more suitable than FMC.

C. The same for both FMC and OMC.

D. FMC is clearly more suitable than OMC.

E. FMC is somewhat more suitable than OMC.

48-3. Doing the same medication as usual

A. OMC is clearly more suitable than FMC.

B. OMC is somewhat more suitable than FMC.

C. The same for both FMC and OMC.

D. FMC is clearly more suitable than OMC.

E. FMC is somewhat more suitable than OMC.

48-4. Consultation at the scene of a possible infectious disease such as a fever outpatient clinic.

A. OMC is clearly more suitable than FMC.

B. OMC is somewhat more suitable than FMC.

C. The same for both FMC and OMC.

D. FMC is clearly more suitable than OMC.

E. FMC is somewhat more suitable than OMC.

48-5. When there is no doctor nearby who specializes in that disease.

A. OMC is clearly more suitable than FMC.

B. OMC is somewhat more suitable than FMC.

C. The same for both FMC and OMC.

D. FMC is clearly more suitable than OMC.

E. FMC is somewhat more suitable than OMC.

48-6. When it is difficult to go to a hospital (difficulty in transportation, living in a remote area, etc.)

A. OMC is clearly more suitable than FMC.

B. OMC is somewhat more suitable than FMC.

C. The same for both FMC and OMC.

D. FMC is clearly more suitable than OMC.

E. FMC is somewhat more suitable than OMC.

48-7. Second opinion

A. OMC is clearly more suitable than FMC.

B. OMC is somewhat more suitable than FMC.

C. The same for both FMC and OMC.

D. FMC is clearly more suitable than OMC.

E. FMC is somewhat more suitable than OMC.

48-8. Nutritional guidance

A. OMC is clearly more suitable than FMC.

B. OMC is somewhat more suitable than FMC.

C. The same for both FMC and OMC.

D. FMC is clearly more suitable than OMC.

E. FMC is somewhat more suitable than OMC.

48-9. Perinatal counseling services such as parent classes, genetic counseling, infertility counseling, and pregnancy complications counseling.

A. OMC is clearly more suitable than FMC.

B. OMC is somewhat more suitable than FMC.

C. The same for both FMC and OMC.

D. FMC is clearly more suitable than OMC.

E. FMC is somewhat more suitable than OMC.

48-10. Consultation services related to childcare, such as developmental counseling and support for childcare.

A. OMC is clearly more suitable than FMC.

B. OMC is somewhat more suitable than FMC.

C. The same for both FMC and OMC.

D. FMC is clearly more suitable than OMC.

E. FMC is somewhat more suitable than OMC.

49. Please select all of the following factors that you consider to be obstacles to the spread of OMC.

A. OMC increases administrative procedures on the part of medical institutions.

B. OMC requires more time and effort on the part of physicians

C. OMC requires more time and effort on the part of the patient.

D. OMC places a heavy financial burden on the medical institution

E. OMC imposes a heavy financial burden on patients

F. Difficult for the medical institution to construct a system and communication environment for OMC.

G. Difficult for patients to download applications and build a communication environment for OMC.

H. People concern about the content of the doctor's examination in OMC

I. Talk face-to-face is easier than that online

J. OMC requires patients to make a hospital visit when tests or procedures are needed

K. There are not many patients who are suitable for OMC.

L. People concern about the leakage of personal information through OMC

M. FMC is preferable to OMC for the education of medical students and young doctors

N. Because OMC itself is not well known

O. OMC are known, but people do not know when OMC are appropriate or desired.

P. OMC are known, but people do not know which institutions offer OMC.

Q. Because the level of satisfaction with FMC is high and there are not many people who need OMC.

R. Regardless of the level of satisfaction with FMC, people tend to maintain the status quo.

50. Please share any other thoughts you have about OMC, including what you find convenient, what you have trouble with, or what you hope to see in the future.
