# Supplementary material for: Issues in the Adoption of Online Medical Care: Cross-Sectional Questionnaire Survey
Source: J Med Internet Res. 2024 Nov 1;26:e64159. doi: 10.2196/64159 (PMC11568393; doi:10.2196/64159)
Supplement: Multimedia Appendix 6 [file jmir_v26i1e64159_app6.pptx]

## Slide 1
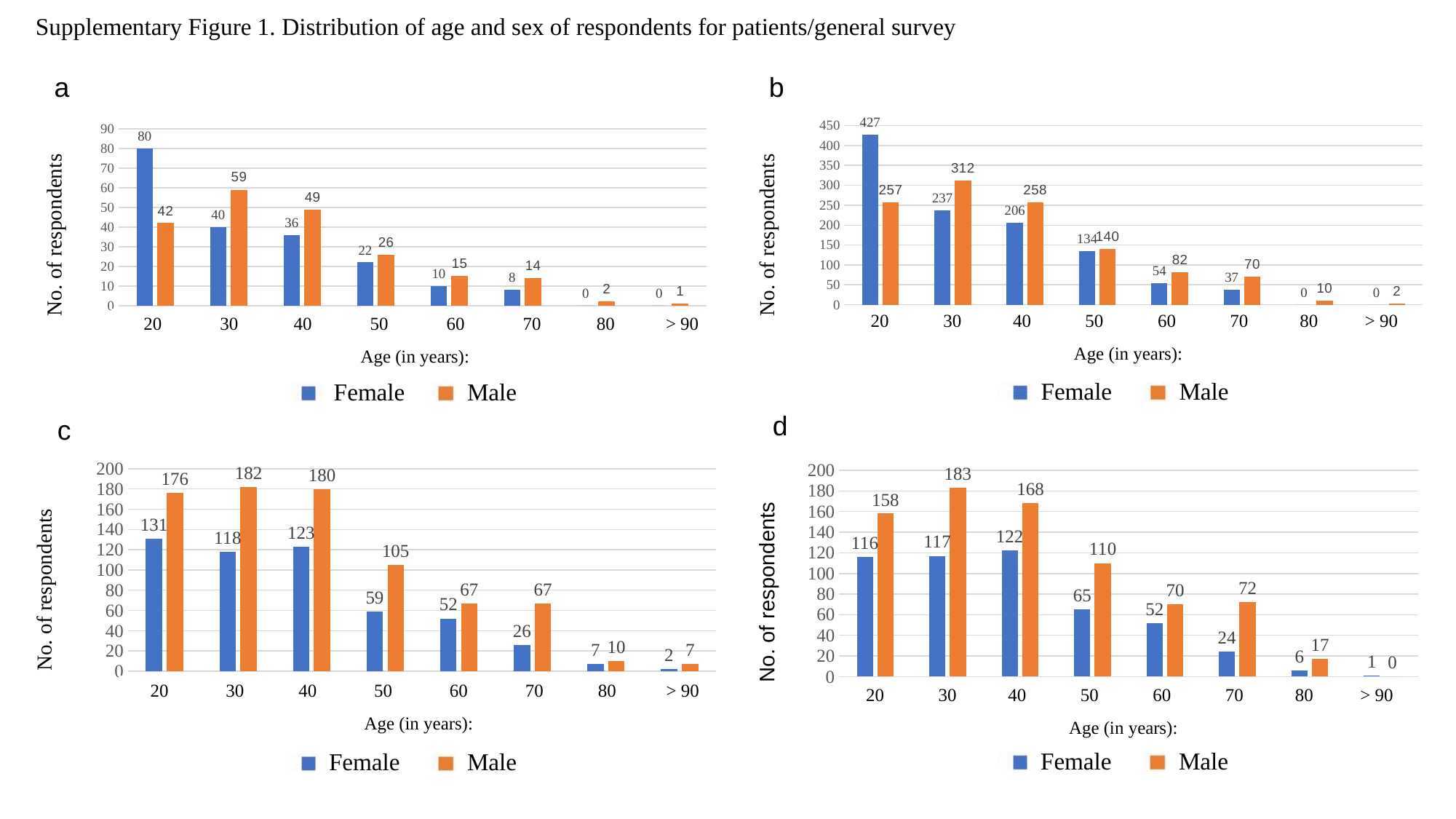

Supplementary Figure 1. Distribution of age and sex of respondents for patients/general survey
### Chart
| Category | 女性 | 男性 |
|---|---|---|
| 20歳代 | 80.0 | 42.0 |
| 30歳代 | 40.0 | 59.0 |
| 40歳代 | 36.0 | 49.0 |
| 50歳代 | 22.0 | 26.0 |
| 60歳代 | 10.0 | 15.0 |
| 70歳代 | 8.0 | 14.0 |
| 80歳代 | 0.0 | 2.0 |
| 90歳代以上 | 0.0 | 1.0 |
### Chart
| Category | 女性 | 男性 |
|---|---|---|
| 20歳代 | 427.0 | 257.0 |
| 30歳代 | 237.0 | 312.0 |
| 40歳代 | 206.0 | 258.0 |
| 50歳代 | 134.0 | 140.0 |
| 60歳代 | 54.0 | 82.0 |
| 70歳代 | 37.0 | 70.0 |
| 80歳代 | 0.0 | 10.0 |
| 90歳代以上 | 0.0 | 2.0 |b
a
No. of respondents
No. of respondents
| 20 | 30 | 40 | 50 | 60 | 70 | 80 | > 90 |
| --- | --- | --- | --- | --- | --- | --- | --- |
| Age (in years): | | | | | | | |
| 20 | 30 | 40 | 50 | 60 | 70 | 80 | > 90 |
| --- | --- | --- | --- | --- | --- | --- | --- |
| Age (in years): | | | | | | | |
Female
Male
Female
Male
### Chart
| Category | 女性 | 男性 |
|---|---|---|
| 20歳代 | 116.0 | 158.0 |
| 30歳代 | 117.0 | 183.0 |
| 40歳代 | 122.0 | 168.0 |
| 50歳代 | 65.0 | 110.0 |
| 60歳代 | 52.0 | 70.0 |
| 70歳代 | 24.0 | 72.0 |
| 80歳代 | 6.0 | 17.0 |
| 90歳代以上 | 1.0 | 0.0 |
### Chart
| Category | 女性 | 男性 |
|---|---|---|
| 20歳代 | 131.0 | 176.0 |
| 30歳代 | 118.0 | 182.0 |
| 40歳代 | 123.0 | 180.0 |
| 50歳代 | 59.0 | 105.0 |
| 60歳代 | 52.0 | 67.0 |
| 70歳代 | 26.0 | 67.0 |
| 80歳代 | 7.0 | 10.0 |
| 90歳代以上 | 2.0 | 7.0 |d
c
No. of respondents
No. of respondents
| 20 | 30 | 40 | 50 | 60 | 70 | 80 | > 90 |
| --- | --- | --- | --- | --- | --- | --- | --- |
| Age (in years): | | | | | | | |
| 20 | 30 | 40 | 50 | 60 | 70 | 80 | > 90 |
| --- | --- | --- | --- | --- | --- | --- | --- |
| Age (in years): | | | | | | | |
Female
Male
Female
Male
1

## Slide 2
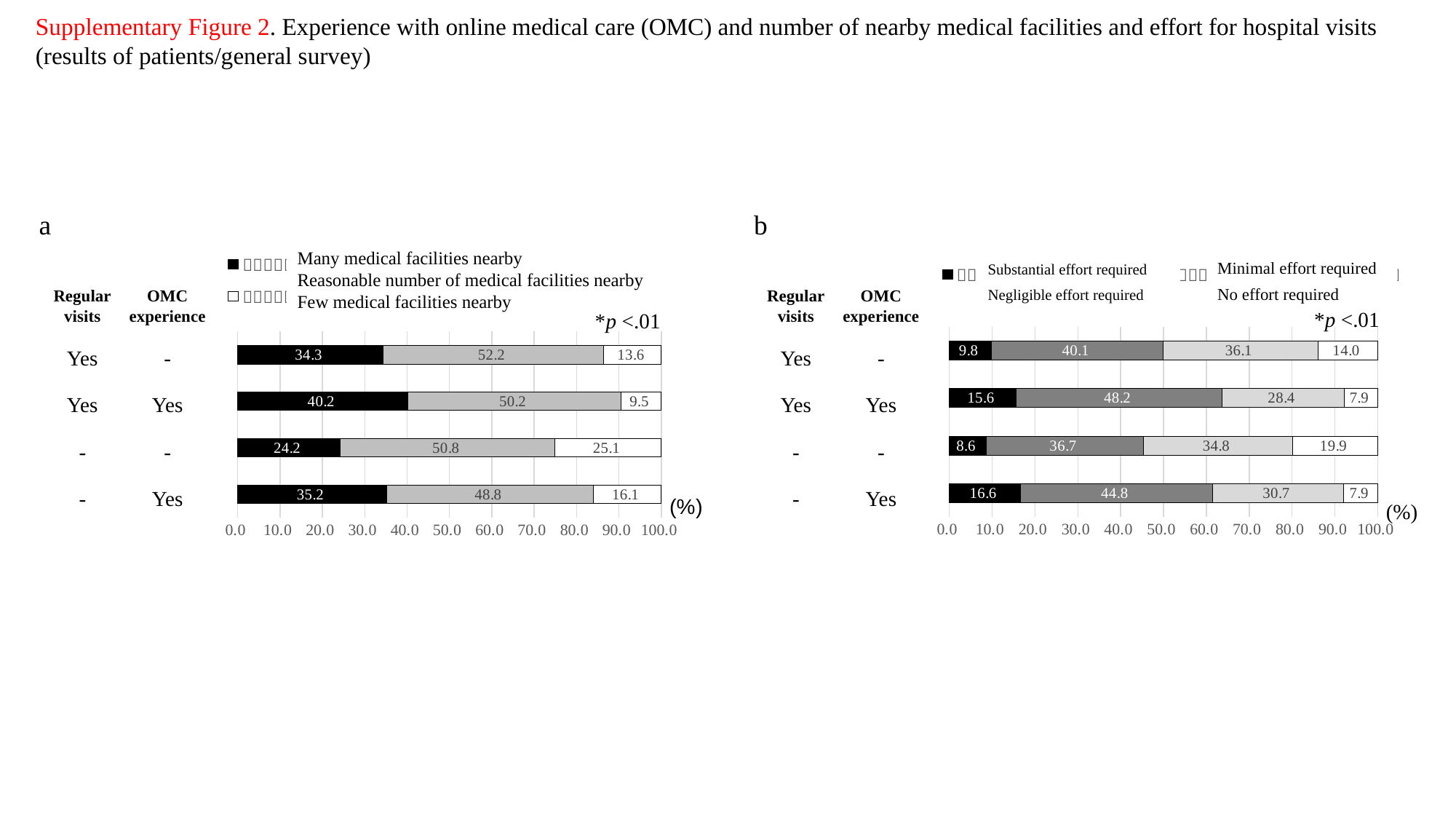

Supplementary Figure 2. Experience with online medical care (OMC) and number of nearby medical facilities and effort for hospital visits (results of patients/general survey)
### Chart
| Category | 近くに医療機関が数多く存在する | 近くに医療機関がそれなりの数存在する | 近くに医療機関はあまり存在しない |
|---|---|---|---|
| 定期通院先なし/オンライン診療経験あり | 35.15 | 48.76 | 16.09 |
| 定期通院先なし/オンライン診療経験なし | 24.17 | 50.76 | 25.07 |
| 定期通院先あり/オンライン診療経験あり | 40.24 | 50.23 | 9.53 |
| 定期通院先あり/オンライン診療経験なし | 34.27 | 52.15 | 13.58 |a
b
### Chart
| Category | かなり手間がかかる | やや手間がかかる | あまり手間はかからない | 全く手間はかからない |
|---|---|---|---|---|
| 定期通院先なし/オンライン診療経験あり | 16.58 | 44.8 | 30.69 | 7.92 |
| 定期通院先なし/オンライン診療経験なし | 8.63 | 36.7 | 34.77 | 19.9 |
| 定期通院先あり/オンライン診療経験あり | 15.55 | 48.17 | 28.43 | 7.85 |
| 定期通院先あり/オンライン診療経験なし | 9.84 | 40.05 | 36.14 | 13.97 |Many medical facilities nearby
Reasonable number of medical facilities nearby
Few medical facilities nearby
Minimal effort required
No effort required
Substantial effort required
Negligible effort required
Regular
visits
OMC
experience
Regular
visits
OMC
experience
*p <.01
*p <.01
Yes
-
Yes
-
Yes
Yes
Yes
Yes
-
-
-
-
-
Yes
-
Yes
(%)

## Slide 3
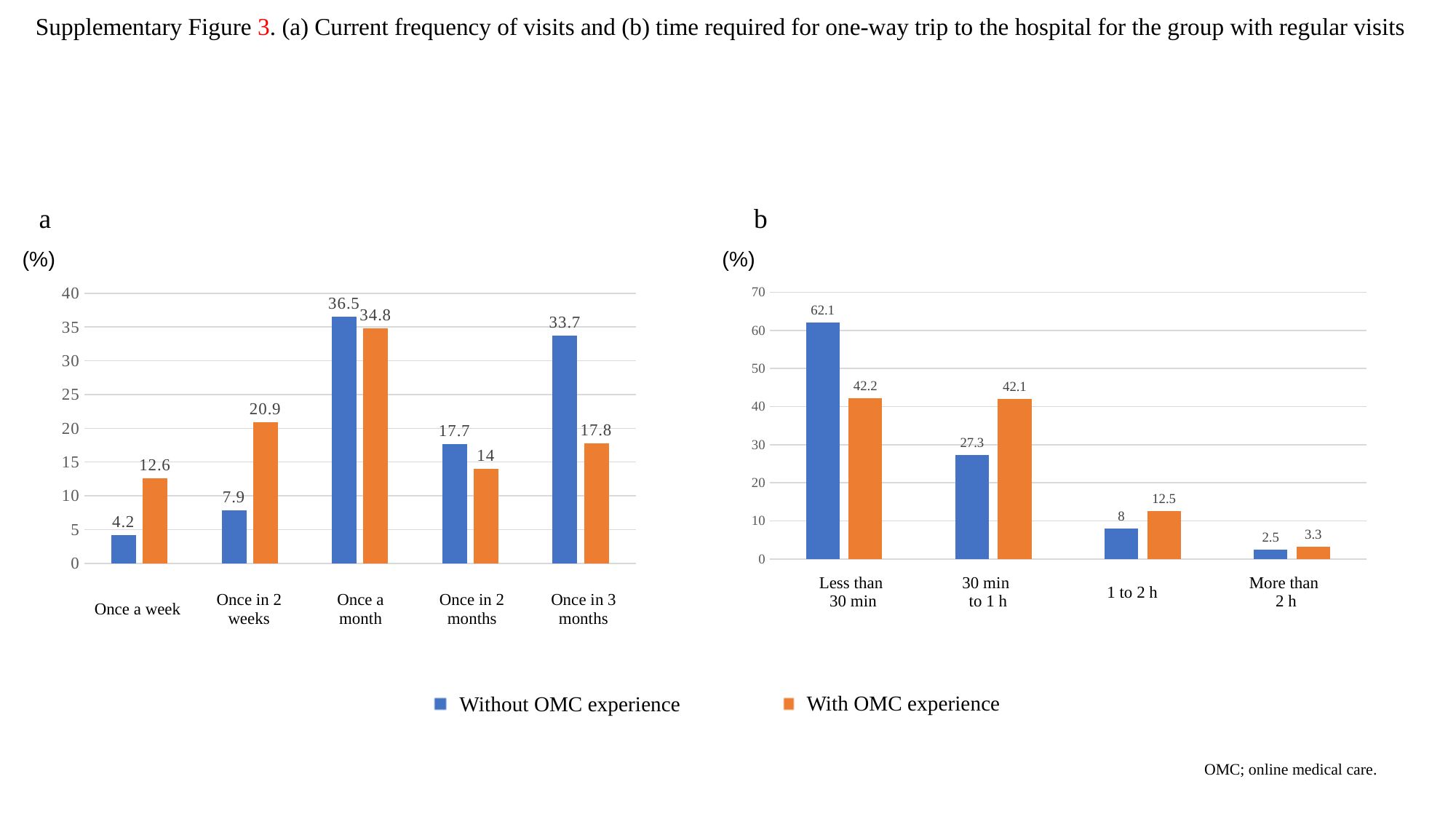

Supplementary Figure 3. (a) Current frequency of visits and (b) time required for one-way trip to the hospital for the group with regular visits
a
b
### Chart
| Category | オンライン診療経験なし群 | オンライン診療経験あり群 |
|---|---|---|
| 週1回程度 | 4.2 | 12.6 |
| 2週に1回程度 | 7.9 | 20.9 |
| 月1回程度 | 36.5 | 34.8 |
| 2ヶ月に1回程度 | 17.7 | 14.0 |
| 3ヶ月に1回程度 | 33.7 | 17.8 |
### Chart
| Category | オンライン診療経験なし群 | オンライン診療経験あり群 |
|---|---|---|
| 30分未満 | 62.1 | 42.2 |
| 30分以上1時間未満 | 27.3 | 42.1 |
| 1時間以上2時間未満 | 8.0 | 12.5 |
| 2時間以上 | 2.5 | 3.3 |(%)
(%)
| Once a week | Once in 2 weeks | Once a month | Once in 2 months | Once in 3 months |
| --- | --- | --- | --- | --- |
| | | | | |
| Less than 30 min | 30 min to 1 h | 1 to 2 h | More than 2 h |
| --- | --- | --- | --- |
| | | | |
With OMC experience
Without OMC experience
OMC; online medical care.

## Slide 4
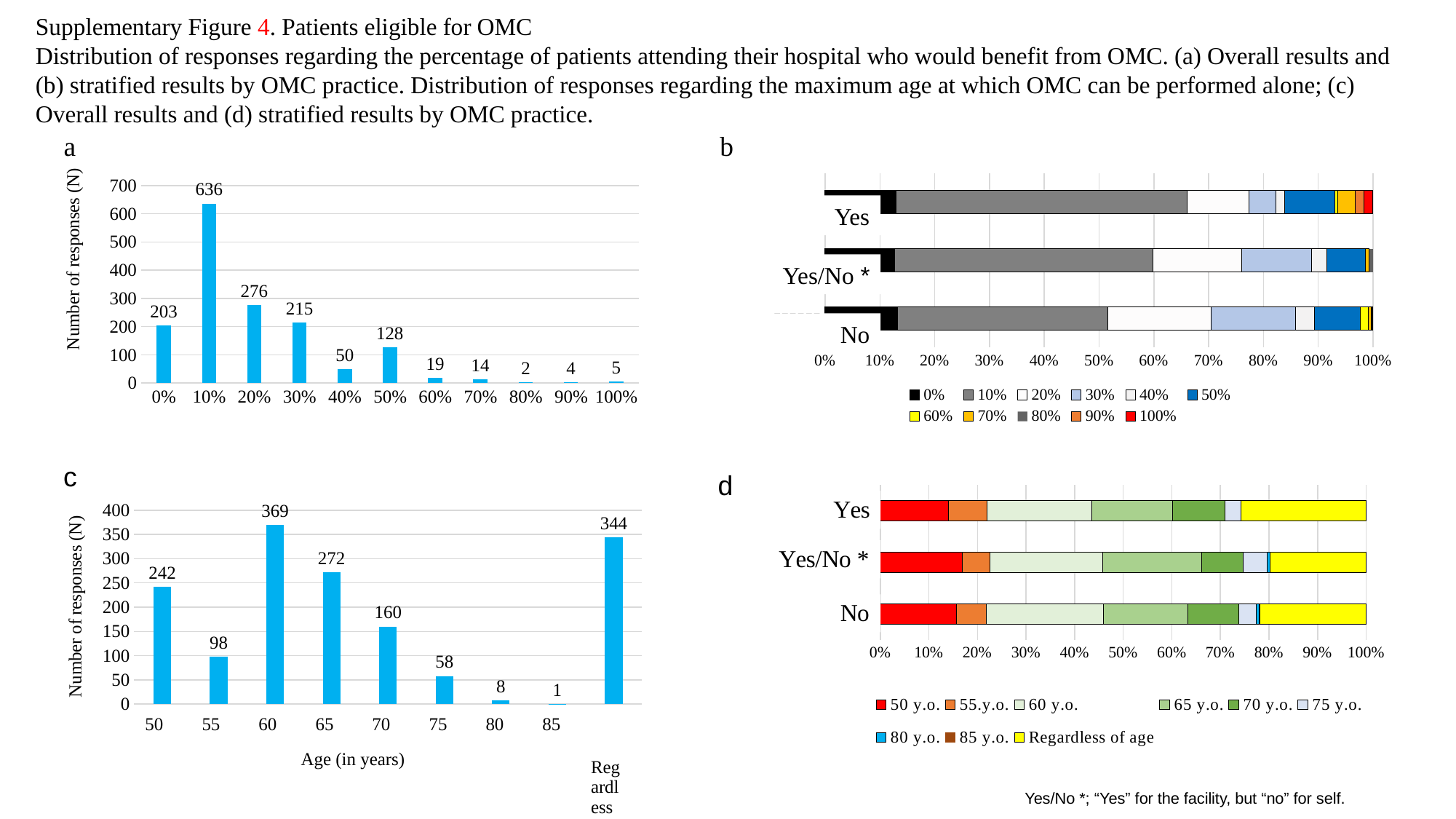

Supplementary Figure 4. Patients eligible for OMC
Distribution of responses regarding the percentage of patients attending their hospital who would benefit from OMC. (a) Overall results and (b) stratified results by OMC practice. Distribution of responses regarding the maximum age at which OMC can be performed alone; (c) Overall results and (d) stratified results by OMC practice.
a
b
### Chart
| Category | |
|---|---|
| 0% | 203.0 |
| 10% | 636.0 |
| 20% | 276.0 |
| 30% | 215.0 |
| 40% | 50.0 |
| 50% | 128.0 |
| 60% | 19.0 |
| 70% | 14.0 |
| 80% | 2.0 |
| 90% | 4.0 |
| 100% | 5.0 |
### Chart
| Category | 0% | 10% | 20% | 30% | 40% | 50% | 60% | 70% | 80% | 90% | 100% |
|---|---|---|---|---|---|---|---|---|---|---|---|
| 行っていない | 161.0 | 470.0 | 232.0 | 188.0 | 43.0 | 101.0 | 18.0 | 7.0 | 1.0 | 1.0 | 2.0 |
| 自身は行っていない | 18.0 | 67.0 | 23.0 | 18.0 | 4.0 | 10.0 | 0.0 | 1.0 | 1.0 | 0.0 | 0.0 |
| 自身も行っている | 24.0 | 99.0 | 21.0 | 9.0 | 3.0 | 17.0 | 1.0 | 6.0 | 0.0 | 3.0 | 3.0 |Yes
Yes/No *
No
c
d
### Chart
| Category | 50 y.o. | 55.y.o. | 60 y.o. | 65 y.o. | 70 y.o. | 75 y.o. | 80 y.o. | 85 y.o. | Regardless of age |
|---|---|---|---|---|---|---|---|---|---|
| No | 192.0 | 75.0 | 296.0 | 212.0 | 128.0 | 45.0 | 7.0 | 1.0 | 268.0 |
| Yes/No * | 24.0 | 8.0 | 33.0 | 29.0 | 12.0 | 7.0 | 1.0 | 0.0 | 28.0 |
| Yes | 26.0 | 15.0 | 40.0 | 31.0 | 20.0 | 6.0 | 0.0 | 0.0 | 48.0 |
### Chart
| Category | |
|---|---|
| 50歳 | 242.0 |
| 55歳 | 98.0 |
| 60歳 | 369.0 |
| 65歳 | 272.0 |
| 70歳 | 160.0 |
| 75歳 | 58.0 |
| 80歳 | 8.0 |
| 85歳 | 1.0 |
| 年齢は関係ないと考える | 344.0 || 50 | 55 | 60 | 65 | 70 | 75 | 80 | 85 | Regardless of age |
| --- | --- | --- | --- | --- | --- | --- | --- | --- |
| Age (in years) | | | | | | | | |
Yes/No *; “Yes” for the facility, but “no” for self.

## Slide 5
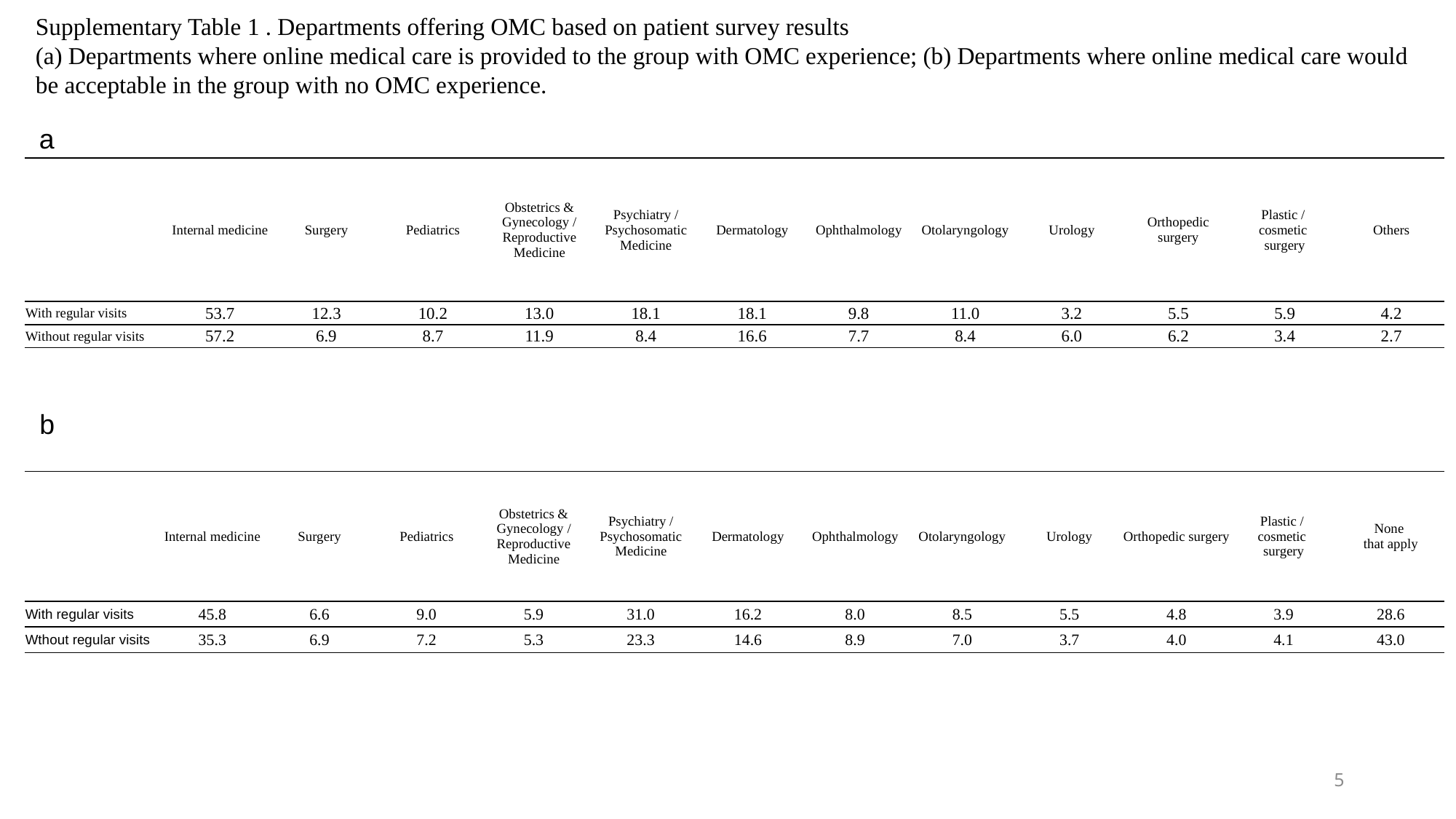

Supplementary Table 1 . Departments offering OMC based on patient survey results
(a) Departments where online medical care is provided to the group with OMC experience; (b) Departments where online medical care would be acceptable in the group with no OMC experience.
a
| | Internal medicine | Surgery | Pediatrics | Obstetrics & Gynecology / Reproductive Medicine | Psychiatry / Psychosomatic Medicine | Dermatology | Ophthalmology | Otolaryngology | Urology | Orthopedic surgery | Plastic / cosmetic surgery | Others |
| --- | --- | --- | --- | --- | --- | --- | --- | --- | --- | --- | --- | --- |
| With regular visits | 53.7 | 12.3 | 10.2 | 13.0 | 18.1 | 18.1 | 9.8 | 11.0 | 3.2 | 5.5 | 5.9 | 4.2 |
| Without regular visits | 57.2 | 6.9 | 8.7 | 11.9 | 8.4 | 16.6 | 7.7 | 8.4 | 6.0 | 6.2 | 3.4 | 2.7 |
b
| | Internal medicine | Surgery | Pediatrics | Obstetrics & Gynecology / Reproductive Medicine | Psychiatry / Psychosomatic Medicine | Dermatology | Ophthalmology | Otolaryngology | Urology | Orthopedic surgery | Plastic / cosmetic surgery | None that apply |
| --- | --- | --- | --- | --- | --- | --- | --- | --- | --- | --- | --- | --- |
| With regular visits | 45.8 | 6.6 | 9.0 | 5.9 | 31.0 | 16.2 | 8.0 | 8.5 | 5.5 | 4.8 | 3.9 | 28.6 |
| Wthout regular visits | 35.3 | 6.9 | 7.2 | 5.3 | 23.3 | 14.6 | 8.9 | 7.0 | 3.7 | 4.0 | 4.1 | 43.0 |
5

## Slide 6
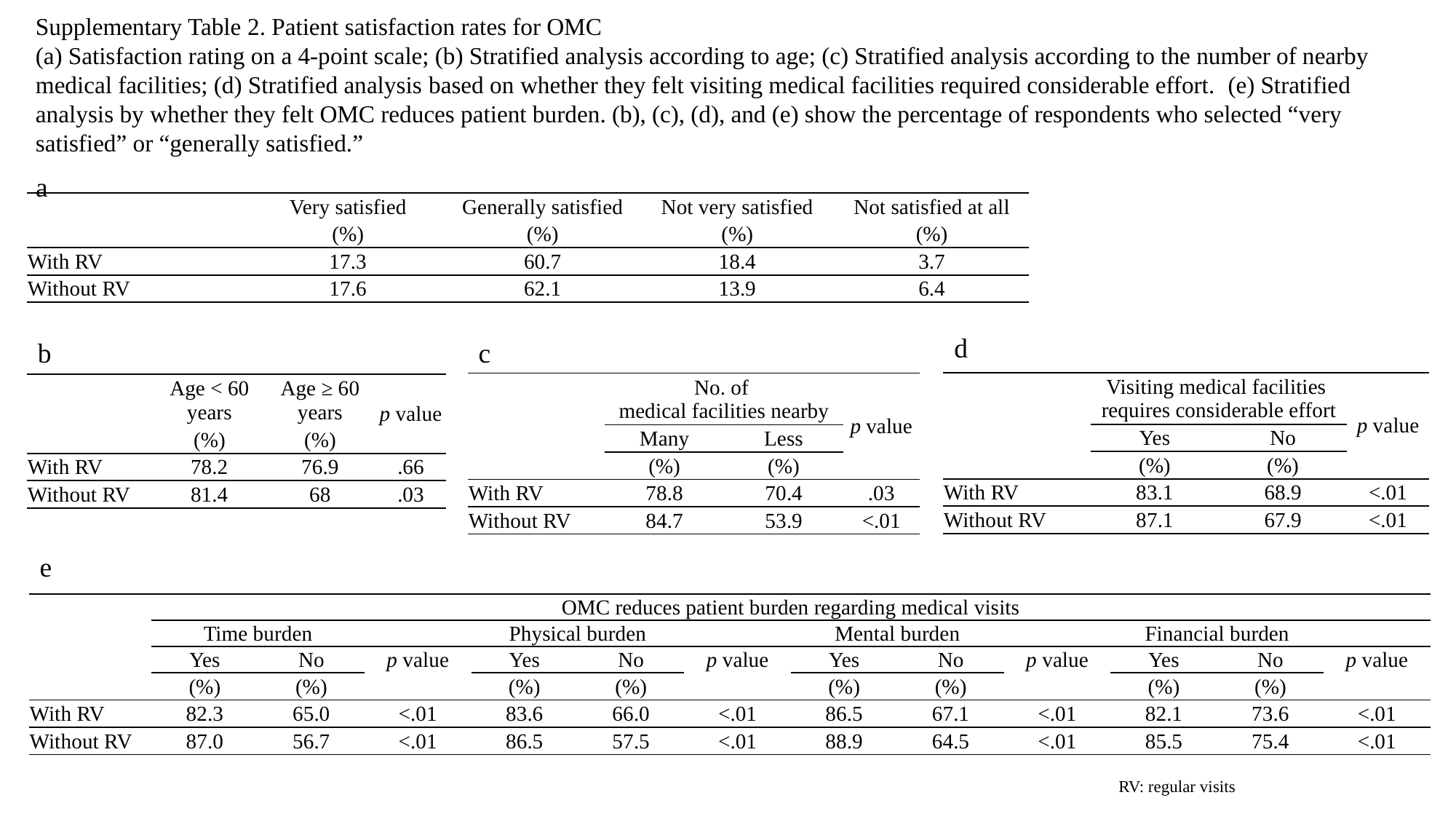

Supplementary Table 2. Patient satisfaction rates for OMC
(a) Satisfaction rating on a 4-point scale; (b) Stratified analysis according to age; (c) Stratified analysis according to the number of nearby medical facilities; (d) Stratified analysis based on whether they felt visiting medical facilities required considerable effort. (e) Stratified analysis by whether they felt OMC reduces patient burden. (b), (c), (d), and (e) show the percentage of respondents who selected “very satisfied” or “generally satisfied.”
a
| | Very satisfied | Generally satisfied | Not very satisfied | Not satisfied at all |
| --- | --- | --- | --- | --- |
| | (%) | (%) | (%) | (%) |
| With RV | 17.3 | 60.7 | 18.4 | 3.7 |
| Without RV | 17.6 | 62.1 | 13.9 | 6.4 |
d
b
c
| | Visiting medical facilities requires considerable effort | | p value |
| --- | --- | --- | --- |
| | Yes | No | |
| | (%) | (%) | |
| With RV | 83.1 | 68.9 | <.01 |
| Without RV | 87.1 | 67.9 | <.01 |
| | No. of medical facilities nearby | | p value |
| --- | --- | --- | --- |
| | Many | Less | |
| | (%) | (%) | |
| With RV | 78.8 | 70.4 | .03 |
| Without RV | 84.7 | 53.9 | <.01 |
| | Age ˂ 60 years | Age ≥ 60 years | p value |
| --- | --- | --- | --- |
| | (%) | (%) | |
| With RV | 78.2 | 76.9 | .66 |
| Without RV | 81.4 | 68 | .03 |
e
| | OMC reduces patient burden regarding medical visits | | | | | | | | | | | |
| --- | --- | --- | --- | --- | --- | --- | --- | --- | --- | --- | --- | --- |
| | Time burden | | | Physical burden | | | Mental burden | | | Financial burden | | |
| | Yes | No | p value | Yes | No | p value | Yes | No | p value | Yes | No | p value |
| | (%) | (%) | | (%) | (%) | | (%) | (%) | | (%) | (%) | |
| With RV | 82.3 | 65.0 | <.01 | 83.6 | 66.0 | <.01 | 86.5 | 67.1 | <.01 | 82.1 | 73.6 | <.01 |
| Without RV | 87.0 | 56.7 | <.01 | 86.5 | 57.5 | <.01 | 88.9 | 64.5 | <.01 | 85.5 | 75.4 | <.01 |
RV: regular visits

## Slide 7
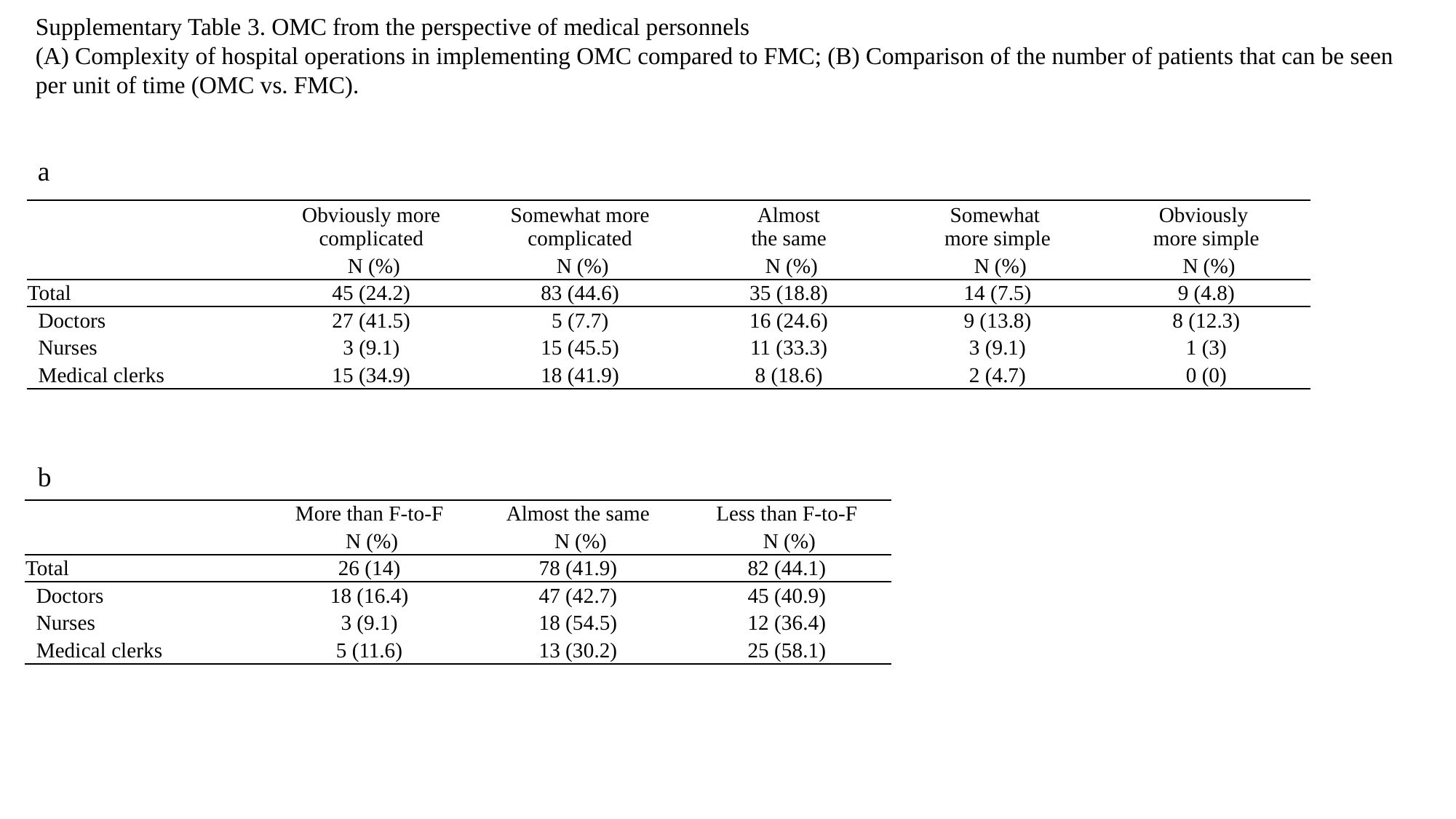

Supplementary Table 3. OMC from the perspective of medical personnels
(A) Complexity of hospital operations in implementing OMC compared to FMC; (B) Comparison of the number of patients that can be seen per unit of time (OMC vs. FMC).
a
| | Obviously more complicated | Somewhat more complicated | Almost the same | Somewhat more simple | Obviously more simple |
| --- | --- | --- | --- | --- | --- |
| | N (%) | N (%) | N (%) | N (%) | N (%) |
| Total | 45 (24.2) | 83 (44.6) | 35 (18.8) | 14 (7.5) | 9 (4.8) |
| Doctors | 27 (41.5) | 5 (7.7) | 16 (24.6) | 9 (13.8) | 8 (12.3) |
| Nurses | 3 (9.1) | 15 (45.5) | 11 (33.3) | 3 (9.1) | 1 (3) |
| Medical clerks | 15 (34.9) | 18 (41.9) | 8 (18.6) | 2 (4.7) | 0 (0) |
b
| | More than F-to-F | Almost the same | Less than F-to-F |
| --- | --- | --- | --- |
| | N (%) | N (%) | N (%) |
| Total | 26 (14) | 78 (41.9) | 82 (44.1) |
| Doctors | 18 (16.4) | 47 (42.7) | 45 (40.9) |
| Nurses | 3 (9.1) | 18 (54.5) | 12 (36.4) |
| Medical clerks | 5 (11.6) | 13 (30.2) | 25 (58.1) |

## Slide 8
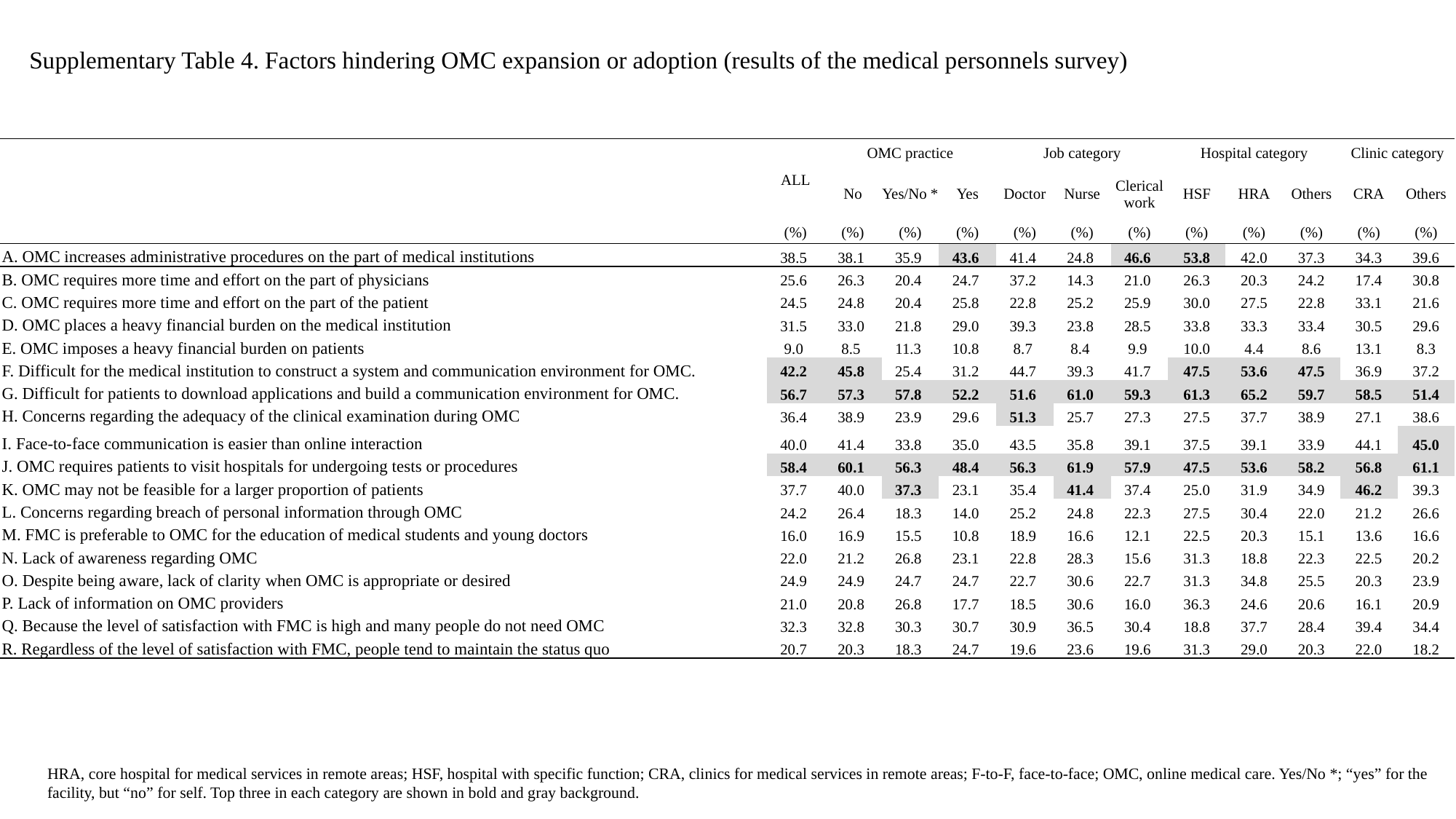

Supplementary Table 4. Factors hindering OMC expansion or adoption (results of the medical personnels survey)
| | ALL | OMC practice | | | Job category | | | Hospital category | | | Clinic category | |
| --- | --- | --- | --- | --- | --- | --- | --- | --- | --- | --- | --- | --- |
| | | No | Yes/No \* | Yes | Doctor | Nurse | Clerical work | HSF | HRA | Others | CRA | Others |
| | (%) | (%) | (%) | (%) | (%) | (%) | (%) | (%) | (%) | (%) | (%) | (%) |
| A. OMC increases administrative procedures on the part of medical institutions | 38.5 | 38.1 | 35.9 | 43.6 | 41.4 | 24.8 | 46.6 | 53.8 | 42.0 | 37.3 | 34.3 | 39.6 |
| B. OMC requires more time and effort on the part of physicians | 25.6 | 26.3 | 20.4 | 24.7 | 37.2 | 14.3 | 21.0 | 26.3 | 20.3 | 24.2 | 17.4 | 30.8 |
| C. OMC requires more time and effort on the part of the patient | 24.5 | 24.8 | 20.4 | 25.8 | 22.8 | 25.2 | 25.9 | 30.0 | 27.5 | 22.8 | 33.1 | 21.6 |
| D. OMC places a heavy financial burden on the medical institution | 31.5 | 33.0 | 21.8 | 29.0 | 39.3 | 23.8 | 28.5 | 33.8 | 33.3 | 33.4 | 30.5 | 29.6 |
| E. OMC imposes a heavy financial burden on patients | 9.0 | 8.5 | 11.3 | 10.8 | 8.7 | 8.4 | 9.9 | 10.0 | 4.4 | 8.6 | 13.1 | 8.3 |
| F. Difficult for the medical institution to construct a system and communication environment for OMC. | 42.2 | 45.8 | 25.4 | 31.2 | 44.7 | 39.3 | 41.7 | 47.5 | 53.6 | 47.5 | 36.9 | 37.2 |
| G. Difficult for patients to download applications and build a communication environment for OMC. | 56.7 | 57.3 | 57.8 | 52.2 | 51.6 | 61.0 | 59.3 | 61.3 | 65.2 | 59.7 | 58.5 | 51.4 |
| H. Concerns regarding the adequacy of the clinical examination during OMC | 36.4 | 38.9 | 23.9 | 29.6 | 51.3 | 25.7 | 27.3 | 27.5 | 37.7 | 38.9 | 27.1 | 38.6 |
| I. Face-to-face communication is easier than online interaction | 40.0 | 41.4 | 33.8 | 35.0 | 43.5 | 35.8 | 39.1 | 37.5 | 39.1 | 33.9 | 44.1 | 45.0 |
| J. OMC requires patients to visit hospitals for undergoing tests or procedures | 58.4 | 60.1 | 56.3 | 48.4 | 56.3 | 61.9 | 57.9 | 47.5 | 53.6 | 58.2 | 56.8 | 61.1 |
| K. OMC may not be feasible for a larger proportion of patients | 37.7 | 40.0 | 37.3 | 23.1 | 35.4 | 41.4 | 37.4 | 25.0 | 31.9 | 34.9 | 46.2 | 39.3 |
| L. Concerns regarding breach of personal information through OMC | 24.2 | 26.4 | 18.3 | 14.0 | 25.2 | 24.8 | 22.3 | 27.5 | 30.4 | 22.0 | 21.2 | 26.6 |
| M. FMC is preferable to OMC for the education of medical students and young doctors | 16.0 | 16.9 | 15.5 | 10.8 | 18.9 | 16.6 | 12.1 | 22.5 | 20.3 | 15.1 | 13.6 | 16.6 |
| N. Lack of awareness regarding OMC | 22.0 | 21.2 | 26.8 | 23.1 | 22.8 | 28.3 | 15.6 | 31.3 | 18.8 | 22.3 | 22.5 | 20.2 |
| O. Despite being aware, lack of clarity when OMC is appropriate or desired | 24.9 | 24.9 | 24.7 | 24.7 | 22.7 | 30.6 | 22.7 | 31.3 | 34.8 | 25.5 | 20.3 | 23.9 |
| P. Lack of information on OMC providers | 21.0 | 20.8 | 26.8 | 17.7 | 18.5 | 30.6 | 16.0 | 36.3 | 24.6 | 20.6 | 16.1 | 20.9 |
| Q. Because the level of satisfaction with FMC is high and many people do not need OMC | 32.3 | 32.8 | 30.3 | 30.7 | 30.9 | 36.5 | 30.4 | 18.8 | 37.7 | 28.4 | 39.4 | 34.4 |
| R. Regardless of the level of satisfaction with FMC, people tend to maintain the status quo | 20.7 | 20.3 | 18.3 | 24.7 | 19.6 | 23.6 | 19.6 | 31.3 | 29.0 | 20.3 | 22.0 | 18.2 |
HRA, core hospital for medical services in remote areas; HSF, hospital with specific function; CRA, clinics for medical services in remote areas; F-to-F, face-to-face; OMC, online medical care. Yes/No *; “yes” for the facility, but “no” for self. Top three in each category are shown in bold and gray background.
